# Genome assembly of *Stephania longa* provides insight into cepharanthine biosynthesis

**DOI:** 10.3389/fpls.2024.1414636

**Published:** 2024-09-05

**Authors:** Huiying Shang, Yuan Lu, Lulu Xun, Kun Wang, Bin Li, Yuxuan Liu, Tao Ma

**Affiliations:** ^1^ Xi’an Botanical Garden of Shaanxi Province (Institute of Botany of Shaanxi Province), Xi’an, Shaanxi, China; ^2^ School of Ecology and Environment, Northwestern Polytechnical University, Xi’an, China; ^3^ Key Laboratory of Bio-Resource and Eco-Environment of Ministry of Education, College of Life Sciences, Sichuan University, Chengdu, China

**Keywords:** *Stephania longa*, genome, evolution, metabolome, gene expression, cepharanthine

## Abstract

**Introduction:**

*Stephania longa*, a medicinal plant renowned for producing cepharanthine, has gained significance due to the compound's notable antiviral properties against SARS-CoV-2. However, a comprehensive genetic understanding of S. longa has been lacking. This study aimed to develop a high-quality, chromosome-level genome assembly to uncover the genetic intricacies and evolutionary narrative of this species. By integrating genomic data with metabolomic and transcriptomic analyses, we sought to identify key genes involved in cepharanthine biosynthesis.

**Methods:**

We employed a multi-faceted approach comprising genome assembly, phylogenetic analysis, gene family dynamics investigation, metabolomic profiling, and gene expression analysis across various tissues of *S. longa*. This integrated strategy enabled the identification of key genes involved in cepharanthine biosynthesis and elucidated the species’ evolutionary history.

**Results:**

Our phylogenetic analysis clarified the placement of the genus *Stephania* within the Ranunculales order and revealed its notably high mutation rate. We identified gene family expansions and signs of positive selection likely contributing to *Stephania*’s unique metabolic capabilities. Metabolomic profiling uncovered complex regulatory mechanisms orchestrating the biosynthesis and distribution of cepharanthine and related metabolites. Through the integration of genomic, transcriptomic, and metabolomic data, we identified genes with expression patterns and evolutionary trajectories suggesting pivotal roles in cepharanthine biosynthesis, including those involved in crucial biosynthetic steps.

**Discussion:**

This comprehensive study, integrating genomic, metabolomic, and transcriptomic approaches, provides valuable insights into S. longa's biosynthetic potential. It not only enhances our understanding of the species but also establishes a foundation for future investigations into the biosynthesis and therapeutic exploitation of cepharanthine and related alkaloids.

## Introduction

The Menispermaceae family, belongs to the Ranunculales order, comprises 65 genera and approximately 350 species worldwide, predominantly distributed in tropical and subtropical regions. Notably, all plants within the Menispermaceae family contain alkaloids and are recognized for their significant medicinal properties. *Stephania*, a genus belonging to the Menispermaceae family, comprises around 60 species of prominent flowering vines that are native to eastern and southern Asia, as well as Australia (Lo, 1982; Kessler, 1993; Forman, 1997). These plants are distinguished by their unique morphological features, such as large tubers and spirally arranged, peltate leaves where the petiole attaches near the center. This distinctive leaf attachment significantly influences their common name, derived from the Greek word for “crown”, referencing the crown-like arrangement of the anthers in their flowers (Robinson, 1991). These unique adaptations not only provide aesthetic value but also play critical ecological roles in their native habitats, aiding in water retention and efficient sunlight capture. Additionally, the rich alkaloid content in *Stephania* species underscores their importance in traditional medicine, particularly in Chinese herbal practices (Semwal et al., 2010; Wang et al., 2022; Qi et al., 2023).

Previous chemical analyses of *Stephania* species have revealed over 200 alkaloids, which can be categorized into six types based on their chemical structures: morphine dienone, lotus alkane, apocynin, proto-apocynin, proto-cotyledonine, and bisbenzylisoquinoline (Liang et al., 2022; Wang et al., 2022; Qi et al., 2023). These alkaloids not only contribute to the botanical diversity but also offer promising avenues for therapeutic applications including anti-inflammatory, antimicrobial, and antitumor activities (Semwal et al., 2010). Notably, rotundine, l-stepholidine, cepharanthine, and tetrandrine are commonly used as pharmaceutical raw materials. The diverse life forms and chemical strategies observed in *Stephania* suggest a complex evolutionary history. These adaptations may have evolved in response to ecological pressures such as herbivory, competition, and pollination dynamics. The genus’s pivotal evolutionary position within the Menispermaceae family provides valuable insights into the patterns of plant diversification and the development of ecological strategies.

Cepharanthine, a bisbenzylisoquinoline alkaloid produced by *Stephania* species, stands out for its extensive clinical use spanning over 70 years (Bailly, 2019). This clinically approved drug has recently gained prominence for its antiviral properties against SARS-CoV-2, the virus causing COVID-19 (Rogosnitzky and Danks, 2011; Kim et al., 2019; Liang et al., 2022). Research demonstrates cepharanthine’s effectiveness against SARS-CoV-2, showcasing notable results *in vitro* and *in vivo*, particularly in lung tissues (Kim et al., 2019; Ohashi et al., 2021; Zhang et al., 2022; Liang et al., 2023; Xia et al., 2023). Its mechanism in combating COVID-19 is complex, involving interactions with various viral targets and pathways. Advanced network pharmacology techniques have been employed to identify and analyze cepharanthine’s diverse targets, elucidating its role in COVID-19 treatment (Fan et al., 2022; Liu et al., 2022). This includes dissecting protein-protein interaction (PPI) networks and pinpointing hub targets crucial in the drug’s response against the virus (Gerold et al., 2016; Chang et al., 2021). Molecular modeling studies provide insights into how cepharanthine interacts with these targets, including crucial proteins like ACE2, the S1 spike protein of the virus, and other enzymes pivotal in the viral life cycle (Ohashi et al., 2021; Liu et al., 2022). Beyond its antiviral capabilities, cepharanthine is also recognized for its anti-inflammatory and antineoplastic properties, making it a versatile drug for various medical conditions (Rogosnitzky and Danks, 2011; Ershun et al., 2014; Qing et al., 2018; Rogosnitzky et al., 2020). Its long-standing history, coupled with minimal side effects noted in treating different diseases, underscores its potential as a safe and effective therapeutic choice across diverse medical scenarios.

Despite the apparent medicinal significance of cepharanthine, its biosynthetic pathway remains enigmatic. This gap in knowledge is partly attributed to the absence of high-quality genomic resources of the *Stephania* genus. While three genomes have recently been reported for this genus, our understanding of *Stephania* remains limited (Leng et al., 2024; Liu et al., 2024). Addressing this gap, our study presents a comprehensive high-quality genome assembly of *S. longa* complemented by extensive metabolomic and transcriptomic analyses across four different organs of the plant – leaves, roots, fruits, and stems. Through these multifaceted approaches, our study not only illuminates the lineage-specific genetic evolution of *S. longa* but also identifies potential key genes implicated in the biosynthesis of cepharanthine. This research paves the way for a deeper understanding of the genetic underpinnings of this pharmacologically significant alkaloid, opening new avenues for therapeutic exploration and application.

## Results

### Genome assembly and annotation

**Figure 1:**
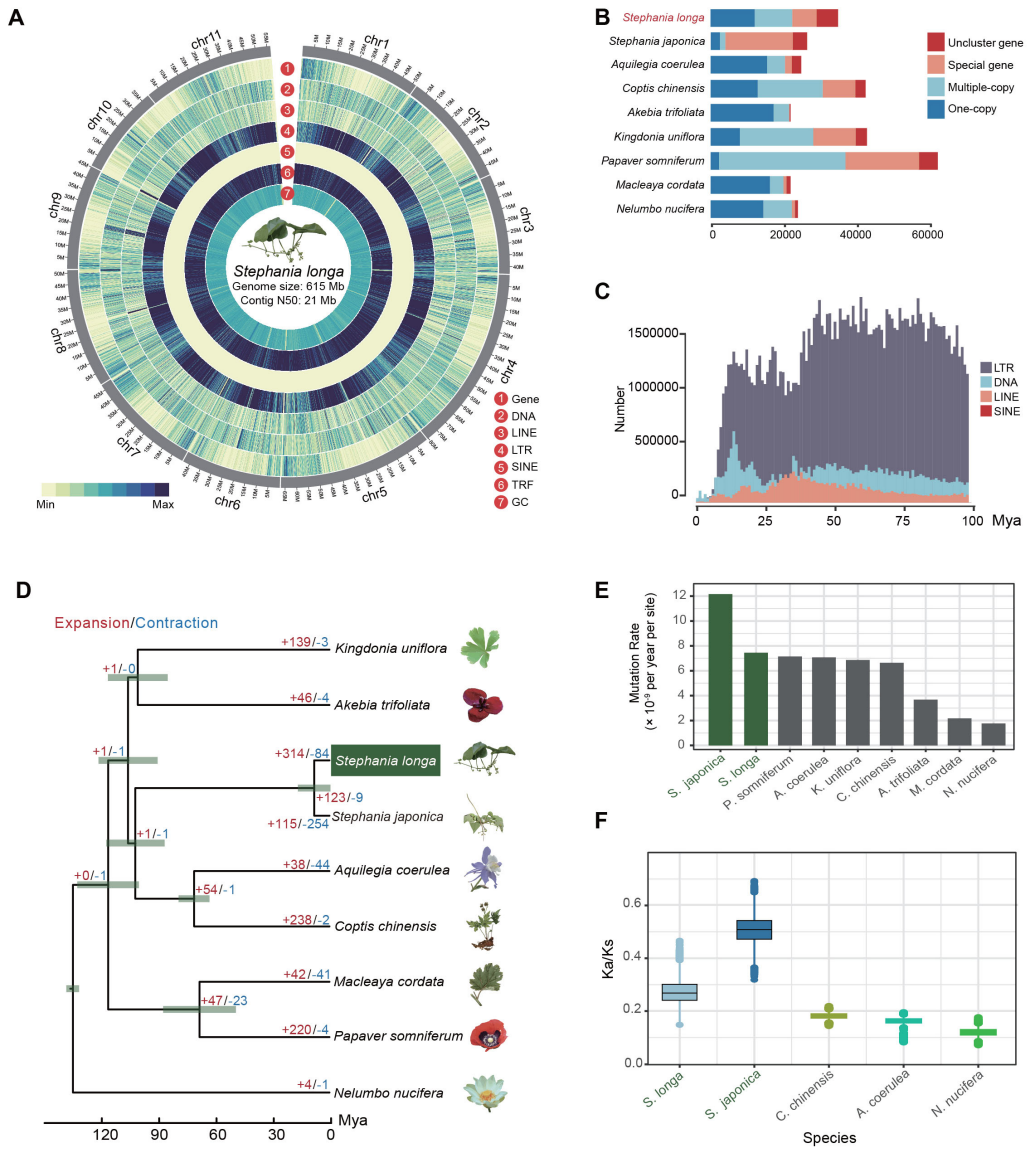
Overview of the *S. longa* genome. **(A)** Circus plot illustrating the 11 chromosomes of the *S. longa* genome, with a genome size of 615 Mb and a contig N50 of 21 Mb. **(B)** Gene number distribution of one-copy, multiple-copy, special genes and unclustered genes in *S. longa* and other species. **(C)** Amplification frequencies of different classes of repetitive sequences in the *S. longa* genome. **(D)** Species tree depicting divergence time and gene family expansion/contraction in various species. **(E)** Mutation rate estimation for each species. **(F)** Ratio of synonymous to nonsynonymous mutations in five species.

We conducted circular consensus sequencing for *S. longa* to achieve a high-quality, chromosome-level genome assembly, resulting in 1.4 million single-molecule reads with an average length of 17 kb and totaling 23 Gb (**Supplementary Table S1**). This was supplemented with 65 Gb of high-throughput chromatin conformation capture (Hi-C) paired-end reads (**Supplementary Table S1**). Based on K-mer analysis, the heterozygosity rate and estimated genome size of *S. longa* were 0.015 and 624.08 Mb, respectively (**Supplementary Figure S1**). By assembling the HiFi sequencing reads, we obtained two haplotype genomes, with sizes of 636.16 Mb for haplotype1 and 621.15 Mb for haplotype2. After removing redundant sequences from the haplotype2 genome using purge_dups v1.2.5, the final genome size is 614.84 Mb, with redundant sequences amounting to 21.32 Mb (**Figure 1A**; **Table 1**). The genome assembly has a contig N50 of 21 Mb (**Supplementary Table S2**), encapsulating 11 pseudo-chromosomes and achieving a 93% anchoring rate (**Supplementary Figure S2**, **Supplementary Table S3**). The genome assembly’s integrity was affirmed by Benchmarking Universal Single-Copy Orthologs (BUSCO) analysis using the embryophyta_odb10 dataset, revealing 97.9% complete BUSCO sequences, with 1.1% partially present and 1.0% missing, underscoring both the contiguity and completeness of our assembly (**Supplementary Table S4**).

**Table 1:** Summary of *S. longa* genome assembly and annotation.

Assembly features	*S. longa’*s genome
Sequence number	397
Total length (bp)	614,840,797
Max scaffold (bp)	80,332,011
Scaffold N50 (bp)	49,162,663
Contig N50 (bp)	21,223,701
Repeat content (%)	67.96
Gene number	34,951
Average gene length (bp)	4239.51
Average CDS length (bp)	322.08
BUSCO (genome)	97.9%
BUSCO (gene)	96.2%

A total of 68% (418Mb) of the assembly were annotated as repetitive sequences, of which 31.34% are long terminal repeat (LTR) retrotransposons (**Supplementary Table S5**). The most abundant subclass of LTR is Gypsy and Copia, accounting for 19.67% and 10.78%, respectively. In contrast to LTR, the DNA transposable elements (5.93%) and the long interspersed nuclear elements (LINE) (2.71%) are not that abundant (**Supplementary Table S5**). Moreover, we noticed that 29.72% of the genome sequences were annotated as the type of “Unknown”, reflecting species-specific genome expansion (**Figure 1B**; **Supplementary Table S5**). We further counted the amplification times of each class of repetitive sequences and found that they have mainly appeared in the last few tens of millions of years, with no outbreaks in the recent past, which is also consistent with the relatively modest size of their genomes (**Figure 1C**).

We further applied an integrated strategy including homolog-based and *ab initio* gene prediction methods to annotate the protein-coding genes and obtained a set of 34,951 protein-coding genes (**Supplementary Table S6**). A total of 28,629 genes are functionally annotated, of which 14,648 were annotated by Gene Ontology database and 17,206 were annotated by KEGG database (**Supplementary Table S6**). The gene density across different chromosomes is ranged from 51.47 per Mb to 58.84 per Mb (**Supplementary Table S7**). The average gene length of protein-coding genes is 4,239.51 bp, and the average coding sequences length is 987.95 bp, with an average of 4.23 exons per genes (**Supplementary Table S8**). We further estimated the tissue-specific gene expression quantity for each gene and found 24,791 genes are expressed in at least one tissue, 17,004 genes are expressed in all tissues (TPM > 1) (**Supplementary Table S9**).

### Phylogeny relationship and evolutionary rate of genes in *Stephania longa*

To elucidate the evolutionary relationship of *S. longa*, we commenced by pinpointing 1,939 single-copy orthologous genes across eight species (including *S. longa*, *Stephania japonica*, *Aquilegia coerulea*, *Coptis chinensis*, *Akebia trifoliata*, *Kingdonia uniflora*, *Papaver somniferum* and *Macleaya cordata*) from the Ranunculales order, using *Nelumbo nucifera* from the Proteales order as an outgroup. These genes were amalgamated to construct a maximum likelihood phylogenetic tree, complemented by a species tree derived through a two-step coalescence approach. Both trees exhibited identical topologies with 100% bootstrap support, identifying *S. longa* and *S. japonica* as the most divergent species and positioning them as a sister clade to *C. chinensis* and *A. coerulea* (**Figure 1D**). Despite that, our exploration into the phylogenetic discordance among these orthologous genes unveiled a notable degree of incomplete lineage sorting in their early divergence phases (**Supplementary Figure S3**). This result reaffirms that relying on a single gene, or a small number of genes, does not provide an accurate reflection of the phylogenetic relationships among species. It is only through whole-genome phylogenetic reconstruction that we can accurately determine these relationships. Overall, the analysis of 1,939 gene trees revealed a diverse set of 203 distinct topologies (**Supplementary Table S10**). Each topology received varying levels of support from the gene trees. Notably, one topology stood out with the highest support, aligning closely with the species tree and being supported by 177 gene trees. Additionally, we observed that 67 gene trees supported a topology where *K. uniflora* is more closely related to a clade comprising *S. longa*, *S. japonica*, *C. chinensis*, and *A. coerulea* rather than to *A. trifoliata*. Conversely, 62 gene trees favored a topology where *K. uniflora* is closer to the two *Stephania* species (**Supplementary Table S10**).

Heightened mutation rates were found in two *Stephania* species compared to other species, estimated at 7.14×10^-9^ and 1.21×10^-8^ mutations per site per year for *S. longa* and *S. japonica*, respectively (**Figure 1E**; **Supplementary Table S11**). Further analysis of the ratio of synonymous to nonsynonymous mutations relative to their ancestral nodes revealed higher Ka/Ks ratios in the two *Stephania* species, particularly in *S. japonica* (**Figure 1F**). The high mutation rate, coupled with a high ratio of synonymous to nonsynonymous mutations, indicates that these species possess significant genetic diversity and are under strong purifying selection to maintain essential protein functions. This balance provides potential adaptability while ensuring genomic stability. Moreover, we identified a total of 312 genes in the common ancestor of the two *Stephania* species that exhibit strong signals of positive selection (**Supplementary Table S12**). These genes are likely to play a significant role in the biosynthesis of lineage-specific secondary metabolites (**Supplementary Table S13**).

### Whole genome duplication (WGD) and dynamics of gene family evolution

**Figure 2:**
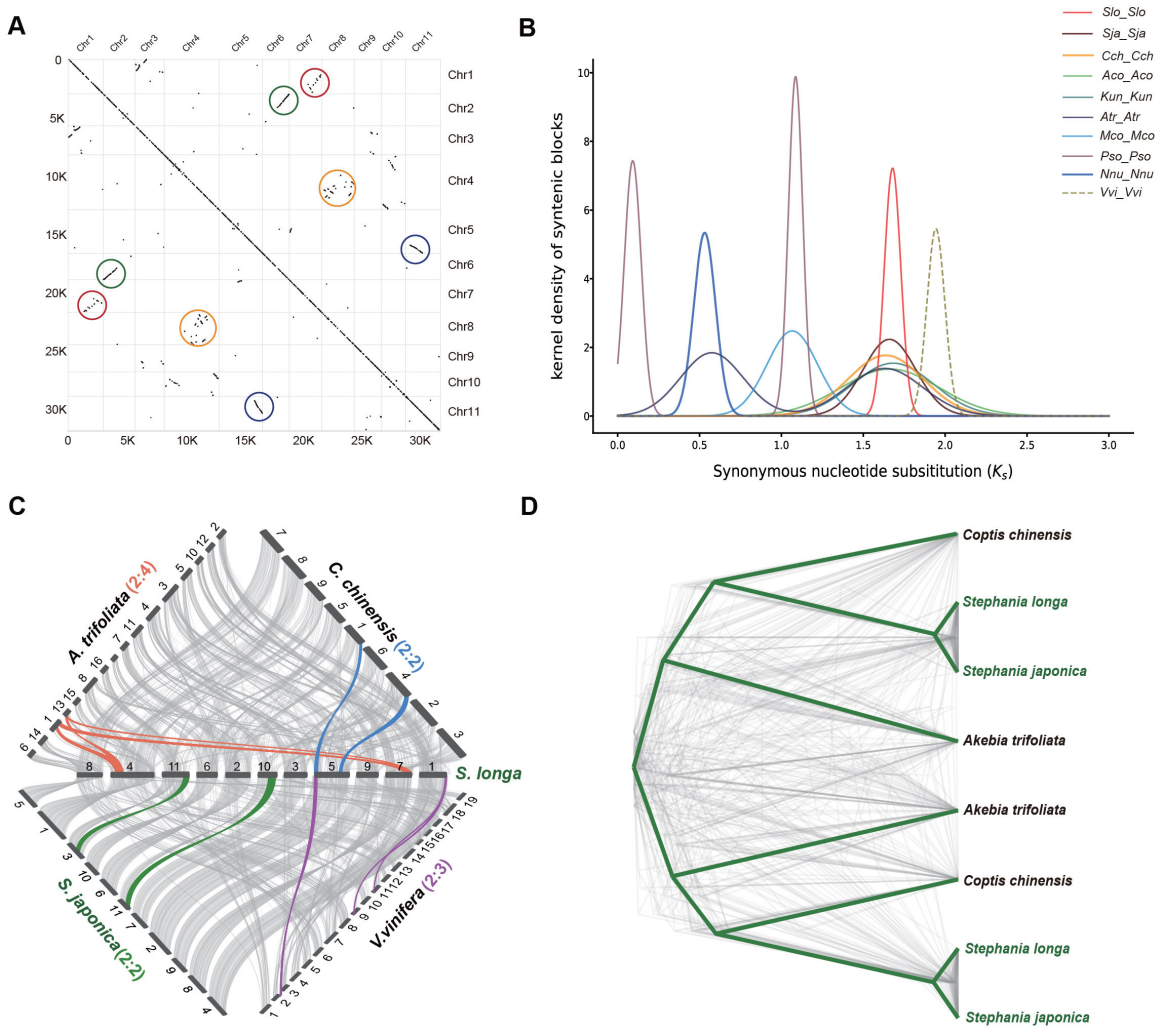
Identification of WGD in the *S. longa* genome. **(A)** Intragenomic collinear blocks of *S. longa* chromosomes, highlighting collinearity regions resulting from WGD events (indicated by colored circles). **(B)** Distribution of synonymous substitution rates (Ks) for paralogous collinear blocks in the genomes of *S. longa* (*Slo_Slo*), *S. japonica* (*Sja_Sja*), *C*. *chinensis* (*Cch_Cch*), *A*. *coerulea* (*Aco_Aco*), *K*. *uniflora* (*Kun_Kun*), *A*. *trifoliata* (*Atr_Atr*), *M. cordata* (*Mco_Mco*), *P. somniferum* (*Pso_Pso*), *N. nucifera* (*Nnu_Nnu*) and *V. vinifera* (*Vvi_Vvi*). **(C)** Syntenic blocks among *S. longa*, *S. japonica*, *C*. *chinensis*, *A. trifoliata* and *V. vinifera*. Chromosome order is represented by numbers in the original genome sequence and each line represents one block. Syntenic blocks are marked with gray lines, while lines in other colors indicate examples of syntenic relationships between *S. longa* and related species, corresponding to WGD or whole-genome triplication (WGT) events. **(D)** Phylogenetic trees constructed for genes within the shared collinear blocks among four Ranunculales species.

In our investigation of the genome duplication process in *S. longa*, we conducted a collinearity comparison of its genome using MCScanX toolkit. We observed extensive collinear relationships between certain chromosomes, such as between chr2 and chr6, as well as chr5 and chr11. Additionally, more common but shorter collinear relationships were found between other chromosomes, such as between chr1 and chr7, and between chr8 and chr4 (**Figure 2A**). These collinear relationships are indicative of ancestral whole-genome duplication remnants. However, the limited prevalence of these collinear relationships suggests that the whole-genome duplication event took place in the distant past, followed by a prolonged period of diploidization during the course of evolution.

Assessing whether the WGD event in *S. longa* was a shared event with its close relatives or unique to *S. longa*, we calculated the Ks distribution of paralogous collinear blocks within each species. We found that the Ks peak in *V. vinifera* is the oldest, consistent with the fact that this species has experienced one ancient WGT event. Similar Ks values were found in *S. longa*, *S. japonica*, *C. chinensis*, *A. coerulea*, *K. uniflora* and *A. trifoliata*, indicating that these six Ranunculales species shared a common WGD event. Additionally, the presence of two Ks peaks in *A. trifoliata* suggests that this species underwent an additional recent WGD event. To further substantiate this, we conducted a collinearity comparison of the genome of *S. longa* with three Ranunculales species and *V. vinifera. *(**Figure 2C**). The ratio of collinear regions between *S. longa* and *V. vinifera* was 2:3, indicating the occurrence of one WGD event in *S. longa*. The ratio of collinear regions between *S. longa* and both *C. chinensis* and *S. japonica* was 2:2, suggesting a shared WGD event among these species. In contrast, the ratio between *S. longa* and *A. trifoliata* was 2:4, indicating that *A. trifoliata* experienced two WGD events. Similarly, phylogenetic trees constructed for genes within the shared collinear blocks among these species also support the shared whole-genome duplication event for the four Ranunculales species (**Figure 2D**; **Supplementary Table S14**). These findings suggest that the genus *Stephania* did not undergo whole-genome duplication independently, and its unique adaptability may primarily be driven by the independent expansion of certain gene families.

**Figure 3:**
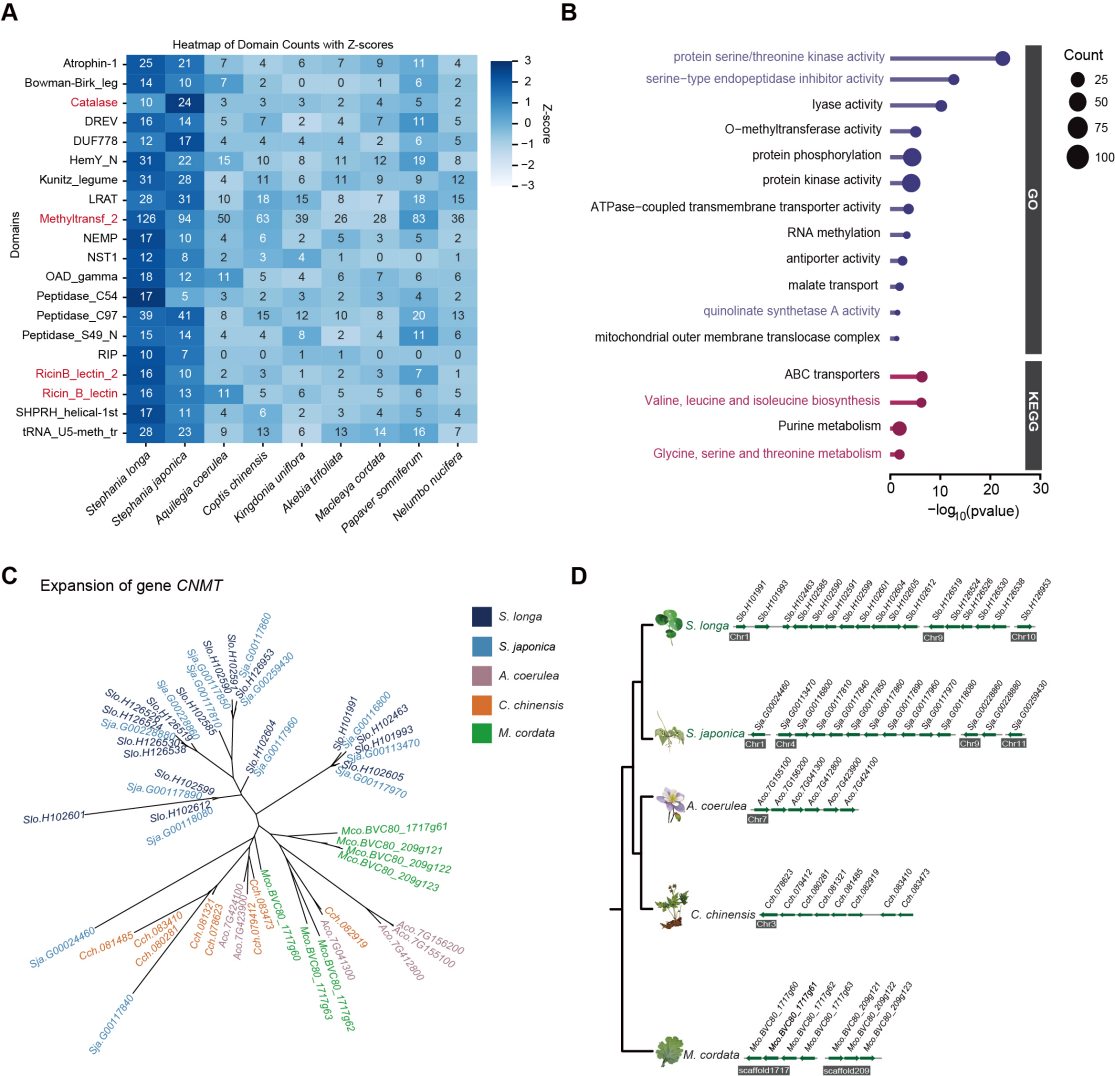
Expansion of gene families in the genus *Stephania*. **(A)** Twenty significantly expanded domains in the genus *Stephania*. **(B)** Gene Ontology (GO) and KEGG pathway analysis of the expanded gene families in the genus *Stephania*. **(C)** Phylogeny of the expanded gene *CNMT* in *S. longa* and *S. japonica* compared to other three species. **(D)** Genomic distribution of *CNMT* gene copies in different species.

To identify which specific gene family dynamics are altered in the genus *Stephania*, we first scanned the copy number of each protein domain using the Pfam-A database in *S. longa* as well as in eight other related species. In total, we identified 105,142 domains in *S. longa* and *S. japonica*, respectively. a number close to that of most other species, except for *Papaver somniferum* which is significantly more (235,539), but this is not surprising given its additional whole-genome duplication events (**Figure 2B**; **Supplementary Table S15**). In total, we identified 247 protein domains in *Stephania* with significantly higher numbers, some of which are potentially associated with the synthesis of secondary metabolites (**Supplementary Table S16**). Notably, the significant expansion of domains like Catalase (n=10), Methyltransf_2 (n=126), DAHP_synth_2 (n=15), RicinB_lectin_2 (n=16), and Ricin_B_lectin (n=16) might be intricately linked to its ability to synthesize cepharanthine, an isoquinoline alkaloid (**Figure 3A**).

We further utilized the CAFE (Computational Analysis of gene Family Evolution) software for a detailed examination of genes that have experienced specific expansions and contractions. Our analysis revealed 314 gene families with notable expansion and 84 with contraction in the genus *Stephania* (**Figure 1D**). We found that the contracted gene families did not exhibit significant enrichment in any GO categories or KEGG pathways. In contrast, the expanded gene families showed a marked enrichment in pathways related to the biosynthesis of the branched-chain amino acids valine, leucine, and isoleucine, alongside purine metabolism pathways (**Figure 3B**). A particularly noteworthy observation was that some of the genes in the isoquinoline alkaloid biosynthesis, notably including *GOT2*, *TAT*, and *CNMT*, exhibited multiple copies in comparison to closely related species (**Supplementary Table S17**). Of note is the presence of the Methyltransf_2 protein domain in multiple copies of the *CNMT* gene (**Figures 3C, D**), underscoring the consistency between different independent analyses.

### Comparative metabolite profiling across four organs

**Figure 4:**
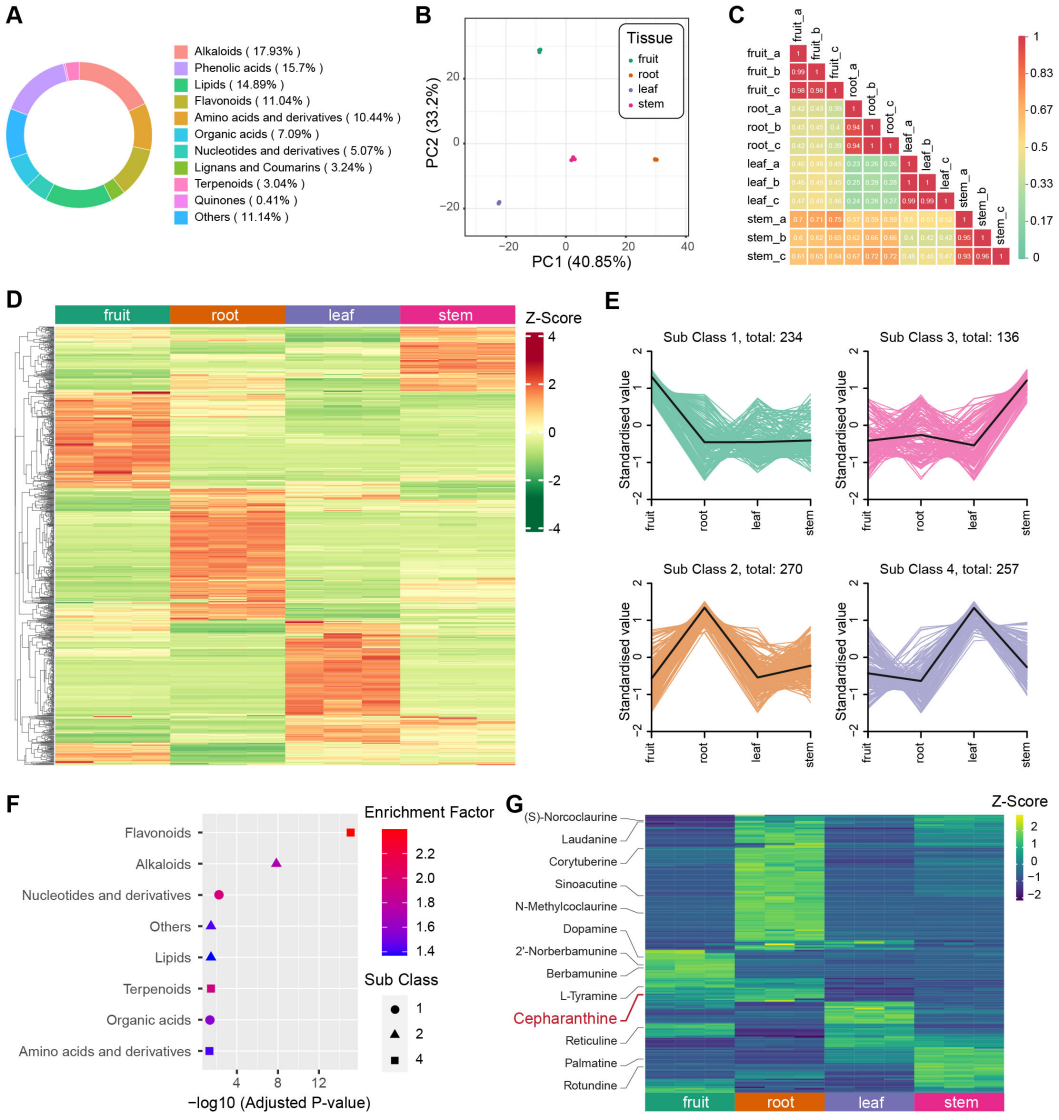
Metabolite analysis of different tissues in *S. longa*. **(A)** Composition and proportions of metabolites in *S. longa*. **(B)** Principal Component Analysis (PCA) plot of metabolites in root, stem, leaf, and fruit. **(C)** Correlation analysis among different tissues. **(D)** Heatmap illustrating differential metabolite clustering among different tissues. **(E)** K-means analysis of differential metabolites among different tissues. **(F)** Enrichment analysis of metabolites classified by K-means analysis. **(G)** Heatmaps depicting the expression levels of alkaloids across different tissues. Notably, different types of alkaloids exhibit variations in expression, with Cepharanthine showing high expression in the roots.

To determine the predominant metabolites present in different tissues of *S. longa*, we employed Ultra-Performance Liquid Chromatography coupled with Tandem Mass Spectrometry (UPLC-MS/MS) for the quantification of metabolite content in the fruit, root, leaf, and stem tissues, each of which included three replicates. We identified 987 metabolites across these tissues, and the most abundant metabolites are alkaloids (17.93%), phenolic acids (15.7%) and lipids (14.89%) (**Figure 4A**). Additionally, we evaluated the Coefficient of Variation (CV) for each sample. The findings indicated exceptional stability in the experimental data, with over 85% of metabolites showing a CV below 0.3, and more than 93% displaying a CV under 0.5 (**Supplementary Figure S4**). Principal Component Analysis (PCA) was conducted for all samples based on each metabolite’s concentration, revealing a consistent clustering of the three replicates within each tissue type (**Figure 4B**). We also computed the Pearson’s correlation coefficient for each sample, finding a high correlation among the three replicates of each tissue. These findings underscore the high repeatability and reliability of our data (**Figure 4C**).

In our comprehensive metabolite analysis of each biological sample, we applied hierarchical clustering to identify patterns among various compounds. The result revealed distinct metabolite profiles unique to each tissue type (**Figure 4D**). Further, using K-means clustering, we categorized these profiles into four subclasses, highlighting cases where specific organs showed elevated concentrations of certain compounds (**Figure 4E**). For instance, leaves exhibited a predominance of flavonoid-type metabolites, roots were abundant in alkaloids, and fruits displayed elevated levels of nucleotides and their derivatives (**Figure 4F**). By combining OPLS-DA analysis with fold change criteria, we identified significant differences in the relative abundance of metabolites among distinct tissue types. Consistent with our previous observations, a substantial proportion of the metabolites displayed notable variations when comparing tissue pairs (**Supplementary Figures S5**-**S10**). These distinctive metabolites notably contribute to various KEGG pathways, including isoquinoline alkaloid biosynthesis (ko00950), alpha-linolenic acid metabolism (ko00592), and nicotinate and nicotinamide metabolism (ko00760) (**Supplementary Tables S18**-**S23**).

Cepharanthine belongs to the class of isoquinoline alkaloids, has its highest concentration in root tissues, a finding corroborated with previous knowledge that it is predominantly extracted from roots (**Figure 4G**). Despite the unknown biosynthesis pathway of cepharanthine, we investigated the presence of other isoquinoline alkaloids involved in the KEGG pathway of isoquinoline alkaloid biosynthesis. Intriguingly, we observed variations in the concentration of these alkaloids across different tissues, such as fruits, leaves, and stems (**Figure 4G**). This suggests a tissue-specific utilization of this biosynthetic pathway, leading to a diverse range of end products.

### Genes with similar expression pattern with cepharanthine

**Figure 5:**
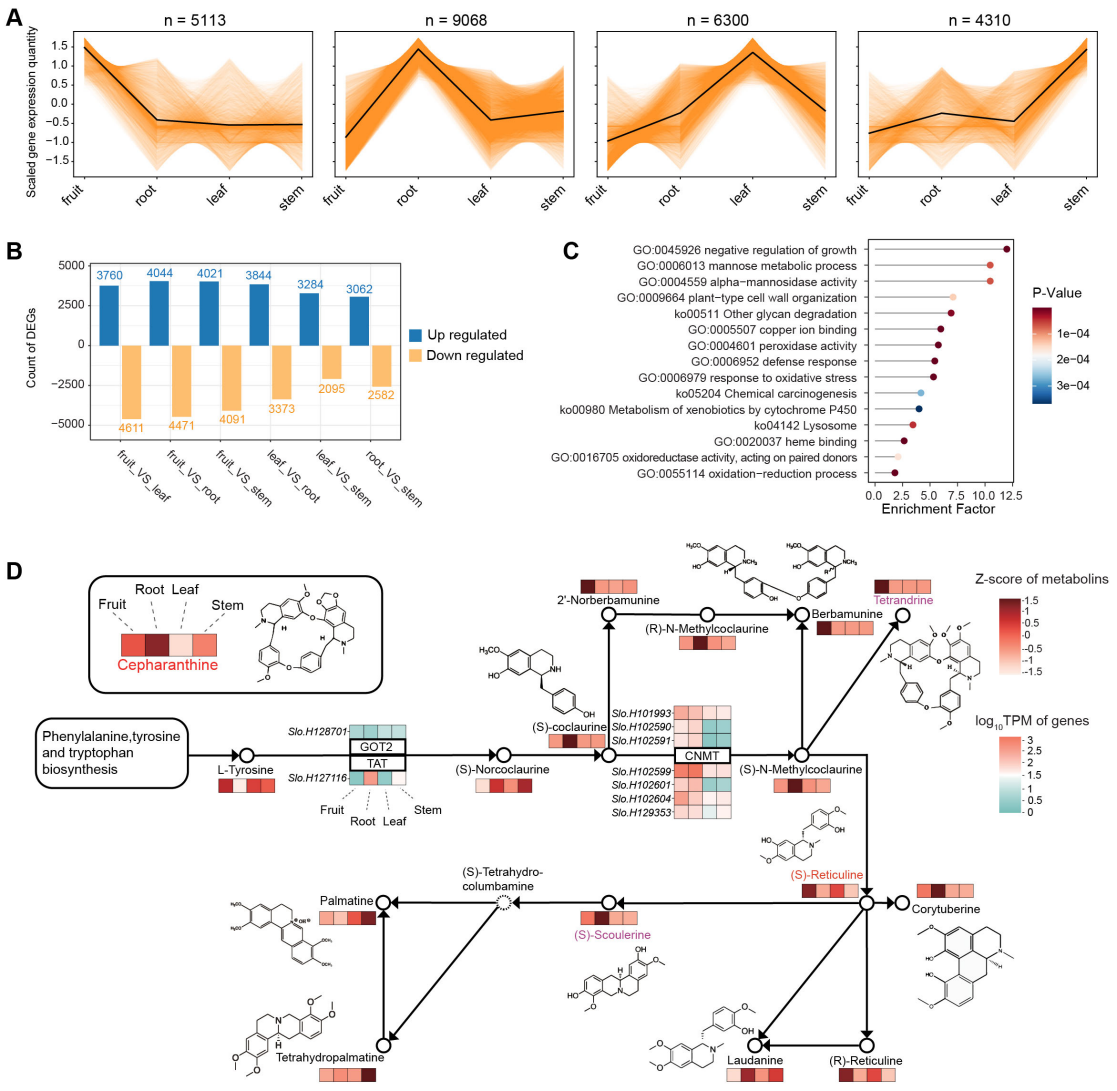
Transcriptome analysis of different tissues in *S. longa*. **(A)** K-means analysis of expression levels across different tissues. **(B)** Differentially expressed genes among various comparisons. **(C)** Gene Ontology (GO) terms and KEGG pathways of genes differentially expressed between the root and other tissues. **(D)** Gene expression profiles associated with the isoquinoline alkaloid biosynthesis pathway.

We then examined the overall gene expression pattern of various tissues for searching the related gene expression pattern to synthesis of cepharanthine. Utilizing K-means analysis, we found that a substantial number of genes (9,068) exhibit higher expression in the root compared to other tissues (**Figure 5A**). This pattern aligns with our metabolite profiling data, which also indicates a higher concentration in the root for most metabolites. Our investigation further revealed differentially expressed genes (DEGs) between tissue types, averaging about 7,000 DEGs for each tissue pair (**Figure 5B**). Notably, 1,047 genes were identified as being significantly more expressed in the root than in any other tissue. These genes are predominantly involved in a variety of biological processes and pathways, as indicated by their enrichment in numerous Gene Ontology (GO) terms and KEGG pathways (**Figure 5C**). These include the negative regulation of growth, mannose metabolism, and defense response, among others. It should be noted that the unique expression profile of these genes in the root likely contributes to their involvement in diverse functions, extending beyond just alkaloid synthesis.

To elucidate the genetic underpinnings of cepharanthine biosynthesis, we examined gene expression profiles associated with the isoquinoline alkaloid biosynthesis pathway (**Figure 5D**). Our findings reveal intriguing connections between the expression patterns of expanded genes in *S. longa* and cepharanthine synthesis, highlighting their crucial roles in metabolic pathways. Particularly noteworthy is the observation that seven paralogs of the *CNMT* gene demonstrate significant expression levels in both the roots and fruits of *S. longa* (**Figure 5D**). This suggests their involvement in key stages of cepharanthine biosynthesis. Additionally, the elevated expression of a specific *TAT* gene copy in the roots provides further evidence of a specialized metabolic machinery in *S. longa* dedicated to cepharanthine synthesis.

Besides to the expanded genes, we also noted that some positively selected genes are also highly expressed in the root (**Supplementary Table S24**). These genes also warrant particular attention for their potential roles in the synthesis of cepharanthine. For instance, *XYP2*, which is related to xyloglucan endotransglucosylase/hydrolase activities in other plant models such as *Arabidopsis thaliana* (Motose et al., 2004; Kobayashi et al., 2011), might play a role in cell wall remodeling and polysaccharide metabolism in the roots of *S. longa*. *EMB2729*, known to be essential for embryo development in *Arabidopsis*, with its specific function in cell division and differentiation (Tzafrir et al., 2003; Wang et al., 2010; Meinke, 2020), could have a significant role in root development and the differentiation of root cells specialized in the synthesis of cepharanthine in *S. longa*.

Finally, it should be noted that the observed variations in expression levels of metabolites closely associated with cepharanthine, such as berbamunine, tetrandrine, (S)-scoulerine, and palmatine across different tissues, underscore the complexity of the biosynthetic pathways in *S. longa* (**Supplementary Figure S11**). For instance, berbamunine and tetrandrine exhibit their highest expression in fruits, while (S)-scoulerine is predominantly found in roots. This differential expression pattern suggests a sophisticated regulatory mechanism that directs the flow of precursors and intermediates to specific metabolites based on the tissue type. Given the structural similarities between tetrandrine and cepharanthine, particularly the subtle variations in their CH3 groups, it’s plausible that tissue-specific gene expression plays a crucial role in channeling common precursors towards the synthesis of these distinct but chemically related metabolites. This nuanced control mechanism may ensure the optimal production of each metabolite, contributing to the plant’s adaptability and survival by maximizing the diversity of its chemical defenses and interactions with the environment.

## Discussions

Our comprehensive study on *S. longa* not only unveils the high-quality genome assembly of this medicinal plant but also provides significant insights into the biosynthesis of cepharanthine, a compound with notable antiviral properties, particularly against SARS-CoV-2. Through detailed phylogenetic analyses, we have elucidated the evolutionary positioning of *S. longa* within the Ranunculales order, highlighting its rapid mutation rate. Based on the analysis of gene evolutionary rates, we identified 312 genes under positive selection in the genus *Stephania*. Although these genes are not significant enriched in any functional categories or pathways, some of them might have been contributed to the lineage specific unique secondary metabolite. Based on our collinearity comparison with four other species, *S. japonica, A. trifoliata*, *C. chinensis*, and *V. vinifera*, our findings suggest that *S. longa* did not undergo independent WGD event, but rather shared a common WGD event with other Ranunculales species. The investigation into gene family dynamics has uncovered significant expansions and positive selection events that potentially contribute to the plant’s specialized metabolic capabilities, providing valuable insights into the genetic basis of cepharanthine synthesis. Among these genes, catalase stands out as a potential player in managing oxidative stress, an inherent byproduct of the complex alkaloid biosynthesis pathway. Its presence suggests a mechanism for counteracting the detrimental effects of reactive oxygen species during cepharanthine production. The expansion of the Methyltransf_2 gene family is particularly noteworthy, implying its critical function in the methylating precursors or intermediates involved in the cepharanthine pathway. This gene family likely plays a vital role in modifying specific compounds, thereby influencing the synthesis of cepharanthine. Another gene of interest is DAHP_synth_2, known for its involvement in aromatic amino acid synthesis (Webby et al., 2005). Its presence suggests that it may provide essential precursors necessary for the production of cepharanthine. Furthermore, the expanded gene families containing RicinB lectin domains indicate their potential involvement in glycosylation processes or cellular interactions that facilitate alkaloid production (Rüdiger and Gabius, 2001). This expansion reveals an evolutionary adaptation that enhances the plant’s capacity to synthesize cepharanthine, either directly through glycosylation or by creating a favorable biochemical environment.

We have performed metabolomic profiling and analyzed gene expression levels in various tissues of *S. longa*. This integrated approach has provided a holistic view of the plant’s metabolic landscape, allowing us to identify key genes with specific expression patterns and evolutionary signatures that suggest their involvement in cepharanthine biosynthesis. The *CNMT* gene, which stands for Caffeic acid O-Methyltransferase, plays a critical role in the lignin and melatonin synthesis pathways in plants (Louie et al., 2010; Byeon et al., 2014). This gene is characterized by a conserved C-terminal catalytic domain known as Methyltransf_2, which includes a SAM/SAH (S-Adenosyl methionine/S-Adenosyl homocysteine) binding pocket and a substrate-binding site. The presence of these domains indicates the gene’s involvement in methylation processes, which are crucial for modifying various compounds within the plant, including secondary metabolites like alkaloids. Therefore, it is plausible that *CNMT* is involved in the biosynthesis of cepharanthine by contributing to the methylation steps necessary for its formation.

*TAT*, or tyrosine aminotransferase, is involved in the initial steps of the tyrosine-derived pathway, which is fundamental to the biosynthesis of a wide array of plant secondary metabolites, including isoquinoline alkaloids (Schenck and Maeda, 2018; Xu et al., 2020). These findings collectively suggest that the expansion and high expression of specific genes like *CNMT* and *TAT* in *S. longa* are not merely coincidental but are likely integral to the plant’s evolutionary strategy to synthesize and accumulate significant levels of cepharanthine. This adaptation may offer *S. longa* a competitive edge in its ecological niche, possibly through enhanced defensive capabilities or other ecological benefits conferred by the presence of cepharanthine. Further functional studies on these expanded and highly expressed genes will be crucial to fully understand their roles in the biosynthesis and regulation of cepharanthine in *S. longa*.

The root-specific expression patterns of these genes suggest their potential involvement in crucial processes related to the plant’s adaptation to its environment, possibly through the production of secondary metabolites such as cepharanthine. This adaptation is essential for root growth and response to environmental stresses, potentially influencing the biosynthesis of secondary metabolites. The positive selection observed in the *XYP2* gene indicates an evolutionary adaptation that could enhance the plant’s ability to modulate its cell wall structure, favoring increased secondary metabolite production like cepharanthine. This adaptation is particularly significant in response to environmental challenges or microbial interactions in the soil. The high expression of *EMB2729* in roots, coupled with its positive selection, suggests its involvement in optimizing root cell functions to enhance the production of secondary metabolites. This could involve the regulation of metabolic pathways specific to root tissues or the development of specialized cells within the roots that are highly efficient in synthesizing cepharanthine. These adaptations contribute to the overall capacity of the plant to produce this valuable alkaloid. These findings highlight the vital role of these genes in the plant’s ability to produce cepharanthine and provide a compelling direction for future research into the genetic and biochemical basis of this plant’s unique secondary metabolite profile. Understanding these adaptations will deepen our knowledge of the mechanisms underlying cepharanthine biosynthesis and may have implications for potential applications in agriculture, medicine, or other fields. Despite our progress, a detailed functional analysis of the identified genes and their roles in the cepharanthine biosynthesis pathway remains essential. Future studies will need to employ advanced techniques such as gene editing, functional genomics, and metabolic engineering to validate the functions of these genes and elucidate the step-by-step synthesis of cepharanthine in the genus *Stephania*.

## Methods

### Plant materials and genome sequencing

The plant specimen was collected from Hulu Mountain, Nan’ao Island, Shen’ao Town, Shantou City, Guangdong Province, China (latitude 23°24’54.0”N, longitude 117°03’25.2”E). Genomic DNAs were collected from fresh leaves using a plant genomic DNA kit (QIAGEN, Shanghai, China). The genomic DNA extracted was used for library construction following the PacBio SMRT library construction protocol. This involved DNA fragmentation, DNA concentration, damage repair, end repair, adapter ligation and template purification. PacBio HiFi sequencing libraries were generated using the NEB Next^®^ Ultra™ DNA Library Prep Kit (NEB, USA) and prepared following the standard manufacturer’s protocol (Pacific Biosciences, CA, USA). Subsequently, the genome was sequenced on the PacBio Sequel II platform (Pacifc Biosciences, CA, USA).

For genome survey analysis and genome polishing, a short paired-end Illumina DNA library was sequenced on the HiSeq 2000 platform using a 150 bp paired-end strategy, following the manufacturer’s instructions (Illumina, San Diego, CA, USA). HiFi reads were produced using the PacBio Sequel II system. The Hi-C library construction followed standard procedures and was sequenced on the DNBseq T7 platform in PE150 mode.

### Genome assembly

To do the genome survey of *S. longa*, we first performed preprocessing on the Illumina sequencing reads. This involved filtering out adapter sequences, removing low-quality reads, and trimming using Fastp v0.23.4 (Chen, 2023). We then utilized the K-mer method with Jellyfish v2.2.3 software (Marçais and Kingsford, 2011) and a k-mer size of 21 for the estimation of genome size and heterozygosity using GCE v1.0.2 (https://github.com/fanagislab/GCE) (Liu et al., 2013). The HiFi reads were then employed for contig assembly using Hifiasm v0.16.1 (Cheng et al., 2022) with default parameters. We then utilized purge_dups v1.2.5 (https://github.com/dfguan/purge_dups) to analyze haplotigs and overlaps in the assembly based on read depth, followed by nextPolish (https://github.com/Nextomics/NextPolish) for base correction, resulting in haploid contig versions of the genome sequence corrected by two third-generation sequencing datasets. To further enhance the assembly, we utilized Fastp v0.23.4 (Chen, 2023) to filter Hi-C sequencing reads aligned to the contig assembly with BWA v0.7.17 (Li, 2013). The resulting draft reference genome was scaffolded using YaHS v1.1a-r3 (Zhou et al., 2023). To evaluate the completeness of the *S. longa* genome, we employed BUSCO v5.4.0 (Manni et al., 2021) using a set of 1,614 conserved genes sourced from the embryophyta_odb10 database.

### Annotation of repetitive sequences

To annotate repetitive sequences within the genome of *S. longa*, we utilized a combination of *ab initio* and homology-based approaches. For *ab initio*-based repeat identification, we employed LTR_Finder v1.05 (Xu and Wang, 2007), RepeatScout v1.05 (Price et al., 2005), and PILER v2.4 (Edgar and Myers, 2005). To classify homology-based repeat elements, we used RepeatMasker v4.07 and RepeatProteinMask v4.0.7 (Tarailo-Graovac and Chen, 2009), which were employed to search against the Repbase v21.12 repeat sequence database (Bao et al., 2015).

### Prediction and annotation of protein coding genes

To predict protein-coding genes, we utilized a combination of three methods: *de novo* prediction, homology-based prediction, and transcript-based prediction. For *de novo* prediction, we employed Augustus v2.5.5 (Stanke and Waack, 2003). Homology-based evidence was obtained by aligning protein sequences of *Aquilegia coerulea*, *Coptis chinensis*, *Akebia trifoliata*, *Kingdonia uniflora*, *Papaver somniferum*, *Macleaya cordata*, *and Nelumbo nucifera* against the *S. longa* assembly using BLAST, with an e-value threshold of 1e^−5^.

For transcriptomic prediction, we used SPAdes v3.15.5 (Prjibelski et al., 2020) to perform assembly based on the reference transcripts. Gene prediction was carried out using TransDecoder v.5.5.0 (available at https://github.com/TransDecoder/TransDecoder/releases/tag/TransDecoder-v5.5.0). Gene structures were predicted using GeneWise v2.2.0 (Birney et al., 2004). Finally, the prediction results obtained from the three methods were merged to generate a consensus gene set using EVidenceModeler v2.0.0 (Haas et al., 2008).

To perform gene functional annotation, we employed a consensus of sequence and domain information. Protein sequences were aligned to NCBI Non-Redundant Protein Sequence (NR) databases, Kyoto Encyclopedia of Genes and Genomes (KEGG v89.0; Kanehisa et al., 2017), SwissProt, and TrEMBL (Uniprot release 2020-06; Boeckmann et al., 2003) using BLASTp. Domains were searched and predicted using InterProScan v5.11-55.0 (Zdobnov and Apweiler, 2001) with publicly available databases including PANTHER (Thomas et al., 2003), Pfam (Bateman et al., 2004), PRINTS (Attwood et al., 2000), ProDom (Servant et al., 2002), PROSITE profiles (Sigrist et al., 2010), and SMART (Letunic et al., 2012). Gene ontology (GO) terms (Ashburner et al., 2000) for each gene were predicted based on the InterPro descriptions.

### Phylogenetic tree inference and estimation of divergence times

To explore the evolutionary history of the *S. longa* genome, we obtained eight additional sequenced genomes from NCBI for conducting multispecies alignments. These genomes include *S. japonica*, *A. coerulea*, *C. chinensis*, *A. trifoliata*, *K. uniflora*, *P. somniferum*, *M. cordata*, and *N. nucifera*. Pairwise comparisons of the gene sets among these nine species were performed using the diamond software v2.0.4.142 (Buchfink et al., 2021). Genes with an E-value smaller than 1e^-5^ in the alignment results were subjected to the Reciprocal Best Hit (RBH) method to identify the 1:1:1 orthologous gene dataset across these species. For each orthologous gene set, we utilized the Muscle software v5.1 (Edgar, 2022) to obtain well-aligned protein sequences. These alignments were then concatenated and used as the input file for RAxML v8.2.12 (Stamatakis, 2014) to calculate the maximum likelihood phylogenetic tree. The parameters used for RAxML were as follows: -f a –m PROTGAMMAAUTO -p 15256 -x271828 -N 100. Additionally, employing the same parameters, we computed the maximum likelihood tree for each orthologous gene set. These individual trees were combined to create the input file for ASTRAL v5.7.8 (Zhang et al., 2018), which generated the final species tree.

To process the aligned protein sequences mentioned earlier, along with their corresponding CDS sequences, we utilized the pal2nal software v14 (Suyama et al., 2006) to convert the protein alignment results into nucleotide sequences aligned by codons. The concatenated nucleotide sequences were then used to extract all four-fold degenerate sites located at the 3rd codon position. These extracted four-fold degenerate sites were employed as the input file for the mcmctree program in the PAML software package v4.9j (Yang, 2007; Dos Reis and Yang, 2019) to estimate the divergence times among the eight species. To calibrate the divergence times, we established divergence time ranges for specific species pairs, namely *S. longa*-*N. nucifera* (~126-132 Mya), *C. chinensis*-*A. coerulea* (~64-79 Mya), and *P. somniferum*-*M. cordata* (~44-82 Mya). In order to ensure the convergence of the divergence time results, the mcmctree program was executed twice, and the percentage deviation between the results of the two runs was required to be less than 0.1%.

### Gene family analysis

We employed the Orthofinder software v2.5.4 (Emms and Kelly, 2019) to conduct cluster analysis and identify orthologous gene sets among *S. japonica, A. coerulea*, *C. chinensis*, *A. trifoliata*, *K. uniflora*, *P. somniferum*, *M. cordata*, *N. nucifera*, and *S. longa*. Subsequently, we utilized the CAFE software v4.2.1 (Han et al., 2013) with the identified orthologous gene sets and an evolutionary time tree to assess gene family expansions and contractions at various species and nodes. To ensure the quality of the input files, we initially filtered them using the python script cafetutorial_clade_and_size_filter.py, which can be found in the CAFE software package. Following the filtering step, we executed the CAFE software twice. The first run was performed to estimate the lambda parameter, while the second run utilized the estimated lambda value to calculate the expansions and contractions of gene families in the eight species. CAFE conducted statistical tests for each gene family at each node, and we retained only the results with a p-value less than 0.05 for further analysis. To annotate the gene families showing expansions or contractions, we utilized the Gene Ontology Resource (http://geneontology.org/) for Gene Ontology (GO) annotation and the GHOSTKOALA tool (https://www.kegg.jp/ghostkoala/) for Kyoto Encyclopedia of Genes and Genomes (KEGG) annotation.

### Substitution rate of synonymous and nonsynonymous sites

We aligned the nucleotide sequences of single-copy orthologous gene families from 9 species using Prank software v.170703 (Löytynoja, 2014). Subsequently, we used the Gblocks software v0.91b (Castresana, 2000) in codon alignment mode to filter out poorly conserved positions and remove information sites containing gaps. Next, the Codeml subroutine in PAML software v4.9j (Yang, 2007) was employed to calculate the ratio of non-synonymous substitutions (dN) to synonymous substitutions (dS) and detect selective pressure among the species. To measure selection on each protein, we utilized the free-ratio model (model=1), assuming an independent ω ratio for each branch. We randomly selected 150 genes from the orthologous single-copy gene set and repeated this process 10,000 times to compare selection pressure on different branches. Furthermore, we applied the branch-site model to analyze selection pressure, considering fixed and variable ω values. *S. longa* was designated as the foreground branch, and we used chi-square tests and false discovery rate (FDR) tests to identify genes under positive selection. The chi-square tests quantified the difference in the number of parameters between the models by calculating twice the difference in log-likelihood values and degrees of freedom. Genes were considered to have experienced common ancestral positive selection if the FDR value was less than 0.05, as determined by Bayesian empirical Bayes (BEB) analysis.

### Whole-genome duplication and synteny analysis

We employed the MCScanX toolkit (Wang et al., 2012) to analyze genome collinearity within and between five species, namely *S. longa*, *S. japonica, C. chinensis*, and *A. trifoliata* and *V. vinifera*. To conduct the analysis, we first performed whole-genome alignment using LAST v1282 (Kiełbasa et al., 2011). Collinear blocks were identified, and a minimum of 5 gene pairs was required for each block. By examining the relative positions and orientations of gene pairs within the collinear blocks, we determined the collinearity patterns in the genome and inferred the whole-genome duplication history.

To obtain paralogous gene families, we extracted genes from the collinear blocks identified through the MCScanX analysis. Each pair of protein sequences was then aligned using Muscle software v5.1 (Edgar, 2022). The resulting aligned amino acid matrix was converted to a nucleotide matrix using pal2nal software v14 (Suyama et al., 2006). Subsequently, gene cluster analysis based on CDS alignment was performed to identify the paralogous gene families. We utilized KaKs_Calculator software v3.0 (Zhang, 2022) to calculate the Ks values for the identified paralogous gene families. To identify significant peaks consistent with whole-genome duplication (WGD) events, we generated a Ks distribution curve using the ggplot2 R package.

### Metabolomic analysis

The freeze-dried samples of fruits, roots, leaves, and stems were crushed using a mixer mill (MM 400, Retsch) with a zirconia bead for 1.5 min at 30 Hz. A total of 50 mg of powder was dissolved in 1.2 ml of 70% methanol. All extracts were centrifuged at 12,000 rpm for 5 min, and the supernatants were filtered using a 0.22 μm PTFE filter. The filered supernatants were subjected to analysis using an UPLC-ESI-MS/MS system (UPLC, SHIMADZU Nexera X2, https://www.shimadzu.com.cn/; MS, Applied Biosystems 4500 Q TRAP, https://www.thermofisher.cn/cn/zh/home/brands/applied-biosystems.html). The analytical procedure was conducted using an Agilent SB-C18 UPLC column (1.8 µm, 2.1 mm x 100 mm). The mobile phase comprised Solvent A (pure water with 0.1% formic acid) and Solvent B (acetonitrile with 0.1% formic acid). The analysis initiated with a mobile phase of 95% Solvent A and 5% Solvent B. A linear gradient was applied over 9 minutes to shift to 5% Solvent A and 95% Solvent B, which was then maintained for 1 minute. The system was subsequently returned to 95% Solvent A and 5% Solvent B over 1.1 minutes and maintained for an additional 2.9 minutes. The flow rate was set at 0.35 mL/min, the column oven temperature was 40°C, and the injection volume was 4 μL. Detection was achieved using an ESI-triple quadrupole-linear ion trap mass spectrometer (QTRAP-MS).

The operation parameters for the ESI source in the mass spectrometric analysis were meticulously set to optimize detection. The source temperature was maintained at 550°C, and the ion spray voltage was adjusted to 5500 V in positive ion mode and -4500 V in negative ion mode. The ion source gases I and II were set at 50 and 60 psi, respectively, while the curtain gas was set at 25 psi. The collision-activated dissociation (CAD) was kept at a high setting. Instrument tuning and mass calibration were carried out using 10 and 100 μmol/L polypropylene glycol solutions for QQQ and LIT modes, respectively. For the quantitative analysis, QQQ scans were performed as Multiple Reaction Monitoring (MRM) experiments with the collision gas (nitrogen) set to a medium level. The declustering potential (DP) and collision energy (CE) for individual MRM transitions were optimized based on the specific metabolites expected during each period of the run, ensuring precise and targeted detection.

Metabolic profiling is carried out using the proprietary MWDB (Metware Database) from Wuhan Maiwei Metabolic Biotechnology Co., Ltd., complemented by secondary spectral data, to identify metabolites. Quantitative analysis is executed using triple quadrupole mass spectrometry, specifically employing MRM mode to ensure precise measurement of metabolites. Utilize Analyst 1.6.3 software to view the total ion chromatogram and multi-peak graphs for MRM metabolite detection. Principal component analysis (PCA) was performed using the prcomp function within the R software (www.r-project.org) to assess the overall variation in the dataset. To identify differences between the samples, hierarchical cluster analysis (HCA) and orthogonal partial least squares discriminant analysis (OPLS-DA) were conducted using R (MetaboAnalystR package). Metabolites with a variable importance in projection (VIP) score of 1 or higher and a fold change of 2 or higher, or a fold change of 0.5 or lower were considered as differentially regulated metabolites. Metabolites identified in the study were annotated using the KEGG Compound database (https://www.kegg.jp/kegg/compound/). The metabolome annotation files for *S. longa* have been uploaded to Figshare and can be accessed at https://doi.org/10.6084/m9.figshare.26125690. Subsequently, these annotated metabolites were mapped onto biological pathways using the KEGG Pathway database (https://www.kegg.jp/kegg/pathway.html). Pathways that contained significantly regulated metabolites were then subjected to Metabolite Set Enrichment Analysis (MSEA). The significance of these pathways was assessed using the hypergeometric test, with the resulting p-values indicating the level of significance.

### Transcriptome analysis

Plant material, including fruits, roots, leaves, and stems from *S. longa*, was collected and immediately flash-frozen in liquid nitrogen. The frozen samples were stored at -80°C until further processing. RNA extraction was conducted using the RNAprep Pure Plant Kit (TIANGEN, China) following the manufacturer’s protocol. Following successful RNA extraction, cDNA libraries were synthesized using the extracted RNA from each sample type. Paired-end sequencing was performed on the Illumina NovaSeq 6000 platform, generating 150 bp reads for each library. To quantify gene expression levels, the raw sequencing data were processed and aligned to the *S. longa* reference genome. The number of clean reads for each contig was calculated, and subsequent normalization was performed to obtain reads per kilobase per million reads (RPKM) values. Differential expression analysis between groups was performed using DESeq2 v1.22.1 and edgeR v3.24.3. The resulting P-values were adjusted using the false discovery rate (FDR) method. To determine significant differential expression, the threshold was set based on the corrected P-value and |log2foldchange|. Typically, differentially expressed genes are defined as having an absolute log2Fold Change greater than or equal to 1 and an FDR lower than 0.05. Enrichment analysis was performed using the hypergeometric test.

## Data Availability

The sequencing reads and genome assembly of *S. longa* have been deposited in the NCBI BioProject database under accession number PRJNA1088790.
