# Supplementary material for: Genome assembly of Stephania longa provides insight into cepharanthine biosynthesis
Source: Front Plant Sci. 2024 Sep 5;15:1414636. doi: 10.3389/fpls.2024.1414636 (PMC11410628; doi:10.3389/fpls.2024.1414636)
Supplement: Supplementary file 1 [file DataSheet1.pdf]

Supplementary Information for

Genome assembly of *Stephania longa* provides insight into cepharanthine biosynthesis

This file includes: Supplementary Figure S1-S11 and Table S1-S24

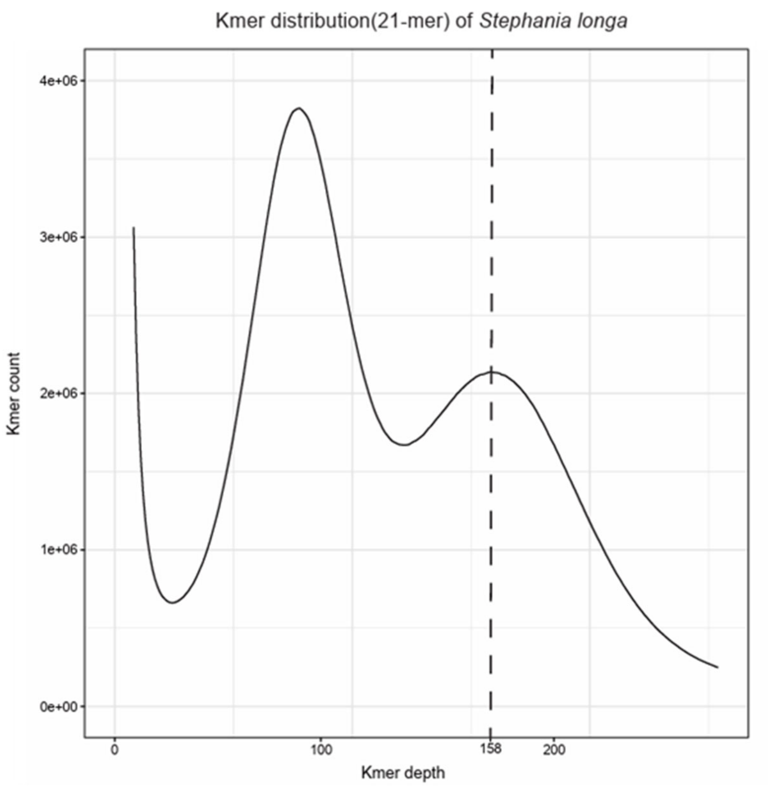

Supplementary Figure S1 The K-mer (k=21) distribution of the *Stephania longa* genome. The genome size is inferred by dividing the total number of K-mers by the depth of K-mers. Based on these calculations, the heterozygosity rate and estimated genome size for *S. longa* is 0.015 and 624.08 Mb, respectively.

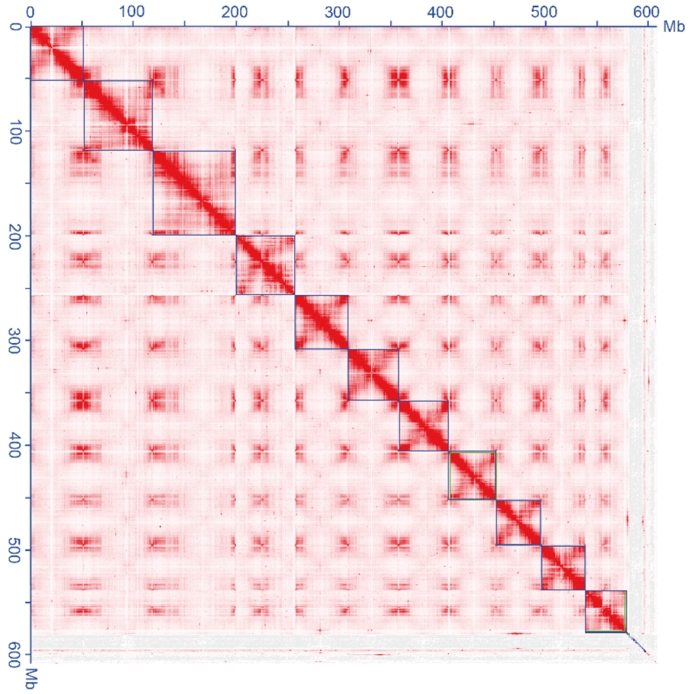

Supplementary Figure S2 The interactions of 11 *S. longa* chromosomes obtained by Hi-C map.

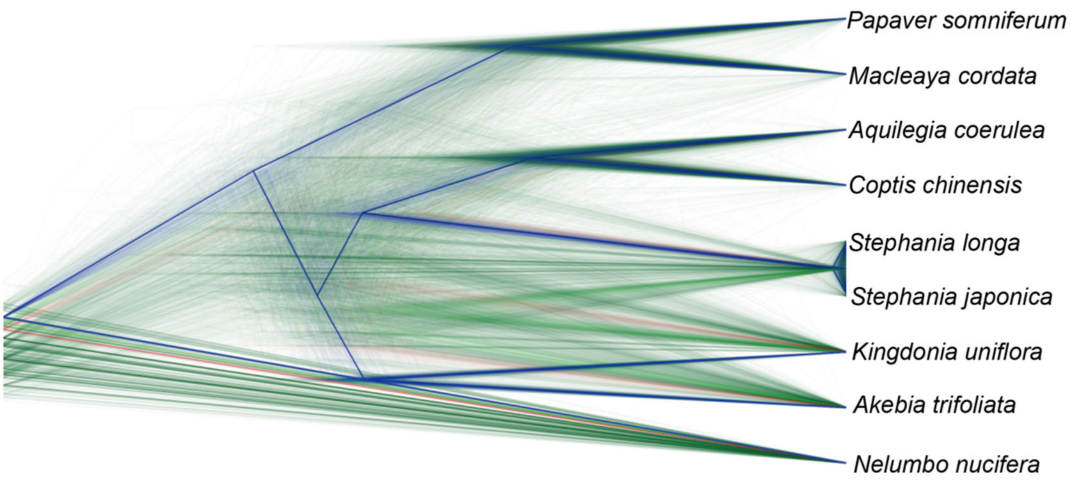

Supplementary Figure S3 The phylogenetic tree illustrating the relationship among orthologous genes, highlighting a notable level of incomplete lineage sorting during their early divergence stages.

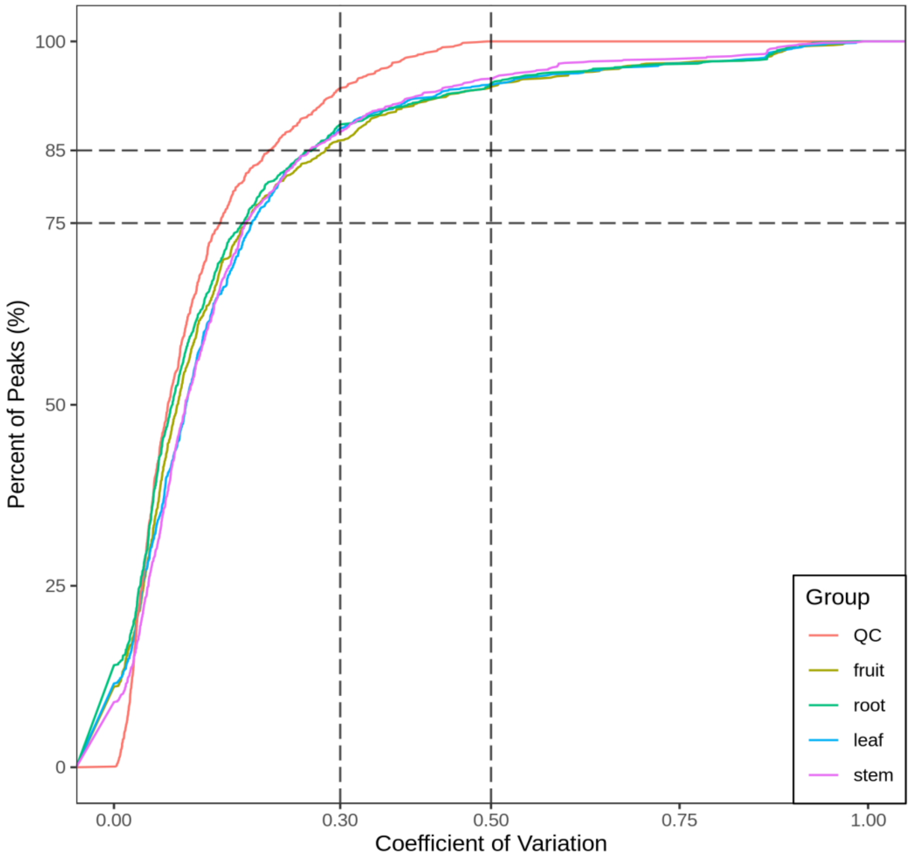

Supplementary Figure S4 The coefficient of variation (CV) for each organ of *Stephania longa*.

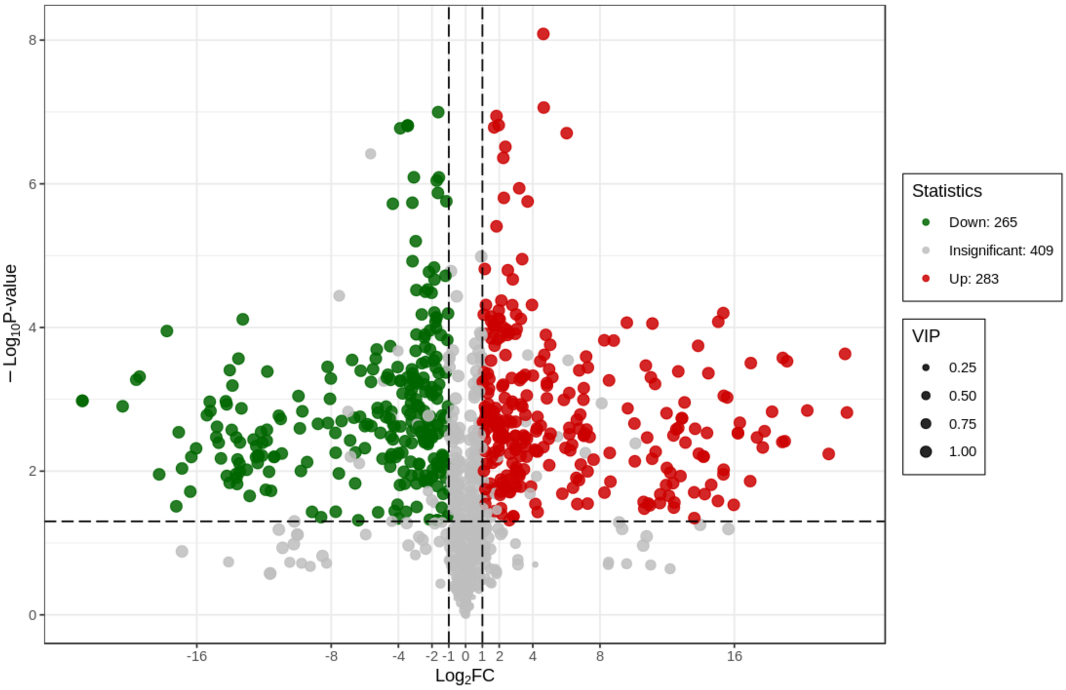

Supplementary Figure S5 The figure depicts a volcano plot illustrating the differential metabolites between fruit and leaf tissues. The plot displays the fold change of metabolite abundance on the x-axis and the statistical significance on the y-axis.

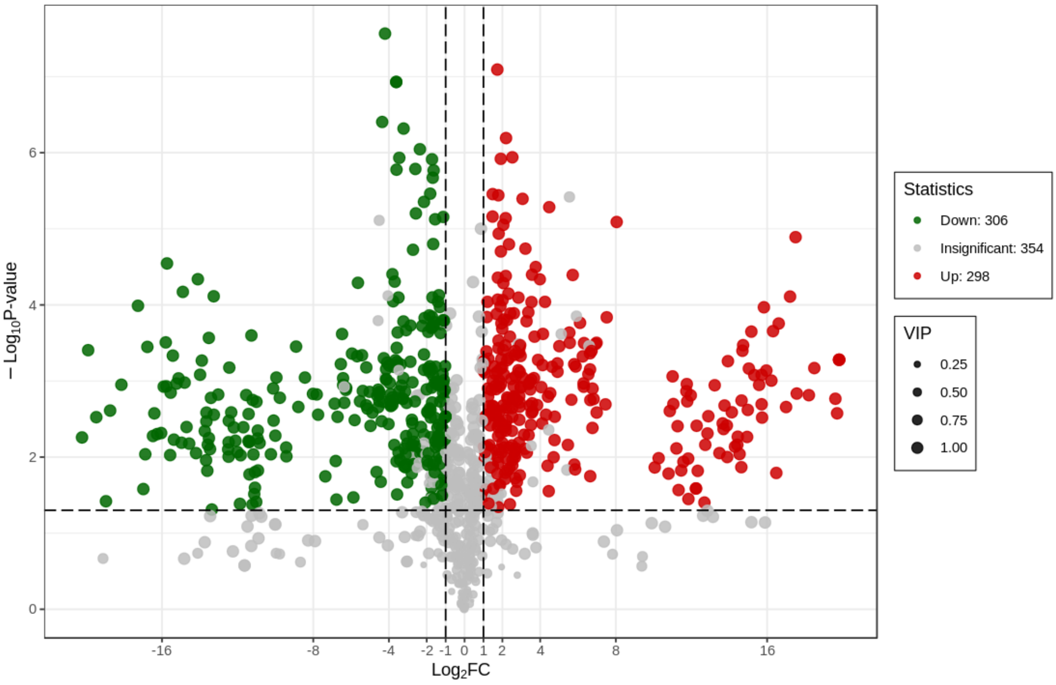

Supplementary Figure S6 The figure depicts a volcano plot illustrating the differential metabolites

between fruit and root tissues. The plot displays the fold change of metabolite abundance on the x-axis and the statistical significance on the y-axis.

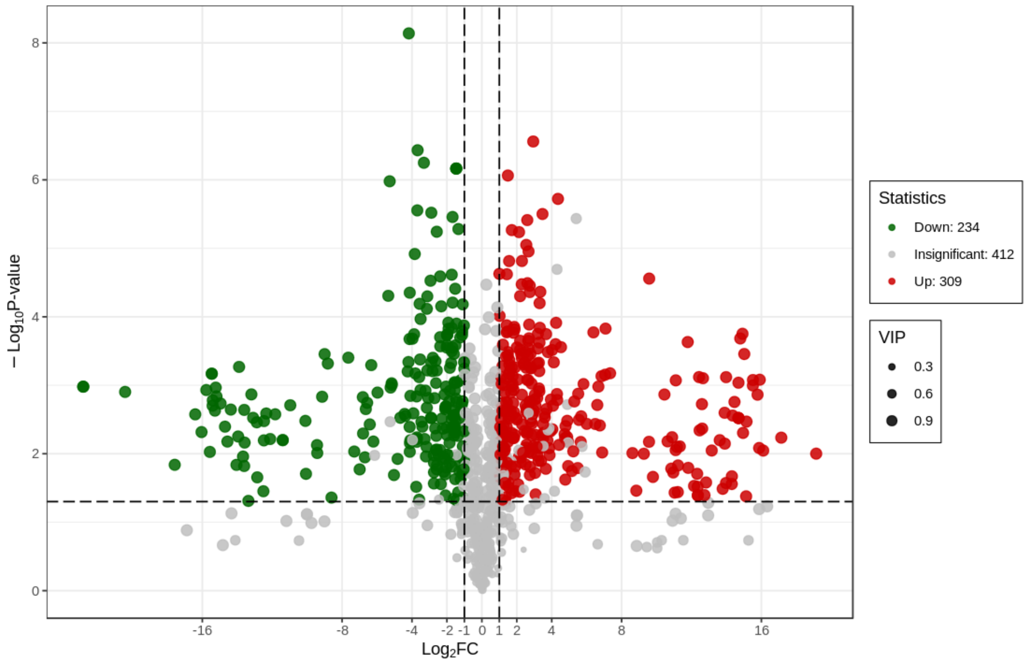

Supplementary Figure S7 The figure depicts a volcano plot illustrating the differential metabolites between fruit and stem tissues. The plot displays the fold change of metabolite abundance on the x-axis and the statistical significance on the y-axis.

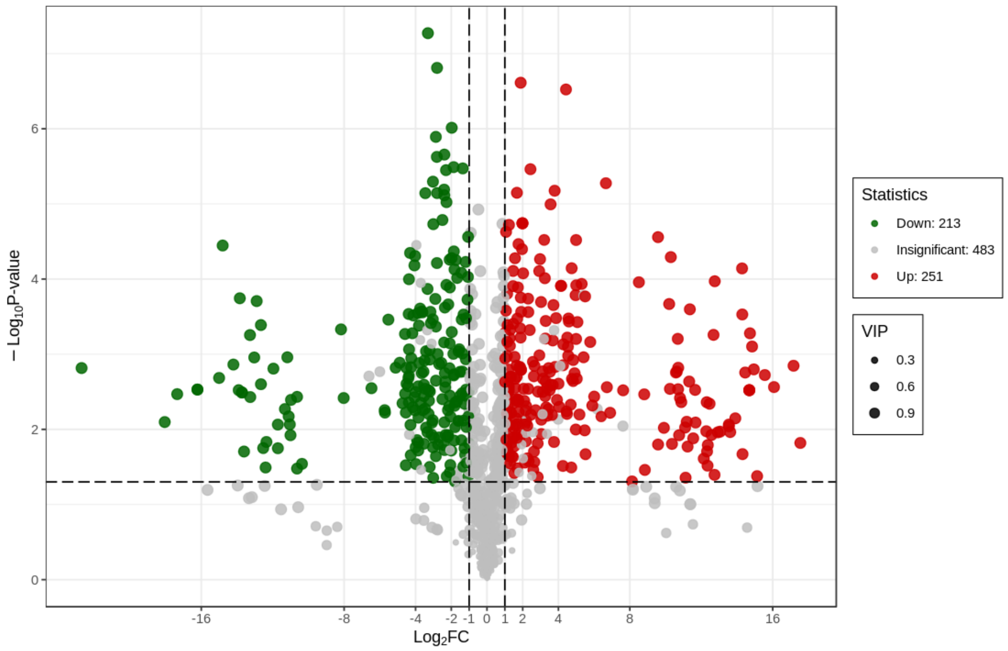

Supplementary Figure S8 The figure depicts a volcano plot illustrating the differential metabolites between leaf and stem tissues. The plot displays the fold change of metabolite abundance on the x-

axis and the statistical significance on the y-axis.

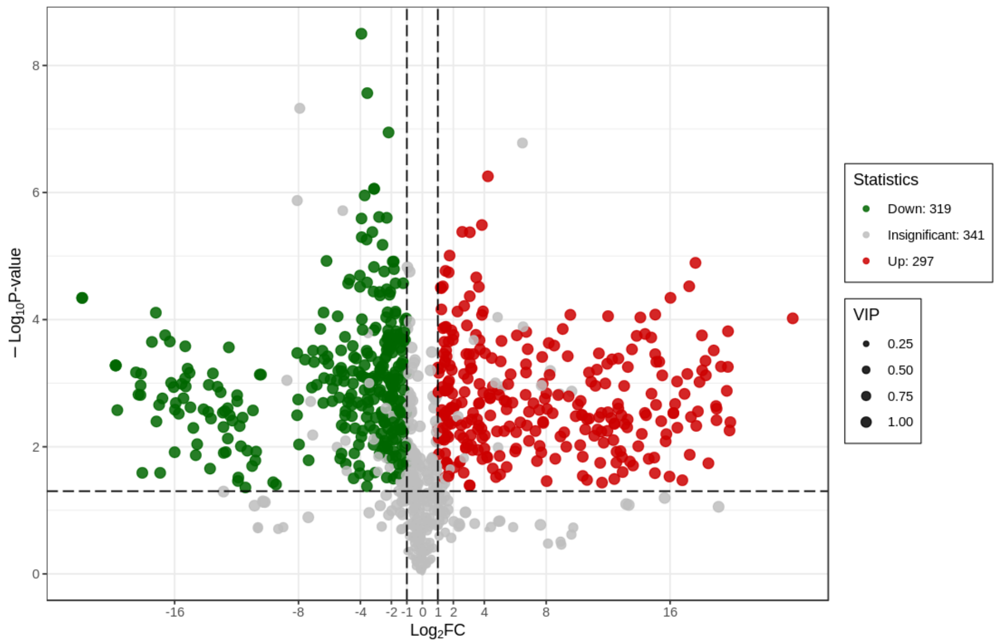

Supplementary Figure S9 The figure depicts a volcano plot illustrating the differential metabolites between root and leaf tissues. The plot displays the fold change of metabolite abundance on the x-axis and the statistical significance on the y-axis.

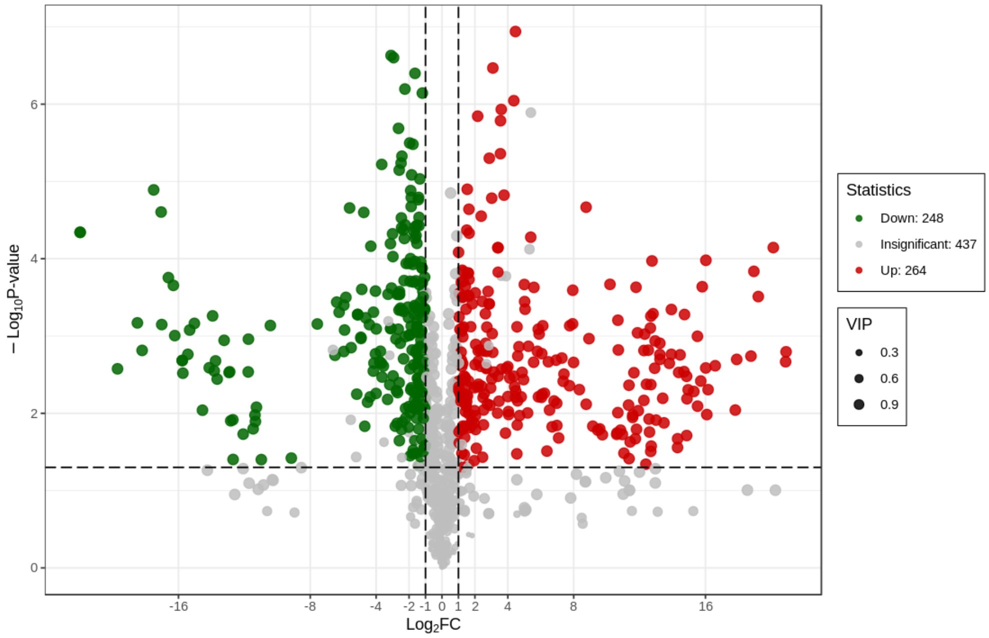

Root vs stem

Supplementary Figure S10 The figure depicts a volcano plot illustrating the differential metabolites between root and stem tissues. The plot displays the fold change of metabolite abundance on the x-axis and the statistical significance on the y-axis.

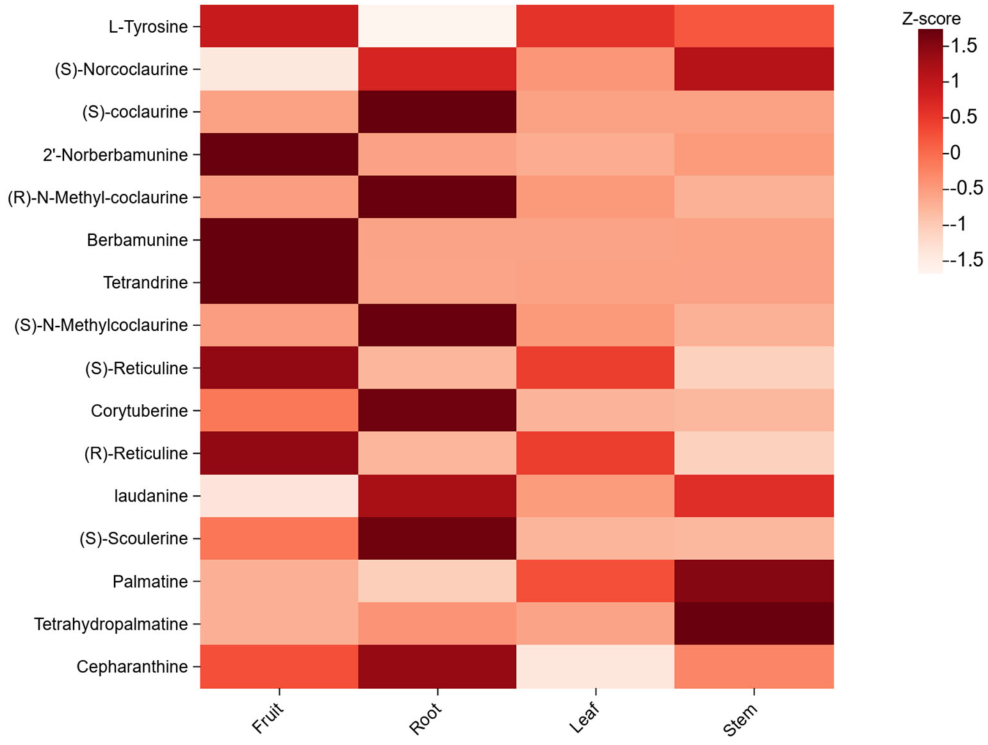

Supplementary Figure S11 Heatmap depicting the expression levels of metabolites closely associated with cepharanthine across various tissues. The heatmap illustrates the variations in expression levels of these metabolites.

Supplementary Table S1. Statistics of the cleaned genomic reads.

Type	Sample	Platform	Bases (bp)	Reads Count	Max length (bp)	Mean length (bp)
Illumina	Leaf	HiSeq2000	122,254,467,681	817,631,248	150	150
HiFi	Leaf	PacBio Sequel II	22,946,830,961	1,369,750	49,911	16,752
Hi-C	Leaf	DNBSEQ-T7	64,700,334,900	431,335,566	150	150
RNA	Fruit01	DNBSEQ-T7	7,220,704,653	47,863,204	150	150
	Fruit02	DNBSEQ-T7	7,843,352,265	52,004,912	150	150
	Leaf01	DNBSEQ-T7	6,167,773,783	40,886,354	150	150
	Leaf02	DNBSEQ-T7	5,795,431,702	38,438,156	150	150
	Root01	DNBSEQ-T7	6,002,855,757	39,794,392	150	150
	Root02	DNBSEQ-T7	6,280,072,561	41,638,606	150	150
	Stem01	DNBSEQ-T7	6,867,657,800	45,533,282	150	150
	Stem02	DNBSEQ-T7	6,769,986,713	44,893,510	150	150

Supplementary Table S2. Statistics of the genome assembly.

Term	Contig assembly		Hi-C assembly	
	Size (bp)	Number	Size (bp)	Number
N90	4,149,436	31	38,391,000	11

N80	9,837,489	24	42,918,000	10
N70	13,026,994	19	45,206,836	8
N60	16,754,862	15	47,469,000	7
N50	21,223,701	11	49,162,663	6
Max length (bp)	48,651,511	-	80,332,011	-
Total size (bp)	614768797	-	614840797	-
Total number	-	541	-	397
Percent assembled (%)	99.9		100	

Supplementary Table S3. Statistics of the draft chromosome-level genome assembly.

Chromosome ID	Length (bp)
chr1	51,576,500
chr2	49,162,663
chr3	43,119,043
chr4	80,332,011
chr5	66,810,489
chr6	42,918,000
chr7	47,469,000
chr8	51,288,500
chr9	45,206,836
chr10	38,391,000
chr11	56,508,221
Total length (bp)	572,782,263
Total length/Genome size (%)	93.16

Supplementary Table S4. Statistics of the genome assembly on the presence of conserved BUSCO orthologs.

Library	embryophyta_odb10
Complete BUSCOs (C)	1,581
Complete and single-copy BUSCOs (S)	1,547
Complete and duplicated BUSCOs (D)	34
Fragmented BUSCOs (F)	18
Missing BUSCOs (M)	15
Total BUSCO groups searched	1,614
Summarize	97.9%

Supplementary Table S5. Summary of repetitive sequences in *Stephania longa*.

	Repbase TEs		TE proteins		De novo		Combined TEs	
Type	Length (bp)	Percent in genome (%)	Length (bp)	Percent in genome (%)	Length (bp)	Percent in genome (%)	Length (bp)	Percent in genome (%)
DNA	1,818,281	0.30	44,969	0.01	35,895,462	5.84	36,482,156	5.93
LINE	433,089	0.07	32,421	0.01	16,484,787	2.68	16,658,671	2.71

SINE	0	0.00	0	0	866,517	14.00	866,517	0.14
LTR	40,723,294	6.62	69,618,909	11.32	180,334,989	29.33	192,664,850	31.34
Other	173,972	0.03	318	0.00	4,190,936	0.68	4,345,673	0.71
Unknown	24,422	0.00	0	0.00	182,685,786	29.71	182,708,918	29.72
Total	44,623,160	7.26	69,786,562	11.35	410,240,481	66.72	418,065,900	68.00

Supplementary Table S6. Statistics of the functional annotation of protein-coding genes.

Term	Number	Percent (%)
InterPro	22,903	65.53
GO	14,648	41.91
KEGG	17,206	49.23
Swissprot	22,272	63.72
TrEMBL	27,581	78.91
COG	11,004	31.48
NR	28,043	80.24
Annotated genes	28,629	81.91
Unannotated genes	6,322	18.09
Total genes	34,951	-

Supplementary Table S7. Summary of gene density across different chromosomes.

Chromosome ID	Length (bp)	Gene count	Gene density (per Mb)
chr1	51,576,500	2,985	57.88
chr2	49,162,663	2,742	55.77
chr3	43,119,043	2,489	57.72
chr4	80,332,011	4,700	58.51
chr5	66,810,489	3,748	56.10
chr6	42,918,000	2,271	52.91
chr7	47,469,000	2,793	58.84
chr8	51,288,500	2,775	54.11
chr9	45,206,836	2,327	51.47
chr10	38,391,000	2,091	54.47
chr11	56,508,221	2,991	52.93

Supplementary Table S8. Statistics on annotated genes from the *Stephania longa* and other species used in this study.

Species name	Genome size(bp)	Total number	Average gene length(bp)	Average exon number	Average exon length	Average intron length

					(bp)	(bp)
Stephania longa	614,840,797	34,951	4,239.51	4.23	322.08	890.73
Stephania japonica	643,419,807	26,695	4,881.85	4.84	220.03	994.35
Papaver somniferum	2,710,122,729	62,933	2,520.66	4.57	268.36	362.48
Macleaya cordata	344,987,380	21,911	3,723.21	5.30	236.40	574.13
Coptis chinensis	935,659,780	42,450	3,529.36	4.21	222.93	806.93
Aquilegia coerulea	306,516,179	43,550	3,436.84	5.53	223.66	485.65
Akebia trifoliata	652,732,637	21,923	6,573.30	4.97	248.10	1344.02
Kingdonia uniflora	1,004,558,319	42,833	3,639.75	4.14	228.74	856.56
Nelumbo nucifera	804,647,751	23,966	10,579.10	5.53	243.17	2036.84

Supplementary Table S9. Statistics on the tissue-specific gene expression quantity.

TPM	Fruit1	Fruit2	Leaf1	Leaf2	Root1	Root2	Stem1	Stem2	ALL
TPM>0	21,052	21,247	20,404	20,329	20,781	20,904	21,136	21,140	24,791
TPM>1	16,059	15,985	16,246	16,156	17,037	17,023	16,891	16,858	17,004
TPM>5	11,728	11,726	12,564	12,558	13,422	13,407	13,070	13,029	9,612
TPM>10	9,319	9,288	10,594	10,551	11,438	11,422	10,981	10,877	7,387

Supplementary Table S10. Gene trees with frequently occurring topologies (more than 10 times).

Topology	Count
(((((S.japonica,S.longa),(C.chinensis,A.coerulea)),(K.uniflora,A.trifoliata)),(M.cordata,P.somniferum)),N.nucifera)	177
((((((S.japonica,S.longa),(C.chinensis,A.coerulea)),K.uniflora),A.trifoliata),(M.cordata,P.somniferum)),N.nucifera)	67
((((((S.japonica,S.longa),K.uniflora),(C.chinensis,A.coerulea)),A.trifoliata),(M.cordata,P.somniferum)),N.nucifera)	62
(((((S.japonica,S.longa),A.trifoliata),((C.chinensis,A.coerulea),K.uniflora)),(M.cordata,P.somniferum)),N.nucifera)	54
(((((M.cordata,P.somniferum),(C.chinensis,A.coerulea)),(K.uniflora,A.trifoliata)),(S.japonica,S.longa)),N.nucifera)	52
((((((C.chinensis,A.coerulea),K.uniflora),(M.cordata,P.somniferum)),A.trifoliata),(S.japonica,S.longa)),N.nucifera)	51
((((((S.japonica,S.longa),(C.chinensis,A.coerulea)),A.trifoliata),K.uniflora),(M.cordata,P.somniferum)),N.nucifera)	50
((((((C.chinensis,A.coerulea),K.uniflora),A.trifoliata),(M.cordata,P.somniferum)),(S.japonica,S.longa)),N.nucifera)	43
(((((K.uniflora,A.trifoliata),(C.chinensis,A.coerulea)),(M.cordata,P.somniferum)),(S.japonica,S.longa)),N.nucifera)	41

(((((M.cordata,P.somniferum),(K.uniflora,A.trifoliata)),(C.chinensis,A.coerulea)),(S.japonica,S.longa)),N.nucifera)	39
((((S.japonica,S.longa),(C.chinensis,A.coerulea)),((M.cordata,P.somniferum),(K.uniflora,A.trifoliata))),N.nucifera)	38
((((((S.japonica,S.longa),K.uniflora),A.trifoliata),(C.chinensis,A.coerulea)),(M.cordata,P.somniferum)),N.nucifera)	35
(((((S.japonica,S.longa),(C.chinensis,A.coerulea)),(M.cordata,P.somniferum)),(K.uniflora,A.trifoliata)),N.nucifera)	33
(((((M.cordata,P.somniferum),A.trifoliata),((C.chinensis,A.coerulea),K.uniflora)),(S.japonica,S.longa)),N.nucifera)	31
((((((C.chinensis,A.coerulea),K.uniflora),A.trifoliata),(S.japonica,S.longa)),(M.cordata,P.somniferum)),N.nucifera)	31
(((((K.uniflora,A.trifoliata),(C.chinensis,A.coerulea)),(S.japonica,S.longa)),(M.cordata,P.somniferum)),N.nucifera)	30
((((((M.cordata,P.somniferum),(C.chinensis,A.coerulea)),K.uniflora),A.trifoliata),(S.japonica,S.longa)),N.nucifera)	28
(((((S.japonica,S.longa),(K.uniflora,A.trifoliata)),(C.chinensis,A.coerulea)),(M.cordata,P.somniferum)),N.nucifera)	27
(((((S.japonica,S.longa),K.uniflora),((C.chinensis,A.coerulea),A.trifoliata)),(M.cordata,P.somniferum)),N.nucifera)	27
((((((C.chinensis,A.coerulea),K.uniflora),(S.japonica,S.longa)),A.trifoliata),(M.cordata,P.somniferum)),N.nucifera)	27
((((((M.cordata,P.somniferum),K.uniflora),(C.chinensis,A.coerulea)),A.trifoliata),(S.japonica,S.longa)),N.nucifera)	27
(((((S.japonica,S.longa),(M.cordata,P.somniferum)),(K.uniflora,A.trifoliata)),(C.chinensis,A.coerulea)),N.nucifera)	26
((((((M.cordata,P.somniferum),(C.chinensis,A.coerulea)),A.trifoliata),K.uniflora),(S.japonica,S.longa)),N.nucifera)	25
(((((M.cordata,P.somniferum),K.uniflora),((C.chinensis,A.coerulea),A.trifoliata)),(S.japonica,S.longa)),N.nucifera)	22
(((((S.japonica,S.longa),(C.chinensis,A.coerulea)),((M.cordata,P.somniferum),K.uniflora)),A.trifoliata),N.nucifera)	20
(((((M.cordata,P.somniferum),K.uniflora),A.trifoliata),((S.japonica,S.longa),(C.chinensis,A.coerulea))),N.nucifera)	20
(((((S.japonica,S.longa),(C.chinensis,A.coerulea)),((M.cordata,P.somniferum),A.trifoliata)),K.uniflora),N.nucifera)	20
((((((S.japonica,S.longa),(C.chinensis,A.coerulea)),A.trifoliata),(M.cordata,P.somniferum)),K.uniflora),N.nucifera)	19
((((((S.japonica,S.longa),(C.chinensis,A.coerulea)),K.uniflora),(M.cordata,P.somniferum)),A.trifoliata),N.nucifera)	19
((((((M.cordata,P.somniferum),K.uniflora),A.trifoliata),(C.chinensis,A.coerulea)),(S.japonica,S.longa)),N.nucifera)	19
(((((S.japonica,S.longa),K.uniflora),((M.cordata,P.somniferum),(C.chinensis,A.coerulea))),A.trifoliata),N.nucifera)	19

(((((S.japonica,S.longa),(C.chinensis,A.coerulea)),K.uniflora),((M.cordata,P.somniferum),A.trifoliata)),N.nucifera)	18
((((((S.japonica,S.longa),A.trifoliata),K.uniflora),(C.chinensis,A.coerulea)),(M.cordata,P.somniferum)),N.nucifera)	18
(((((S.japonica,S.longa),K.uniflora),A.trifoliata),((M.cordata,P.somniferum),(C.chinensis,A.coerulea))),N.nucifera)	18
((((((C.chinensis,A.coerulea),A.trifoliata),K.uniflora),(M.cordata,P.somniferum)),(S.japonica,S.longa)),N.nucifera)	17
(((((C.chinensis,A.coerulea),K.uniflora),(M.cordata,P.somniferum)),((S.japonica,S.longa),A.trifoliata)),N.nucifera)	17
(((((S.japonica,S.longa),(C.chinensis,A.coerulea)),A.trifoliata),((M.cordata,P.somniferum),K.uniflora)),N.nucifera)	17
((((((S.japonica,S.longa),K.uniflora),(C.chinensis,A.coerulea)),(M.cordata,P.somniferum)),A.trifoliata),N.nucifera)	16
((((((C.chinensis,A.coerulea),K.uniflora),(S.japonica,S.longa)),(M.cordata,P.somniferum)),A.trifoliata),N.nucifera)	16
(((((S.japonica,S.longa),K.uniflora),((M.cordata,P.somniferum),A.trifoliata)),(C.chinensis,A.coerulea)),N.nucifera)	16
((((S.japonica,S.longa),(M.cordata,P.somniferum)),((K.uniflora,A.trifoliata),(C.chinensis,A.coerulea))),N.nucifera)	15
(((((M.cordata,P.somniferum),(C.chinensis,A.coerulea)),A.trifoliata),((S.japonica,S.longa),K.uniflora)),N.nucifera)	15
((((((C.chinensis,A.coerulea),A.trifoliata),(M.cordata,P.somniferum)),K.uniflora),(S.japonica,S.longa)),N.nucifera)	14
(((((S.japonica,S.longa),(M.cordata,P.somniferum)),((C.chinensis,A.coerulea),K.uniflora)),A.trifoliata),N.nucifera)	14
((((((S.japonica,S.longa),(C.chinensis,A.coerulea)),(M.cordata,P.somniferum)),K.uniflora),A.trifoliata),N.nucifera)	14
((((((S.japonica,S.longa),(C.chinensis,A.coerulea)),(M.cordata,P.somniferum)),A.trifoliata),K.uniflora),N.nucifera)	14
((((((S.japonica,S.longa),A.trifoliata),(C.chinensis,A.coerulea)),K.uniflora),(M.cordata,P.somniferum)),N.nucifera)	14
((((((M.cordata,P.somniferum),A.trifoliata),K.uniflora),(C.chinensis,A.coerulea)),(S.japonica,S.longa)),N.nucifera)	14
(((((S.japonica,S.longa),(M.cordata,P.somniferum)),(C.chinensis,A.coerulea)),(K.uniflora,A.trifoliata)),N.nucifera)	13
((((((S.japonica,S.longa),K.uniflora),A.trifoliata),(M.cordata,P.somniferum)),(C.chinensis,A.coerulea)),N.nucifera)	13
(((((S.japonica,S.longa),K.uniflora),(C.chinensis,A.coerulea)),((M.cordata,P.somniferum),A.trifoliata)),N.nucifera)	13
(((((M.cordata,P.somniferum),A.trifoliata),K.uniflora),((S.japonica,S.longa),(C.chinensis,A.coerulea))),N.nucifera)	12
(((((S.japonica,S.longa),A.trifoliata),K.uniflora),((M.cordata,P.somniferum),(C.chinensis,A.coerulea))),N.nucifera)	12

((((((C.chinensis,A.coerulea),A.trifoliata),K.uniflora),(S.japonica,S.longa)),(M.cordata,P.somniferum)),N.nucifera)	11
((((((C.chinensis,A.coerulea),K.uniflora),(M.cordata,P.somniferum)),(S.japonica,S.longa)),A.trifoliata),N.nucifera)	11
(((((C.chinensis,A.coerulea),K.uniflora),A.trifoliata),((S.japonica,S.longa),(M.cordata,P.somniferum))),N.nucifera)	11
(((((S.japonica,S.longa),A.trifoliata),(C.chinensis,A.coerulea)),((M.cordata,P.somniferum),K.uniflora)),N.nucifera)	10
((((((S.japonica,S.longa),(M.cordata,P.somniferum)),(C.chinensis,A.coerulea)),K.uniflora),A.trifoliata),N.nucifera)	10
(((((M.cordata,P.somniferum),(K.uniflora,A.trifoliata)),(S.japonica,S.longa)),(C.chinensis,A.coerulea)),N.nucifera)	10
(((((S.japonica,S.longa),(K.uniflora,A.trifoliata)),(M.cordata,P.somniferum)),(C.chinensis,A.coerulea)),N.nucifera)	10

Supplementary Table S11. Statistics of the calculation of mutation rate from the *Stephania longa* and other species.

**Species name**	**Mutation rate (per year)**
Stephania longa	7.14×10^{-9}
Stephania japonica	1.21×10^{-8}
Papaver somniferum	7.06×10^{-9}
Macleaya cordata	2.07×10^{-9}
Coptis chinensis	6.87×10^{-9}
Aquilegia coerulea	7.04×10^{-9}
Akebia trifoliata	3.61×10^{-9}
Kingdonia uniflora	6.97×10^{-9}
Nelumbo nucifera	1.78×10^{-9}

Supplementary Table S12. Statistics of positive selection genes in the genus *Stephania*

Stephania longa gene id	*Stephania japonica* gene id	Common name
slo.H102416	Sjap.Chr4G00116410.1	*GLDP1*
slo.H119789	Sjap.Chr8G00205290.1	*ARGAH2*
slo.H114001	Sjap.Chr2G00048710.1	*AT1G01770*
slo.H117166	Sjap.Chr10G00245420.1	*CIB5*
slo.H121536	Sjap.Chr8G00191250.1	*AT1G80360*
slo.H120969	Sjap.Chr8G00196480.1	*TRZ4*
slo.H108444	Sjap.Chr1G00037580.1	*AT4G19900*
slo.H115387	Sjap.Chr2G00059890.1	*sks3*
slo.H109688	Sjap.Chr1G00027200.1	*CER7*
slo.H107866	Sjap.Chr7G00186690.1	*AT1G34150*

slo.H126185	Sjap.Chr9G00226080.1	*AT5G63680*
slo.H112850	Sjap.Chr1G00000760.1	*AT4G27390*
slo.H124176	Sjap.Chr5G00123890.1	*FACE2*
slo.H126596	Sjap.Chr9G00229470.1	*RCA*
slo.H126042	Sjap.Chr9G00225020.1	*NAC038*
slo.H112634	Sjap.Chr1G00002810.1	*SMG7*
slo.H108370	Sjap.Chr1G00038300.1	*SRT2*
slo.H113652	Sjap.Chr2G00046240.1	*MED31*
slo.H121161	Sjap.Chr8G00194720.1	*AT4G29590*
slo.H129846	Sjap.Chr3G00075990.1	*AT5G41120*
slo.H118443	Sjap.Chr10G00235820.1	*AT1G17230*
slo.H107724	Sjap.Chr7G00185570.1	*CDKB1;2*
slo.H112192	Sjap.Chr1G00006640.1	*AT5G45660*
slo.H116973	Sjap.Chr10G00247210.1	*AT5G16610*
slo.H128354	Sjap.Chr11G00264180.1	*UBP15*
slo.H123897	Sjap.Chr5G00126210.1	*AT3G58470*
slo.H102991	Sjap.Chr6G00167510.1	*ACS*
slo.H103250	Sjap.Chr6G00165180.1	*FRS3*
slo.H110528	Sjap.Chr1G00021160.1	*OVA2*
slo.H104069	Sjap.Chr6G00157970.1	*AT5G39865*
slo.H118700	Sjap.Chr10G00233660.1	*AT3G62310*
slo.H126358	Sjap.Chr9G00227520.1	*AT2G23093*
slo.H120980	Sjap.Chr8G00196410.1	*AT4G32790*
slo.H116114	Sjap.Chr2G00066330.1	*AT1G03210*
slo.H121008	Sjap.Chr8G00196180.1	*GL2*
slo.H102322	Sjap.Chr4G00115660.1	*AT1G73020*
slo.H122199	Sjap.Chr5G00140310.1	*AT3G09080*
slo.H103763	Sjap.Chr6G00160540.1	*AT3G26480*
slo.H123635	Sjap.Chr5G00128640.1	*AT1G63080*
slo.H128913	Sjap.Chr11G00260990.1	*APG7*
slo.H113043	Sjap.Chr2G00040720.1	*AT4G19130*
slo.H121562	Sjap.Chr8G00191050.1	*AT5G07770*
slo.H116428	Sjap.Chr2G00069070.1	*PUM6*
slo.H118419	Sjap.Chr10G00236190.1	*PDF1B*
slo.H127751	Sjap.Chr11G00253050.1	*AT5G54880*
slo.H116272	Sjap.Chr2G00067800.1	*ERF11*
slo.H114290	Sjap.Chr2G00051020.1	*GDAP1*
slo.H110876	Sjap.Chr1G00018430.1	*AT2G29290*
slo.H127022	Sjap.Chr11G00258990.1	*MBR1*
slo.H127115	Sjap.Chr11G00258220.1	*AT5G27410*
slo.H118137	Sjap.Chr10G00238350.1	*AT2G02590*
slo.H116967	Sjap.Chr10G00247280.1	*AT3G17920*
slo.H112008	Sjap.Chr1G00008270.1	*RLT2*
slo.H107826	Sjap.Chr7G00186370.1	*EME1A*

slo.H116655	Sjap.Chr2G00070940.1	*AT3G62010*
slo.H119342	Sjap.Chr8G00209170.1	*AT1G18410*
slo.H116394	Sjap.Chr2G00068830.1	*AT5G55070*
slo.H101657	Sjap.Chr4G00110700.1	*AT4G32060*
slo.H105502	Sjap.Chr6G00146290.1	*ARF2*
slo.H111349	Sjap.Chr1G00013880.1	*AT3G02760*
slo.H120825	Sjap.Chr8G00197750.1	*CID4*
slo.H105391	Sjap.Chr6G00147110.1	*AT2G33680*
slo.H102446	Sjap.Chr4G00116650.1	*emb1688*
slo.H121679	Sjap.Chr8G00190030.1	*AT1G51610*
slo.H128214	Sjap.Chr11G00265500.1	*ABA3*
slo.H116929	Sjap.Chr10G00247780.1	*AT1G14810*
slo.H105544	Sjap.Chr6G00145890.1	*AT1G09130*
slo.H124208	Sjap.Chr5G00123610.1	*ROC4*
slo.H107883	Sjap.Chr7G00186850.1	*AT3G07700*
slo.H101222	Sjap.Chr4G00107120.1	*UBP15*
slo.H103719	Sjap.Chr6G00160830.1	*DWA3*
slo.H116961	Sjap.Chr10G00247330.1	*AT5G06660*
slo.H124514	Sjap.Chr9G00212600.1	*FPA*
slo.H126059	Sjap.Chr9G00225160.1	*AT5G16350*
slo.H114985	Sjap.Chr2G00056370.1	*AT4G24090*
slo.H116374	Sjap.Chr2G00068670.1	*AT4G02425*
slo.H103911	Sjap.Chr6G00159170.1	*AT4G27480*
slo.H109101	Sjap.Chr1G00031990.1	*AT4G26950*
slo.H116058	Sjap.Chr2G00065820.1	*AT1G03150*
slo.H101267	Sjap.Chr4G00107480.1	*AT5G14850*
slo.H112963	Sjap.Chr2G00040010.1	*AT5G02860*
slo.H105396	Sjap.Chr6G00147060.1	*GR-RBP2*
slo.H111474	Sjap.Chr1G00012870.1	*PDAT*
slo.H118677	Sjap.Chr10G00233860.1	*AT2G44500*
slo.H105610	Sjap.Chr6G00145240.1	*STT3A*
slo.H111966	Sjap.Chr1G00008680.1	*TPR9*
slo.H129301	Sjap.Chr3G00080730.1	*AT3G22845*
slo.H119120	Sjap.Chr8G00211090.1	*TUB8*
slo.H113995	Sjap.Chr2G00048650.1	*KEA2*
slo.H115581	Sjap.Chr2G00061500.1	*AT5G38890*
slo.H103330	Sjap.Chr6G00164440.1	*POLA2*
slo.H125109	Sjap.Chr9G00217400.1	*AT1G21580*
slo.H103677	Sjap.Chr6G00161300.1	*AT5G26680*
slo.H108609	Sjap.Chr1G00036400.1	*QRT3*
slo.H124876	Sjap.Chr9G00215650.1	*AT1G18340*
slo.H103684	Sjap.Chr6G00161240.1	*PEX14*
slo.H103374	Sjap.Chr6G00164030.1	*EMB2423*
slo.H122251	Sjap.Chr5G00139850.1	*AT1G05670*

slo.H118581	Sjap.Chr10G00234650.1	*AT5G63200*
slo.H101027	Sjap.Chr4G00105350.1	*AT2G45540*
slo.H108236	Sjap.Chr1G00039520.1	*AT3G52155*
slo.H110499	Sjap.Chr1G00021330.1	*GLT1*
slo.H112048	Sjap.Chr1G00007910.1	*AT2G45500*
slo.H115999	Sjap.Chr2G00065300.1	*AT3G46960*
slo.H121412	Sjap.Chr8G00192430.1	*OCP3*
slo.H120361	Sjap.Chr8G00201070.1	*AT3G60370*
slo.H100985	Sjap.Chr4G00104970.1	*ATR*
slo.H105520	Sjap.Chr6G00146150.1	*AT3G02820*
slo.H116438	Sjap.Chr2G00069170.1	*AT5G37290*
slo.H126622	Sjap.Chr9G00229700.1	*3-Aug*
slo.H127738	Sjap.Chr11G00253150.1	*AT1G61850*
slo.H120576	Sjap.Chr8G00199520.1	*AT5G58370*
slo.H105855	Sjap.Chr7G00168700.1	*AT1G49380*
slo.H100829	Sjap.Chr4G00103500.1	*PPH*
slo.H112146	Sjap.Chr1G00007060.1	*GlcNAc1pUT1*
slo.H115560	Sjap.Chr2G00061280.1	*AT4G38020*
slo.H119012	Sjap.Chr8G00212050.1	*AT2G46560*
slo.H116104	Sjap.Chr2G00066240.1	*DRH1*
slo.H106170	Sjap.Chr7G00171400.1	*AT5G65490*
slo.H103653	Sjap.Chr6G00161470.1	*ATPI4K*
slo.H129284	Sjap.Chr3G00080810.1	*AT4G31170*
slo.H111700	Sjap.Chr1G00010730.1	*AT5G06260*
slo.H103528	Sjap.Chr6G00162720.1	*AT3G57880*
slo.H111284	Sjap.Chr1G00014580.1	*HCC1*
slo.H105435	Sjap.Chr6G00146760.1	*SDG40*
slo.H115986	Sjap.Chr2G00065170.1	*AT5G02860*
slo.H114387	Sjap.Chr2G00051630.1	*AT1G59660*
slo.H118435	Sjap.Chr10G00235890.1	*MOS1*
slo.H118265	Sjap.Chr10G00237230.1	*AT3G01380*
slo.H102561	Sjap.Chr4G00117520.1	*AT5G11980*
slo.H129666	Sjap.Chr3G00077680.1	*PAC*
slo.H121786	Sjap.Chr5G00143790.1	*AT1G22870*
slo.H104215	Sjap.Chr6G00156470.1	*CUL1*
slo.H100722	Sjap.Chr4G00102510.1	*AT3G51050*
slo.H105318	Sjap.Chr6G00147860.1	*AT5G37530*
slo.H123707	Sjap.Chr5G00128020.1	*AT2G39580*
slo.H109381	Sjap.Chr1G00029540.1	*AT5G55220*
slo.H110532	Sjap.Chr1G00021100.1	*AT2G41945*
slo.H130493	Sjap.Chr3G00095380.1	*AT1G05790*
slo.H102414	Sjap.Chr4G00116400.1	*AT5G11840*
slo.H105839	Sjap.Chr7G00168600.1	*DTX35*
slo.H100837	Sjap.Chr4G00103600.1	*AT1G67530*

slo.H106281	Sjap.Chr7G00172500.1	*AT5G66810*
slo.H117635	Sjap.Chr10G00241970.1	*GEX3*
slo.H133653	Sjap.Chr9G00231620.1	*AT5G58100*
slo.H114330	Sjap.Chr2G00051300.1	*DGD2*
slo.H121986	Sjap.Chr5G00142030.1	*UGP3*
slo.H130477	Sjap.Chr3G00095510.1	*CIB5*
slo.H127933	Sjap.Chr11G00251420.1	*XYLT*
slo.H119776	Sjap.Chr8G00205390.1	*AT1G08410*
slo.H113911	Sjap.Chr2G00048080.1	*ABCA12*
slo.H108563	Sjap.Chr1G00036790.1	*EMB1135*
slo.H119031	Sjap.Chr8G00211880.1	*AT5G28400*
slo.H108950	Sjap.Chr1G00033480.1	*AT3G21810*
slo.H110255	Sjap.Chr1G00023240.1	*AT4G23540*
slo.H122099	Sjap.Chr5G00141060.1	*MOS7*
slo.H118060	Sjap.Chr10G00238810.1	*AT3G28720*
slo.H102842	Sjap.Chr4G00119810.1	*AT3G14172*
slo.H121939	Sjap.Chr5G00142480.1	*SLP1*
slo.H101660	Sjap.Chr4G00110730.1	*NUB1*
slo.H125381	Sjap.Chr9G00219440.1	*AT5G23430*
slo.H101073	Sjap.Chr4G00105790.1	*AT4G09760*
slo.H120358	Sjap.Chr8G00201090.1	*SRS2*
slo.H105622	Sjap.Chr6G00145100.1	*PARC6*
slo.H102740	Sjap.Chr4G00119000.1	*XLG3*
slo.H103367	Sjap.Chr6G00164090.1	*AT1G67700*
slo.H112127	Sjap.Chr1G00007190.1	*AT4G10600*
slo.H119238	Sjap.Chr8G00210110.1	*AT4G01290*
slo.H124534	Sjap.Chr9G00212790.1	*LOV1*
slo.H122332	Sjap.Chr5G00139130.1	*AT3G07700*
slo.H123591	Sjap.Chr5G00128920.1	*PRMT6*
slo.H122667	Sjap.Chr5G00136250.1	*AT3G52210*
slo.H122500	Sjap.Chr5G00137630.1	*AT1G73170*
slo.H108495	Sjap.Chr1G00037180.1	*QRT1*
slo.H109165	Sjap.Chr1G00031520.1	*AT1G02670*
slo.H106554	Sjap.Chr7G00175040.1	*ATGRIP*
slo.H120015	Sjap.Chr8G00203320.1	*AT5G17250*
slo.H124417	Sjap.Chr5G00121720.1	*LNO1*
slo.H106439	Sjap.Chr7G00173980.1	*TIC21*
slo.H109211	Sjap.Chr1G00031060.1	*DEG14*
slo.H117528	Sjap.Chr10G00242770.1	*AT2G33680*
slo.H127899	Sjap.Chr11G00251730.1	*TOPP2*
slo.H113153	Sjap.Chr2G00041630.1	*HDG2*
slo.H131658	Sjap.Chr3G00084500.1	*AT4G17540*
slo.H102458	Sjap.Chr4G00116750.1	*BGAL12*
slo.H109526	Sjap.Chr1G00028670.1	*AT5G55520*

slo.H127872	Sjap.Chr11G00251960.1	*AT4G22290*
slo.H130666	Sjap.Chr3G00093700.1	*AT3G07530*
slo.H113111	Sjap.Chr2G00041310.1	*NOA1*
slo.H112929	Sjap.Chr2G00039750.1	*AT5G17670*
slo.H100776	Sjap.Chr4G00103030.1	*AT4G39160*
slo.H105688	Sjap.Chr6G00144570.1	*AT3G19990*
slo.H116483	Sjap.Chr2G00069500.1	*MCM3*
slo.H116017	Sjap.Chr2G00065460.1	*AT5G25040*
slo.H121855	Sjap.Chr5G00143210.1	*AT5G05310*
slo.H112969	Sjap.Chr2G00040070.1	*TRM9*
slo.H128602	Sjap.Chr11G00262340.1	*VAR2*
slo.H100379	Sjap.Chr4G00099660.1	*SBT4.12*
slo.H107238	Sjap.Chr7G00181810.1	*AT5G64160*
slo.H119530	Sjap.Chr8G00207550.1	*AT5G51880*
slo.H128504	Sjap.Chr11G00262980.1	*RFNR2*
slo.H112714	Sjap.Chr1G00002080.1	*ALN*
slo.H117797	Sjap.Chr10G00240380.1	*EDL2*
slo.H116841	Sjap.Chr10G00248720.1	*GC1*
slo.H120898	Sjap.Chr8G00197080.1	*AT2G05830*
slo.H124932	Sjap.Chr9G00216100.1	*AT5G63370*
slo.H117972	Sjap.Chr10G00239310.1	*AT3G55160*
slo.H105238	Sjap.Chr6G00148720.1	*THO2*
slo.H103390	Sjap.Chr6G00163890.1	*AT4G31570*
slo.H113199	Sjap.Chr2G00042070.1	*AT1G17640*
slo.H102845	Sjap.Chr4G00119830.1	*AT5G21280*
slo.H115792	Sjap.Chr2G00063140.1	*AT1G63330*
slo.H107319	Sjap.Chr7G00182390.1	*RECQSIM*
slo.H115592	Sjap.Chr2G00061620.1	*AT5G37360*
slo.H130721	Sjap.Chr3G00093250.1	*CRT3*
slo.H127834	Sjap.Chr11G00252300.1	*AT1G05060*
slo.H103274	Sjap.Chr6G00164980.1	*AT3G27180*
slo.H119168	Sjap.Chr8G00210720.1	*AT2G41600*
slo.H116579	Sjap.Chr2G00070340.1	*AT4G01860*
slo.H115855	Sjap.Chr2G00063780.1	*AT3G23080*
slo.H110000	Sjap.Chr1G00025250.1	*AT5G17840*
slo.H121074	Sjap.Chr8G00195580.1	*AT1G06710*
slo.H102329	Sjap.Chr4G00115730.1	*AT4G32820*
slo.H114543	Sjap.Chr2G00052570.1	*AT3G21465*
slo.H100108	Sjap.Chr4G00097130.1	*FC1*
slo.H126583	Sjap.Chr9G00229320.1	*AT1G63080*
slo.H108988	Sjap.Chr1G00033040.1	*AT1G36050*
slo.H102474	Sjap.Chr4G00116880.1	*AT3G57060*
slo.H126138	Sjap.Chr9G00225690.1	*OTP86*
slo.H121591	Sjap.Chr8G00190770.1	*AT3G61750*

slo.H100059	Sjap.Chr4G00096690.1	*CHB3*
slo.H121630	Sjap.Chr8G00190470.1	*AT5G14770*
slo.H121609	Sjap.Chr8G00190630.1	*ASP5*
slo.H129706	Sjap.Chr3G00077310.1	*RRP4*
slo.H109065	Sjap.Chr1G00032330.1	*XIJ*
slo.H107748	Sjap.Chr7G00185740.1	*Fes1A*
slo.H101663	Sjap.Chr4G00110790.1	*AT2G25280*
slo.H119827	Sjap.Chr8G00205010.1	*TTN1*
slo.H127735	Sjap.Chr11G00253190.1	*UBP10*
slo.H108243	Sjap.Chr1G00039470.1	*ACT1*
slo.H120254	Sjap.Chr8G00201810.1	*EDD1*
slo.H110618	Sjap.Chr1G00020370.1	*AT2G40980*
slo.H115853	Sjap.Chr2G00063760.1	*TZP*
slo.H121125	Sjap.Chr8G00195020.1	*AT1G25420*
slo.H122170	Sjap.Chr5G00140510.1	*RPL4*
slo.H116173	Sjap.Chr2G00066850.1	*AT2G20790*
slo.H103558	Sjap.Chr6G00162330.1	*AT1G70590*
slo.H114220	Sjap.Chr2G00049760.1	*AT3G61080*
slo.H118452	Sjap.Chr10G00235760.1	*AT5G57230*
slo.H101387	Sjap.Chr4G00108480.1	*FBW2*
slo.H120168	Sjap.Chr8G00202270.1	*AT1G65070*
slo.H108959	Sjap.Chr1G00033410.1	*NDR1*
slo.H125920	Sjap.Chr9G00224030.1	*SUA*
slo.H100313	Sjap.Chr4G00099070.1	*UBC8*
slo.H123649	Sjap.Chr5G00128530.1	*AT2G41640*
slo.H119538	Sjap.Chr8G00207510.1	*SSE1*
slo.H111978	Sjap.Chr1G00008570.1	*AT4G19440*
slo.H121774	Sjap.Chr5G00143940.1	*AT3G14170*
slo.H119257	Sjap.Chr8G00209940.1	*AT4G11350*
slo.H109275	Sjap.Chr1G00030430.1	*AT4G30020*
slo.H106752	Sjap.Chr7G00177000.1	*TTN7*
slo.H109568	Sjap.Chr1G00027920.1	*AT2G32160*
slo.H125924	Sjap.Chr9G00224060.1	*PAA2*
slo.H130315	Sjap.Chr3G00071700.1	*AT5G02860*
slo.H126701	Sjap.Chr9G00230410.1	*AT2G19260*
slo.H122436	Sjap.Chr5G00138210.1	*AT1G16800*
slo.H125213	Sjap.Chr9G00218050.1	*AT1G20380*
slo.H123626	Sjap.Chr5G00128690.1	*AT1G25500*
slo.H116779	Sjap.Chr10G00249260.1	*SOS4*
slo.H100899	Sjap.Chr4G00104150.1	*PBRP*
slo.H109153	Sjap.Chr1G00031600.1	*AT5G55060*
slo.H116706	Sjap.Chr10G00249830.1	*EDA7*
slo.H121402	Sjap.Chr8G00192520.1	*AT5G20170*
slo.H120563	Sjap.Chr8G00199640.1	*S1P*

slo.H110529	Sjap.Chr1G00021150.1	*AT3G44050*
slo.H121840	Sjap.Chr5G00143320.1	*AT3G56330*
slo.H109210	Sjap.Chr1G00031070.1	*EMB2729*
slo.H117634	Sjap.Chr10G00241980.1	*AT3G29280*
slo.H117029	Sjap.Chr10G00246720.1	*CPSF73-I*
slo.H103700	Sjap.Chr6G00161050.1	*EX1*
slo.H127521	Sjap.Chr11G00255010.1	*PETC*
slo.H107040	Sjap.Chr7G00179130.1	*AT5G15880*
slo.H102980	Sjap.Chr4G00120840.1	*AT3G13225*
slo.H130992	Sjap.Chr3G00090570.1	*RAD50*
slo.H103826	Sjap.Chr6G00159890.1	*ZAT*
slo.H105547	Sjap.Chr6G00145850.1	*AT2G40640*
slo.H130709	Sjap.Chr3G00093340.1	*PPI1*
slo.H121952	Sjap.Chr5G00142340.1	*TAF15b*
slo.H123772	Sjap.Chr5G00127470.1	*AT2G32840*
slo.H122112	Sjap.Chr5G00140950.1	*CPL2*
slo.H126685	Sjap.Chr9G00230250.1	*AT4G31570*
slo.H124764	Sjap.Chr9G00214640.1	*PTP1*
slo.H111155	Sjap.Chr1G00015910.1	*AT5G50840*
slo.H109986	Sjap.Chr1G00025360.1	*AT4G26370*
slo.H128396	Sjap.Chr11G00263780.1	*PTAC12*
slo.H112019	Sjap.Chr1G00008130.1	*HEXO2*
slo.H117793	Sjap.Chr10G00240410.1	*SAMC1*
slo.H102182	Sjap.Chr4G00114540.1	*GLDH*
slo.H130706	Sjap.Chr3G00093360.1	*AT1G65270*
slo.H100093	Sjap.Chr4G00097000.1	*AT5G41060*
slo.H112974	Sjap.Chr2G00040120.1	*AT1G31500*
slo.H116747	Sjap.Chr10G00249540.1	*AT3G45020*
slo.H118232	Sjap.Chr10G00237570.1	*AT1G70150*
slo.H126724	Sjap.Chr9G00230620.1	*AT5G14770*
slo.H122603	Sjap.Chr5G00136790.1	*CYP704A1*
slo.H106643	Sjap.Chr7G00176130.1	*LPA3*
slo.H112596	Sjap.Chr1G00003130.1	*AT5G53920*
slo.H122148	Sjap.Chr5G00140710.1	*AT5G41330*
slo.H124323	Sjap.Chr5G00122630.1	*AT3G32930*
slo.H119144	Sjap.Chr8G00210860.1	*AE7*
slo.H130161	Sjap.Chr3G00073090.1	*NRPB9B*
slo.H124797	Sjap.Chr9G00214880.1	*AT1G35660*

Supplementary Table S13. The positive selection genes with KEGG annotations.

PSG Genes	Genes name	Map ID	Pathway

slo.H108988	AT1G36050	map04141	Protein processing in endoplasmic reticulum
slo.H109065	XIJ	map05130	Pathogenic Escherichia coli infection
slo.H109210	EMB2729	map00500	Starch and sucrose metabolism
slo.H109688	CER7	map03018	RNA degradation
slo.H110528	OVA2	map00970	Aminoacyl-tRNA biosynthesis
slo.H111155	AT5G50840	map00900	Terpenoid backbone biosynthesis
slo.H111349	AT3G02760	map00970	Aminoacyl-tRNA biosynthesis
slo.H111700	AT5G06260	map03018	RNA degradation
slo.H111966	TPR9	map00310	Lysine degradation
slo.H112019	HEXO2	map04142	Lysosome
		map00531	Glycosaminoglycan degradation
		map00513	Various types of N-glycan biosynthesis
		map00520	Amino sugar and nucleotide sugar metabolism
		map00511	Other glycan degradation
		map00603	Glycosphingolipid biosynthesis - globo and isoglobo series
		map00604	Glycosphingolipid biosynthesis - ganglio series
slo.H112634	SMG7	map03015	mRNA surveillance pathway
slo.H112714	ALN	map00230	Purine metabolism
slo.H112974	AT1G31500	map03018	RNA degradation
slo.H113043	AT4G19130	map03030	DNA replication
		map03460	Fanconi anemia pathway
		map03440	Homologous recombination
		map03430	Mismatch repair
		map03420	Nucleotide excision repair
slo.H114387	AT1G59660	map05164	Influenza A
		map03013	RNA transport
slo.H115581	AT5G38890	map03018	RNA degradation
slo.H115792	AT1G63330	map03460	Fanconi anemia pathway
slo.H115986	AT5G02860	map03460	Fanconi anemia pathway
slo.H115999	AT3G46960	map03018	RNA degradation
slo.H116173	AT2G20790	map04142	Lysosome
slo.H116394	AT5G55070	map00640	Propanoate metabolism
		map00280	Valine, leucine and isoleucine degradation
slo.H116483	MCM3	map04113	Meiosis - yeast
		map04111	Cell cycle - yeast
		map03030	DNA replication
		map04110	Cell cycle
slo.H116579	AT4G01860	map04136	Autophagy - other
		map04714	Thermogenesis
		map04150	mTOR signaling pathway
		map04140	Autophagy - animal
		map04151	PI3K-Akt signaling pathway
		map04138	Autophagy - yeast

slo.H116747	AT3G45020	map03010	Ribosome
slo.H116779	SOS4	map00750	Vitamin B6 metabolism
slo.H116967	AT3G17920	map05016	Huntington disease
slo.H117972	AT3G55160	map00563	Glycosylphosphatidylinositol (GPI)-anchor biosynthesis
slo.H118265	AT3G01380	map00563	Glycosylphosphatidylinositol (GPI)-anchor biosynthesis
slo.H118700	AT3G62310	map03040	Spliceosome
slo.H119120	TUB8	map05165	Human papillomavirus infection
slo.H119257	AT4G11350	map00514	Other types of O-glycan biosynthesis
		map00062	Fatty acid elongation
slo.H119342	AT1G18410	map01040	Biosynthesis of unsaturated fatty acids
		map04913	Ovarian steroidogenesis
slo.H119538	SSE1	map04146	Peroxisome
slo.H119776	AT1G08410	map03008	Ribosome biogenesis in eukaryotes
		map05146	Amoebiasis
slo.H119789	ARGAH2	map00330	Arginine and proline metabolism
		map00220	Arginine biosynthesis
slo.H119827	TTN1	map03040	Spliceosome
slo.H120015	AT5G17250	map00563	Glycosylphosphatidylinositol (GPI)-anchor biosynthesis
slo.H120563	S1P	map04141	Protein processing in endoplasmic reticulum
slo.H120898	AT2G05830	map00270	Cysteine and methionine metabolism
slo.H120969	TRZ4	map03013	RNA transport
slo.H120980	AT4G32790	map00534	Glycosaminoglycan biosynthesis - heparan sulfate / heparin
slo.H121074	AT1G06710	map03460	Fanconi anemia pathway
slo.H121402	AT5G20170	map04919	Thyroid hormone signaling pathway
slo.H121562	AT5G07770	map04310	Wnt signaling pathway
slo.H121630	AT5G14770	map03460	Fanconi anemia pathway
slo.H121952	TAF15b	map05202	Transcriptional misregulation in cancer
slo.H122099	MOS7	map03013	RNA transport
slo.H122170	RPL4	map03010	Ribosome
slo.H122251	AT1G05670	map03460	Fanconi anemia pathway
slo.H122436	AT1G16800	map03040	Spliceosome
slo.H122667	AT3G52210	map03015	mRNA surveillance pathway
slo.H123626	AT1G25500	map05231	Choline metabolism in cancer
slo.H123635	AT1G63080	map03460	Fanconi anemia pathway
		map05016	Huntington disease
		map04932	Non-alcoholic fatty liver disease (NAFLD)
		map00190	Oxidative phosphorylation
slo.H123897	AT3G58470	map05010	Alzheimer disease
		map04714	Thermogenesis
		map05012	Parkinson disease

		map04723	Retrograde endocannabinoid signaling
slo.H124176	FACE2	map00900	Terpenoid backbone biosynthesis
slo.H124417	LNO1	map03013	RNA transport
slo.H124764	PTP1	map02020	Two-component system
slo.H124876	AT1G18340	map03022	Basal transcription factors
		map05203	Viral carcinogenesis
		map03420	Nucleotide excision repair
slo.H100059	CHB3	map04714	Thermogenesis
		map05225	Hepatocellular carcinoma
slo.H100313	UBC8	map04120	Ubiquitin mediated proteolysis
slo.H100899	PBRP	map03022	Basal transcription factors
		map05203	Viral carcinogenesis
		map05017	Spinocerebellar ataxia
slo.H100985	ATR	map03015	mRNA surveillance pathway
slo.H101073	AT4G09760	map00564	Glycerophospholipid metabolism
		map05231	Choline metabolism in cancer
slo.H101267	AT5G14850	map00563	Glycosylphosphatidylinositol (GPI)-anchor biosynthesis
slo.H125109	AT1G21580	map04360	Axon guidance
		map04015	Rap1 signaling pathway
		map04144	Endocytosis
		map04530	Tight junction
		map04391	Hippo signaling pathway - fly
		map05165	Human papillomavirus infection
		map04390	Hippo signaling pathway
slo.H126185	AT5G63680	map00010	Glycolysis / Gluconeogenesis
		map05203	Viral carcinogenesis
		map05165	Human papillomavirus infection
		map04930	Type II diabetes mellitus
		map00620	Pyruvate metabolism
		map00230	Purine metabolism
		map04922	Glucagon signaling pathway
		map05230	Central carbon metabolism in cancer
slo.H126724	AT5G14770	map03460	Fanconi anemia pathway
slo.H102182	GLDH	map00053	Ascorbate and aldarate metabolism
slo.H102416	GLDP1	map00260	Glycine, serine and threonine metabolism
		map00630	Glyoxylate and dicarboxylate metabolism
slo.H102458	BGAL12	map00052	Galactose metabolism
		map04142	Lysosome
		map00531	Glycosaminoglycan degradation
		map00600	Sphingolipid metabolism
		map00511	Other glycan degradation
		map00604	Glycosphingolipid biosynthesis - ganglio series
slo.H127115	AT5G27410	map00770	Pantothenate and CoA biosynthesis

		map00290	Valine, leucine and isoleucine biosynthesis
		map00280	Valine, leucine and isoleucine degradation
		map00270	Cysteine and methionine metabolism
		map00966	Glucosinolate biosynthesis
		map04014	Ras signaling pathway
		map00591	Linoleic acid metabolism
		map04975	Fat digestion and absorption
		map04972	Pancreatic secretion
slo.H127738	AT1G61850	map00592	alpha-Linolenic acid metabolism
		map00590	Arachidonic acid metabolism
		map04270	Vascular smooth muscle contraction
		map00564	Glycerophospholipid metabolism
		map00565	Ether lipid metabolism
		map04136	Autophagy - other
slo.H128913	APG7	map04216	Ferroptosis
		map04140	Autophagy - animal
		map04138	Autophagy - yeast
slo.H129706	RRP4	map03018	RNA degradation
slo.H130161	NRPB9B	map05016	Huntington disease
		map03020	RNA polymerase
		map04072	Phospholipase D signaling pathway
slo.H130315	AT5G02860	map04080	Neuroactive ligand-receptor interaction
		map04962	Vasopressin-regulated water reabsorption
		map04925	Aldosterone synthesis and secretion
slo.H130493	AT1G05790	map04745	Phototransduction - fly
		map04723	Retrograde endocannabinoid signaling
		map05170	Human immunodeficiency virus 1 infection
		map05169	Epstein-Barr virus infection
		map04612	Antigen processing and presentation
		map05168	Herpes simplex virus 1 infection
slo.H130721	CRT3	map05142	Chagas disease (American trypanosomiasis)
		map04145	Phagosome
		map04141	Protein processing in endoplasmic reticulum
		map05166	Human T-cell leukemia virus 1 infection
		map05163	Human cytomegalovirus infection
		map04218	Cellular senescence
slo.H130992	RAD50	map03450	Non-homologous end-joining
		map03440	Homologous recombination
		map00010	Glycolysis / Gluconeogenesis
		map00640	Propanoate metabolism
slo.H102991	ACS	map00680	Methane metabolism
		map00720	Carbon fixation pathways in prokaryotes
		map00620	Pyruvate metabolism
		map00630	Glyoxylate and dicarboxylate metabolism

slo.H103330	POLA2	map03030	DNA replication
slo.H103374	EMB2423	map04150	mTOR signaling pathway
		map03460	Fanconi anemia pathway
slo.H103528	AT3G57880	map00564	Glycerophospholipid metabolism
		map04371	Apelin signaling pathway
		map00562	Inositol phosphate metabolism
		map04136	Autophagy - other
		map05152	Tuberculosis
		map04138	Autophagy - yeast
		map05167	Kaposi sarcoma-associated herpesvirus infection
slo.H103653	ATPI4K	map05131	Shigellosis
		map04070	Phosphatidylinositol signaling system
		map05016	Huntington disease
		map05132	Salmonella infection
		map05017	Spinocerebellar ataxia
		map04145	Phagosome
		map04140	Autophagy - animal
slo.H103677	AT5G26680	map03420	Nucleotide excision repair
slo.H103684	PEX14	map04146	Peroxisome
slo.H103719	DWA3	map03040	Spliceosome
slo.H103763	AT3G26480	map03008	Ribosome biogenesis in eukaryotes
slo.H103911	AT4G27480	map00534	Glycosaminoglycan biosynthesis - heparan sulfate / heparin
		map00532	Glycosaminoglycan biosynthesis - chondroitin sulfate / dermatan sulfate
		map04113	Meiosis - yeast
		map04120	Ubiquitin mediated proteolysis
		map04111	Cell cycle - yeast
slo.H104215	CUL1	map04914	Progesterone-mediated oocyte maturation
		map05166	Human T-cell leukemia virus 1 infection
		map04110	Cell cycle
		map04114	Oocyte meiosis
slo.H105238	THO2	map03040	Spliceosome
		map03013	RNA transport
slo.H105391	AT2G33680	map03460	Fanconi anemia pathway
slo.H105435	SDG40	map02010	ABC transporters
slo.H105544	AT1G09130	map04212	Longevity regulating pathway - worm
		map04112	Cell cycle - Caulobacter
		map00513	Various types of N-glycan biosynthesis
slo.H105610	STT3A	map04141	Protein processing in endoplasmic reticulum
		map00510	N-Glycan biosynthesis
slo.H106752	TTN7	map04111	Cell cycle - yeast
slo.H107319	RECQSIM	map03460	Fanconi anemia pathway
		map03440	Homologous recombination

slo.H107748	Fes1A	map04141	Protein processing in endoplasmic reticulum
		map04611	Platelet activation
		map04015	Rap1 signaling pathway
		map05164	Influenza A
		map05130	Pathogenic Escherichia coli infection
		map04810	Regulation of actin cytoskeleton
		map05225	Hepatocellular carcinoma
		map04745	Phototransduction - fly
		map05135	Yersinia infection
		map04390	Hippo signaling pathway
		map05131	Shigellosis
		map04921	Oxytocin signaling pathway
		map05418	Fluid shear stress and atherosclerosis
		map04670	Leukocyte transendothelial migration
		map05132	Salmonella infection
slo.H108243	ACT1	map05410	Hypertrophic cardiomyopathy (HCM)
		map04145	Phagosome
		map04510	Focal adhesion
		map05100	Bacterial invasion of epithelial cells
		map05416	Viral myocarditis
		map04530	Tight junction
		map04714	Thermogenesis
		map04520	Adherens junction
		map05412	Arrhythmogenic right ventricular cardiomyopathy (ARVC)
		map05110	Vibrio cholerae infection
		map04210	Apoptosis
		map05414	Dilated cardiomyopathy (DCM)
		map04391	Hippo signaling pathway - fly
		map05205	Proteoglycans in cancer
		map04919	Thyroid hormone signaling pathway
		map04714	Thermogenesis
slo.H108370	SRT2	map00760	Nicotinate and nicotinamide metabolism
		map05230	Central carbon metabolism in cancer
slo.H108444	AT4G19900	map00601	Glycosphingolipid biosynthesis - lacto and neolacto series
		map00603	Glycosphingolipid biosynthesis - globo and isoglobo series

Supplementary Table S14. WGD gene trees with frequently occurring topologies.

Topology	Count

((((S.japonica,S.longa),C.chinensis),A.trifoliata),(((S.japonica,S.longa),C.chinensis),A.trifoliata))	21
((((S.japonica,S.longa),C.chinensis),A.trifoliata),((A.trifoliata,C.chinensis),(S.japonica,S.longa)))	6
((((S.japonica,S.longa),A.trifoliata),C.chinensis),(((S.japonica,S.longa),C.chinensis),A.trifoliata))	6
((((S.japonica,S.longa),C.chinensis),((S.japonica,S.longa),C.chinensis)),(A.trifoliata,A.trifoliata))	5
((((S.japonica,S.longa),A.trifoliata),C.chinensis),(((S.japonica,S.longa),C.chinensis),A.trifoliata))	4
((((S.japonica,S.longa),A.trifoliata),C.chinensis),(((S.japonica,S.longa),A.trifoliata),C.chinensis))	4
((((S.japonica,S.longa),C.chinensis),A.trifoliata),(((S.japonica,S.longa),A.trifoliata),C.chinensis))	4
((((S.japonica,S.longa),A.trifoliata),C.chinensis),((A.trifoliata,C.chinensis),(S.japonica,S.longa)))	4
((((S.japonica,S.longa),C.chinensis),A.trifoliata),((A.trifoliata,C.chinensis),(S.japonica,S.longa)))	4
((((S.japonica,S.longa),C.chinensis),A.trifoliata),((A.trifoliata,C.chinensis),(S.japonica,S.longa)))	4
((((S.japonica,S.longa),(S.japonica,S.longa)),(C.chinensis,C.chinensis)),(A.trifoliata,A.trifoliata))	3
(((((S.japonica,S.longa),C.chinensis),C.chinensis),(S.japonica,S.longa)),(A.trifoliata,A.trifoliata))	3
((((C.chinensis,C.chinensis),A.trifoliata),(S.japonica,S.longa)),((S.japonica,S.longa),A.trifoliata))	3
(((((S.japonica,S.longa),C.chinensis),(S.japonica,S.longa)),C.chinensis),(A.trifoliata,A.trifoliata))	2
((((S.japonica,S.longa),C.chinensis),A.trifoliata),((A.trifoliata,C.chinensis),(S.japonica,S.longa)))	2
(((A.trifoliata,C.chinensis),(S.japonica,S.longa)),((A.trifoliata,C.chinensis),(S.japonica,S.longa)))	2
(((((S.japonica,S.longa),C.chinensis),(S.japonica,S.longa)),C.chinensis),(A.trifoliata,A.trifoliata))	2
(((((S.japonica,S.longa),C.chinensis),C.chinensis),(S.japonica,S.longa)),(A.trifoliata,A.trifoliata))	2
((((C.chinensis,C.chinensis),A.trifoliata),A.trifoliata),((S.japonica,S.longa),(S.japonica,S.longa)))	2
((((S.japonica,S.longa),C.chinensis),(S.japonica,S.longa)),((A.trifoliata,C.chinensis),A.trifoliata))	1
((((S.japonica,S.longa),A.trifoliata),A.trifoliata),((C.chinensis,C.chinensis),(S.japonica,S.longa)))	1
((((S.japonica,S.longa),A.trifoliata),(A.trifoliata,C.chinensis)),((S.japonica,S.longa),C.chinensis))	1

(((((S.japonica,S.longa),C.chinensis),C.chinensis),A.trifoliata),((S.japonica,S.longa),A.trifoliata))	1
(((((S.japonica,S.longa),C.chinensis),A.trifoliata),C.chinensis),((S.japonica,S.longa),A.trifoliata))	1
(((A.trifoliata,C.chinensis),(A.trifoliata,C.chinensis)),((S.japonica,S.longa),(S.japonica,S.longa)))	1
((((S.japonica,S.longa),A.trifoliata),A.trifoliata),((C.chinensis,C.chinensis),(S.japonica,S.longa)))	1
(((((S.japonica,S.longa),(S.japonica,S.longa)),C.chinensis),A.trifoliata),(A.trifoliata,C.chinensis))	1
((((S.japonica,S.longa),C.chinensis),(S.japonica,S.longa)),((A.trifoliata,C.chinensis),A.trifoliata))	1
((((S.japonica,S.longa),C.chinensis),(S.japonica,S.longa)),((A.trifoliata,C.chinensis),A.trifoliata))	1
((((S.japonica,S.longa),(S.japonica,S.longa)),C.chinensis),((A.trifoliata,C.chinensis),A.trifoliata))	1
((((C.chinensis,C.chinensis),(S.japonica,S.longa)),(S.japonica,S.longa)),(A.trifoliata,A.trifoliata))	1
((((A.trifoliata,A.trifoliata),(S.japonica,S.longa)),C.chinensis),((S.japonica,S.longa),C.chinensis))	1
(((A.trifoliata,C.chinensis),(A.trifoliata,C.chinensis)),((S.japonica,S.longa),(S.japonica,S.longa)))	1
((((S.japonica,S.longa),C.chinensis),A.trifoliata),((A.trifoliata,C.chinensis),(S.japonica,S.longa)))	1
((((A.trifoliata,S.japonica),S.longa),A.trifoliata),((C.chinensis,C.chinensis),(S.japonica,S.longa)))	1
((((S.japonica,S.longa),A.trifoliata),C.chinensis),(((S.japonica,S.longa),C.chinensis),A.trifoliata))	1
((((S.japonica,S.longa),A.trifoliata),C.chinensis),((A.trifoliata,C.chinensis),(S.japonica,S.longa)))	1
((((S.japonica,S.longa),C.chinensis),A.trifoliata),(((S.japonica,S.longa),A.trifoliata),C.chinensis))	1
(((((S.japonica,S.longa),C.chinensis),C.chinensis),A.trifoliata),((S.japonica,S.longa),A.trifoliata))	1

Supplementary Table S15. Statistics of Protein domain annotation in the *Stephania longa* and other species analyzed with Pfam-A database.

Species name	Domain count
Stephania longa	105,142
Stephania japonica	88,139
Papaver somniferum	235,539
Macleaya cordata	88,483

Coptis chinensis	129,098
Aquilegia coerulea	101,483
Akebia trifoliata	92,765
Kingdonia uniflora	121,257
Nelumbo nucifera	105,019

Supplementary Table S16. Significantly expanded domains in the genus *Stephania.* The mean Z-score refers to the average Z-score of two *Stephania* species.

Domain Name	*S. longa*	*S. japonica*	*M. cordata*	*K. uniflora*	*C. chinensis*	*N. nucifera*	*A.coerulea*	*A. trifoliata*	*P. somniferum*	Mean Z-score
DUF6866_N	4	94	0	2	0	0	0	0	0	69.61
BING4CT	6	70	1	2	3	3	3	1	5	27.39
Invas_SpaK	8	6	0	0	0	1	0	0	0	19.60
RIP	10	7	0	1	0	0	0	1	0	18.18
Catalase	10	24	4	3	3	2	3	2	5	14.00
Peptidase_C54	17	5	3	3	2	2	3	2	4	11.84
Patched	8	12	2	2	1	3	1	1	2	11.84
DUF3489	13	5	0	1	2	0	0	0	0	11.77
VCX_VCY	7	4	0	0	0	1	0	1	0	11.54
YebO	15	7	1	2	1	3	1	0	2	10.59
Thioredoxin_12	6	3	1	1	1	1	1	1	2	9.59
DUF4659	4	3	0	0	0	0	0	0	1	9.59
DUF6293	5	4	0	1	0	0	0	0	1	9.33
DUF3099	6	4	0	1	1	0	0	1	0	9.24
FAM186A-B_N	3	9	1	2	1	2	1	1	2	9.24
DUF778	12	17	2	4	4	5	4	4	6	9.21
SPACA9	9	5	2	0	1	0	0	1	1	8.98
LARA_dom	6	5	0	1	2	1	1	1	1	8.42
KinB_sensor	3	6	1	1	1	0	1	1	0	8.38
SHPRH_helical-1st	17	11	4	2	6	4	4	3	5	8.37
MIEAP	5	4	0	1	1	1	0	0	0	8.23
U79_P34	4	5	1	0	1	0	1	0	0	8.23
Integrase_2	6	2	0	1	0	0	0	0	1	8.22
DnaB_bind	3	5	0	1	0	0	0	1	0	8.22
LBP_M	4	2	0	0	0	1	0	0	0	8.16
SOXp	4	2	0	0	0	0	1	0	0	8.16
DUF3243	4	2	1	0	0	0	0	0	0	8.16
LLC1	3	3	0	0	1	0	0	0	0	8.16

Umbravirus_LDM	3	3	0	0	1	0	0	0	0	8.16
PBS_linker_poly	4	9	0	2	1	0	0	1	0	8.14
ANXA2R	5	4	1	1	0	1	0	0	1	7.94
NodZ	5	4	1	0	0	1	1	1	0	7.94
TAL_effector	10	7	1	2	1	3	0	1	2	7.83
Kunitz_legume	31	28	9	6	11	12	4	11	9	7.66
MAP65_ASE1	30	38	6	9	8	10	8	5	16	7.59
PTS_IIB	6	6	0	0	0	1	1	1	2	7.55
IgGFc_binding	5	2	0	0	1	0	0	0	1	7.12
Antitox_RHH	4	3	0	1	0	0	1	0	0	7.12
dbPDZ_assoc	4	3	1	0	0	0	1	0	0	7.12
DUF3836	4	3	0	1	1	0	0	0	0	7.12
DUF5304	3	4	0	0	1	0	0	1	0	7.12
PP1	17	10	1	4	4	2	5	4	5	7.09
Peptidase_C97	39	41	8	12	15	13	8	10	20	7.01
Atrophin-1	25	21	9	6	4	4	7	7	11	6.85
DUF6052	3	2	0	0	1	0	0	0	0	6.74
DUF4532	3	2	0	1	0	0	0	0	0	6.74
Focadhesin	3	2	0	0	1	0	0	0	0	6.74
Atthog	2	3	0	1	0	0	0	0	0	6.74
AbiGii_2	2	3	0	0	0	0	1	0	0	6.74
23S_rRNA_IVP	2	3	0	0	0	0	0	0	1	6.74
NEMP	17	10	3	2	6	2	4	5	5	6.62
CDC4_D	7	7	1	0	1	0	1	1	3	6.48
Surface_antigen	6	4	0	0	2	0	1	0	0	6.28
LAGLIDADG_1	5	2	0	0	1	0	1	0	1	6.21
DUF2970	7	5	0	1	2	0	1	0	2	6.17
DUF3198	5	2	1	0	1	1	0	1	1	6.17
Urease_gamma	5	4	2	1	2	1	2	2	2	6.17
Luteo_P1-P2	6	4	1	0	1	1	0	0	2	6.12
DUF1163	4	6	0	1	0	0	1	0	2	6.08

NST1	12	8	0	4	3	1	2	1	0	6.02
DUF5038	3	3	0	0	1	0	0	1	0	6.01
DUF4154	3	3	0	1	1	0	0	0	0	6.01
DUF792	3	3	0	1	0	0	1	0	0	6.01
GD_AH_second	3	3	0	1	1	0	0	0	0	6.01
Glyco_hydro_44	4	2	0	0	1	0	0	1	0	6.01
DUF5115	2	4	1	0	0	0	1	0	0	6.01
E1_FCCH	9	6	2	3	2	1	2	2	4	5.92
DUF4423	5	4	2	2	1	2	1	2	1	5.92
Ceramidse_alk_C	3	6	1	1	2	1	2	2	2	5.92
Fungus-induced	6	5	2	1	0	2	1	2	2	5.59
Phage_Mu_Gp27	5	9	3	0	1	2	1	2	0	5.55
Col_cuticle_N	9	5	0	1	0	3	0	0	2	5.46
DUF6146	5	12	0	1	4	0	2	0	1	5.43
CCDC34	4	5	1	0	0	1	0	1	2	5.41
RicinB_lectin_2	16	10	3	1	3	1	2	2	7	5.40
ATP12	3	3	1	1	1	1	1	1	2	5.31
ANM3_zf-C2H2	2	2	0	0	0	1	0	0	0	5.31
ORC1_wHTH	2	2	0	1	0	0	0	0	0	5.31
DUF6722	2	2	0	0	1	0	0	0	0	5.31
DUF6385	2	2	0	0	0	0	0	1	0	5.31
DUF6113	2	2	0	0	0	0	0	1	0	5.31
IL17R_fnIII_D1	2	2	0	1	0	0	0	0	0	5.31
S8_pro-domain	2	2	0	1	0	0	0	0	0	5.31
DUF5018	2	2	0	0	0	1	0	0	0	5.31
DUF4920	2	2	0	0	0	0	0	0	1	5.31
DUF4831	2	2	0	0	0	0	0	1	0	5.31
DUF4327	2	2	0	0	0	1	0	0	0	5.31
NPH-II	2	2	0	0	0	0	0	1	0	5.31
Cas_Csy2	2	2	0	0	0	0	0	0	1	5.31
DUF1924	2	2	1	0	0	0	0	0	0	5.31
SNN_linker	2	2	0	0	1	0	0	0	0	5.31
DUF1824	2	2	1	0	0	0	0	0	0	5.31

NTNH_C	2	2	0	0	0	0	0	1	0	5.31
Psu	2	2	0	1	0	0	0	0	0	5.31
Extensin-like_C	2	2	0	0	0	0	0	1	0	5.31
DUF417	2	2	0	0	0	0	0	0	1	5.31
EII-GUT	2	2	0	0	1	0	0	0	0	5.31
PPV_E1_N	2	2	0	0	0	0	1	0	0	5.31
MOSC	6	4	2	3	3	2	2	2	3	5.20
DUF6588	3	3	1	1	0	1	0	0	0	5.20
Asparaginase_C	3	3	1	0	0	1	0	0	1	5.20
NiFeSe_Hases	3	3	0	1	1	1	0	1	1	5.06
DUF6142	3	3	1	1	0	0	1	1	0	4.91
DUF5024	3	3	0	0	1	0	1	1	1	4.91
Picorna_P3A	4	2	0	0	1	1	0	1	1	4.91
DUF5054	5	3	0	1	0	2	0	0	0	4.90
Sterol-sensing	3	7	1	0	2	2	2	1	2	4.90
TERT_C	4	3	1	1	2	1	1	2	1	4.90
Ebp1_C	3	2	0	0	0	1	0	0	1	4.90
ATG29_N	3	2	0	0	0	1	1	0	0	4.90
DUF3759	3	2	0	1	0	0	0	1	0	4.90
Muramidase	3	4	1	2	1	1	1	1	2	4.90
DUF3310	3	2	0	1	0	0	0	1	0	4.90
DUF2586	3	2	0	1	1	0	0	0	0	4.90
EppA_BapA	3	2	0	1	0	0	1	0	0	4.90
DMT_6	3	2	0	0	1	0	0	0	1	4.90
PMT	3	2	0	1	0	0	1	0	0	4.90
Urease_beta	3	4	1	2	1	1	1	1	2	4.90
DUF6833	2	3	0	0	1	0	1	0	0	4.90
Calpain_inhib	2	3	0	0	1	0	0	0	1	4.90
Ion_trans_N	6	5	0	0	0	0	0	0	3	4.83
ETC_C1_NDUFA5	7	6	2	2	4	2	2	1	3	4.79
Apis_Csd	4	7	2	1	1	1	3	0	1	4.79
L27	5	3	0	1	2	0	1	1	0	4.69
CytoC_RC	5	3	0	1	0	1	1	0	2	4.69
MHC2-interact	4	4	1	1	2	0	0	1	0	4.69
CCDC22_N	5	4	1	1	0	2	1	2	2	4.59

Glyco_hydro_4C	5	4	0	2	2	1	1	1	2	4.59
DUF575	4	3	0	0	0	0	0	0	2	4.59
TssN	11	10	3	6	5	6	5	3	6	4.53
Deltameth_res	5	4	0	0	2	0	2	1	1	4.37
SRP1_TIP1	5	4	0	0	2	0	0	0	2	4.35
BRR2_plug	13	10	3	5	4	1	3	2	7	4.31
PgpA	4	5	2	2	1	2	0	2	1	4.22
RnlA-toxin_DBD	3	2	0	1	0	1	1	0	0	4.19
ADDT	3	2	0	1	0	0	0	1	1	4.19
TAtT	3	2	1	1	0	0	0	1	0	4.19
NCF1_PBR_AIR	3	2	0	0	0	0	1	1	1	4.19
SEEEED	3	2	0	0	1	0	1	0	1	4.19
DNA_pol3_a_NI	3	2	0	0	1	0	1	0	1	4.19
Flavodoxin_4	3	2	0	1	1	0	1	0	0	4.19
DFF40	3	2	0	0	1	0	1	0	1	4.19
DUF2620	2	3	0	1	0	1	0	0	1	4.19
Clr2	2	3	1	1	0	0	0	1	0	4.19
Pea-VEAacid	2	3	1	1	0	0	1	0	0	4.19
Pkr1	9	8	0	3	5	3	3	2	3	4.18
OAD_gamma	18	12	7	4	5	6	11	6	6	4.16
DUF3439	27	25	13	5	6	5	15	6	13	4.14
PH_17	4	3	1	0	1	2	0	1	1	4.14
BEN	4	3	1	1	1	2	0	0	1	4.14
DUF5493	5	6	0	0	1	2	0	0	3	4.13
ADD_DNMT3	8	5	4	1	2	1	0	2	2	4.12
Ricin_B_lectin	16	13	5	6	5	5	11	5	6	4.12
tRNA_U5-meth_tr	28	23	14	6	13	7	9	13	16	4.08
RNA_polI_A34	9	5	3	3	3	1	1	1	4	4.06
TMEM141	6	5	2	2	2	2	1	2	4	4.03
FAF1	6	5	2	3	2	2	4	2	3	4.02
HemY_N	31	22	12	8	10	8	15	11	19	3.98
GDH_ACT3	3	5	0	1	2	0	1	1	2	3.97

Glyco_trans _4_2	5	7	2	3	2	3	2	1	4	3.95
3-dmu-9_3-mt	3	2	0	1	1	1	1	1	0	3.95
RNA_pol_R pb8	3	4	2	2	1	2	1	2	2	3.95
ANAPC9	2	3	1	1	1	1	0	0	1	3.95
DUF6435	3	2	1	1	1	1	0	0	0	3.90
DUF6329	3	2	0	1	1	0	1	0	1	3.90
DUF6203	3	4	1	2	2	2	1	1	2	3.90
T6_Ig_like	3	2	0	1	0	1	1	0	1	3.90
FRQ	3	2	0	0	1	1	1	1	0	3.90
E_Pc_C	3	2	1	1	0	1	0	0	1	3.90
BppU_N	3	3	0	0	0	0	0	0	2	3.88
REV	7	4	0	2	1	0	3	0	2	3.87
LRAT	28	31	7	15	18	15	10	8	18	3.86
DUF6105	2	2	1	0	1	0	0	0	0	3.79
Acyltransf_2	2	2	0	0	1	1	0	0	0	3.79
DUF5975	2	2	0	1	1	0	0	0	0	3.79
DUF5901	2	2	0	0	0	1	0	0	1	3.79
Rv3651-like_N	2	2	1	0	0	1	0	0	0	3.79
RecC_C	2	2	0	1	0	0	1	0	0	3.79
HypF_C	2	2	1	1	0	0	0	0	0	3.79
OSK	2	2	0	0	1	1	0	0	0	3.79
DUF4699	2	2	0	0	0	0	1	0	1	3.79
TMEM71	2	2	0	1	0	0	1	0	0	3.79
FAM183	2	2	0	0	0	0	0	1	1	3.79
YbgS	2	2	0	0	1	0	1	0	0	3.79
RE_ScaI	2	2	0	1	0	1	0	0	0	3.79
PHAT	2	2	0	0	0	0	0	1	1	3.79
PSCyt2	2	2	0	0	0	1	0	0	1	3.79
Osteoregulin	2	2	0	1	1	0	0	0	0	3.79
DUF1357	2	2	0	1	0	0	0	0	1	3.79
Neisseria_PilC	2	2	0	0	1	1	0	0	0	3.79
YHS	2	2	0	0	0	1	0	0	1	3.79
Myotoxins	2	2	0	0	1	0	0	1	0	3.79
Med7	5	5	1	2	2	3	1	1	3	3.77
EBP50_C	6	3	2	1	0	2	2	0	2	3.65
DUF6338	9	8	2	1	3	4	6	2	2	3.64
Bowman-Birk_leg	14	10	1	0	2	2	7	0	6	3.61
DUF3944	3	3	0	0	0	1	0	0	2	3.53

DUF2505	3	3	0	0	0	0	0	1	2	3.53
TetR_C_33	6	1	0	0	0	0	0	0	0	3.50
DUF6414	5	2	0	0	0	0	0	0	0	3.50
WND	5	2	0	0	0	0	0	0	0	3.50
Baculo_11_kDa	14	11	5	5	5	5	3	6	10	3.48
DREV	16	14	7	2	7	5	5	4	11	3.46
Conotoxin	7	6	4	3	3	0	3	2	2	3.46
DUF2615	9	5	0	1	4	2	3	2	4	3.40
NADH-u_ox-rdase	5	4	2	1	2	1	1	0	3	3.40
ThiD2	8	8	1	0	0	1	1	0	6	3.39
GA-like	3	3	2	0	1	1	1	0	1	3.35
Neurokinin_B	3	3	1	0	2	1	1	0	1	3.35
DUF4230	3	3	0	0	0	0	2	1	1	3.33
AmoC	3	3	0	0	1	0	1	0	2	3.33
Methyltransf_2	126	94	28	39	63	36	50	26	83	3.33
RskA	6	5	3	2	0	3	1	0	0	3.30
TetR_C_14	3	3	2	1	1	0	0	0	1	3.27
BaffR-Tall_bind	3	3	1	1	0	1	0	0	2	3.27
HutP	3	3	1	0	1	1	0	2	0	3.27
AHSA1	3	3	2	2	2	2	2	1	2	3.27
YSIRK_signal	3	3	0	1	1	2	0	0	1	3.27
EB	9	14	1	6	6	4	7	2	1	3.24
CTD12	4	3	0	0	0	0	2	0	2	3.24
UBD	7	5	2	4	2	2	2	4	4	3.18
SAM_LFY	4	4	2	2	3	3	2	2	3	3.18
CarS-like	3	3	1	1	1	2	2	1	2	3.18
Type_III_SycN	2	2	0	1	0	0	0	1	1	3.18
Tsi1	2	2	1	1	0	0	1	0	0	3.18
GdpP_PAS	2	2	0	1	0	1	0	0	1	3.18
DUF6806	2	2	1	0	0	1	0	0	1	3.18
DUF6332	2	2	1	0	1	0	0	1	0	3.18
DUF5963	2	2	0	1	1	0	1	0	0	3.18
Cucumopine_C	2	2	0	1	0	0	1	0	1	3.18
KH_9	2	2	0	0	1	1	0	0	1	3.18
SRRM_C	2	2	0	0	1	1	1	0	0	3.18

Methyltrans_Mon_2nd	2	2	0	0	1	0	0	1	1	3.18
DUF3925	2	2	0	0	1	0	1	0	1	3.18
DUF2002	2	2	0	0	1	0	1	0	1	3.18
Hema_stalk	2	2	1	0	1	0	0	0	1	3.18
YbgT_YccB	2	2	0	1	1	0	0	1	0	3.18
Pox_D5	2	2	1	1	0	0	0	1	0	3.18
Orexin	2	2	0	0	1	1	0	0	1	3.18
Gpr1_Fun34_YaaH	2	2	1	0	0	1	0	0	1	3.18
PAX	2	2	0	1	1	0	0	1	0	3.18
Androgen_recep	14	12	10	7	5	8	6	9	9	3.17
DUF6277	3	4	1	2	0	0	1	0	2	3.17
YtkA	3	4	2	0	1	1	0	2	0	3.17
Peptidase_S49_N	15	14	4	8	4	6	4	2	11	3.16
DUF455	4	5	2	3	2	2	3	1	3	3.16
TBD	4	3	0	2	0	0	0	1	2	3.16

Supplementary Table S17. Copy number statistics of *GOT2*, *TAT*, and *CNMT* in the *Stephania longa* and its related species.

**Genes name**	***Stephania longa***	***Stephania japonica***	***Coptis chinensis***	***Aquilegia coerulea***	**Map ID**	**Pathway**
GOT2	slo.H128701 slo.H133035	-	-	-	map00330	Arginine and proline metabolism
					map00220	Arginine biosynthesis
					map00250	Alanine, aspartate and glutamate metabolism
					map00950	Isoquinoline alkaloid biosynthesis
					map00350	Tyrosine metabolism

					map00400	Phenylalanine, tyrosine and tryptophan biosynthesis
					map00950	Isoquinoline alkaloid biosynthesis
					map00360	Phenylalanine metabolism
TAT	slo.H127116	Sjap.Chr11G00258210.1	-	-	map00350	Tyrosine metabolism
					map00400	Phenylalanine, tyrosine and tryptophan biosynthesis
					map00960	Tropane, piperidine and pyridine alkaloid biosynthesis
CNMT	slo.H101991<br>slo.H101993<br>slo.H102463<br>slo.H102585<br>slo.H102590<br>slo.H102591<br>slo.H102599<br>slo.H102601<br>slo.H102604<br>slo.H102605<br>slo.H102612<br>slo.H126519<br>slo.H126524	Sjap.Chr1G00024460.1<br>Sjap.Chr4G00117960.1<br>Sjap.Chr4G00118080.1<br>Sjap.Chr4G00117840.1<br>Sjap.Chr4G00116800.1<br>Sjap.Chr4G00117850.1<br>Sjap.Chr4G00117970.1<br>Sjap.Chr4G00117810.1<br>Sjap.Chr4G00117890.1<br>Sjap.Chr4G00117860.1<br>Sjap.Chr4G00113470.1<br>Sjap.Chr9G00228860.1<br>Sjap.Chr9G00228880.1	cch.078623<br>cch.079412<br>cch.080281<br>cch.081321<br>cch.081485<br>cch.082919<br>cch.083410<br>cch.083473	aco.7G155100<br>aco.7G156200<br>aco.7G041300<br>aco.7G412800<br>aco.7G423900<br>aco.7G424100	map00950<br>map01110	Isoquinoline alkaloid biosynthesis<br>Biosynthesis of secondary metabolites

slo.H126526	Sjap.Chr11G00259430.1
slo.H126530	
slo.H126538	
slo.H126953	

Supplementary Table S18. KEGG pathway analysis of different metabolites in root and stem.

Kegg_pathway	ko_ID	Sig_compound	compound
alpha-Linolenic acid metabolism	ko00592	6	10
Nicotinate and nicotinamide metabolism	ko00760	6	10
Metabolic pathways	ko01100	121	220
Isoquinoline alkaloid biosynthesis	ko00950	11	14
Biosynthesis of secondary metabolites	ko01110	63	106
Glycolysis / Gluconeogenesis	ko00010	1	6
Pentose phosphate pathway	ko00030	4	8
Galactose metabolism	ko00052	9	18
Starch and sucrose metabolism	ko00500	4	8
Amino sugar and nucleotide sugar metabolism	ko00520	5	11
Neomycin, kanamycin and gentamicin biosynthesis	ko00524	3	4
Indole alkaloid biosynthesis	ko00901	2	3
ABC transporters	ko02010	26	38
Phenylalanine metabolism	ko00360	9	18
Phenylalanine, tyrosine and tryptophan biosynthesis	ko00400	5	8
Cyanoamino acid metabolism	ko00460	5	6
Phenylpropanoid biosynthesis	ko00940	11	16
Tropane, piperidine and pyridine alkaloid biosynthesis	ko00960	5	7
Glucosinolate biosynthesis	ko00966	7	7
Aminoacyl-tRNA biosynthesis	ko00970	13	15
Biosynthesis of various secondary metabolites - part 2	ko00998	4	11
2-Oxocarboxylic acid metabolism	ko01210	12	19
Biosynthesis of amino acids	ko01230	21	37
Purine metabolism	ko00230	9	19
Plant hormone signal transduction	ko04075	3	4
Valine, leucine and isoleucine degradation	ko00280	5	5
Valine, leucine and isoleucine biosynthesis	ko00290	6	8
Flavonoid biosynthesis	ko00941	7	7
Pyrimidine metabolism	ko00240	10	15
Propanoate metabolism	ko00640	3	4

Citrate cycle (TCA cycle)	ko00020	1	8
Oxidative phosphorylation	ko00190	1	5
Alanine, aspartate and glutamate metabolism	ko00250	4	10
Lysine degradation	ko00310	8	13
Tyrosine metabolism	ko00350	6	11
Pyruvate metabolism	ko00620	4	7
Glyoxylate and dicarboxylate metabolism	ko00630	7	13
Butanoate metabolism	ko00650	2	7
Sulfur metabolism	ko00920	3	5
Carbon metabolism	ko01200	8	21
Arginine and proline metabolism	ko00330	8	13
Glycerophospholipid metabolism	ko00564	2	6
Ether lipid metabolism	ko00565	1	1
Arginine biosynthesis	ko00220	4	8
Fatty acid degradation	ko00071	1	3
Glycine, serine and threonine metabolism	ko00260	6	11
Tryptophan metabolism	ko00380	2	4
Biosynthesis of cofactors	ko01240	16	34
Flavone and flavonol biosynthesis	ko00944	7	12
Caffeine metabolism	ko00232	1	2
Linoleic acid metabolism	ko00591	9	16
Penicillin and cephalosporin biosynthesis	ko00311	1	1
Pantothenate and CoA biosynthesis	ko00770	4	8
Sphingolipid metabolism	ko00600	2	3
Ubiquinone and other terpenoid-quinone biosynthesis	ko00130	1	3
Monobactam biosynthesis	ko00261	5	6
Thiamine metabolism	ko00730	2	3
Betalain biosynthesis	ko00965	3	3
Glutathione metabolism	ko00480	3	5
Stilbenoid, diarylheptanoid and gingerol biosynthesis	ko00945	3	3
C5-Branched dibasic acid metabolism	ko00660	1	8
Lysine biosynthesis	ko00300	4	7
Biotin metabolism	ko00780	1	1
beta-Alanine metabolism	ko00410	6	10
Ascorbate and aldarate metabolism	ko00053	4	12
Inositol phosphate metabolism	ko00562	1	4
Phosphatidylinositol signaling system	ko04070	1	1
Histidine metabolism	ko00340	2	5
Fatty acid biosynthesis	ko00061	1	5
Fatty acid metabolism	ko01212	1	2
Carotenoid biosynthesis	ko00906	1	1
Vitamin B6 metabolism	ko00750	2	5
Nitrogen metabolism	ko00910	1	1
Isoflavonoid biosynthesis	ko00943	3	3

Cysteine and methionine metabolism	ko00270	7	11
Fructose and mannose metabolism	ko00051	4	6
Pentose and glucuronate interconversions	ko00040	3	9
Glycerolipid metabolism	ko00561	2	4
Zeatin biosynthesis	ko00908	3	10
Carbon fixation in photosynthetic organisms	ko00710	4	9
D-Arginine and D-ornithine metabolism	ko00472	1	1
Photosynthesis	ko00195	1	3
Glycosylphosphatidylinositol (GPI)-anchor biosynthesis	ko00563	1	1
Biosynthesis of unsaturated fatty acids	ko01040	2	8
Sulfur relay system	ko04122	2	3
Porphyrin and chlorophyll metabolism	ko00860	1	1
Carbapenem biosynthesis	ko00332	1	2
Phosphonate and phosphinate metabolism	ko00440	1	2

Note: Kegg_pathway: The name of the pathway; ko_ID: The KO number of the pathway in the KEGG database; Sig_compound: The number of significantly annotated metabolites in that pathway according to KEGG; compound: The number of detected metabolites that belong to that pathway."

Supplementary Table S19. KEGG pathway analysis of different metabolites in root and leaf.

**Kegg_pathway**	**ko_ID**	**Sig_compound**	**compound**
alpha-Linolenic acid metabolism	ko00592	7	10
Isoquinoline alkaloid biosynthesis	ko00950	7	13
Biosynthesis of secondary metabolites	ko01110	66	107
Butanoate metabolism	ko00650	6	7
Metabolic pathways	ko01100	140	222
Glycolysis / Gluconeogenesis	ko00010	3	6
Pentose phosphate pathway	ko00030	7	9
Galactose metabolism	ko00052	12	18
Starch and sucrose metabolism	ko00500	2	8
Amino sugar and nucleotide sugar metabolism	ko00520	6	11
Neomycin, kanamycin and gentamicin biosynthesis	ko00524	2	4
Indole alkaloid biosynthesis	ko00901	3	3
ABC transporters	ko02010	31	38
Arginine and proline metabolism	ko00330	7	13
Carbapenem biosynthesis	ko00332	2	2
Aminoacyl-tRNA biosynthesis	ko00970	14	15
Biosynthesis of amino acids	ko01230	25	36
Phenylalanine metabolism	ko00360	12	18
Phenylalanine, tyrosine and tryptophan biosynthesis	ko00400	4	8
Cyanoamino acid metabolism	ko00460	5	6
Phenylpropanoid biosynthesis	ko00940	11	17
Tropane, piperidine and pyridine alkaloid biosynthesis	ko00960	4	7
Glucosinolate biosynthesis	ko00966	7	7

Biosynthesis of various secondary metabolites - part 2	ko00998	4	11
2-Oxocarboxylic acid metabolism	ko01210	15	19
Ascorbate and aldarate metabolism	ko00053	7	13
Plant hormone signal transduction	ko04075	4	4
Valine, leucine and isoleucine degradation	ko00280	5	5
Valine, leucine and isoleucine biosynthesis	ko00290	7	8
Flavonoid biosynthesis	ko00941	8	9
Pyrimidine metabolism	ko00240	12	15
Propanoate metabolism	ko00640	4	4
Citrate cycle (TCA cycle)	ko00020	4	8
Oxidative phosphorylation	ko00190	3	5
Alanine, aspartate and glutamate metabolism	ko00250	6	10
Lysine degradation	ko00310	6	12
Tyrosine metabolism	ko00350	6	11
Pyruvate metabolism	ko00620	5	7
Glyoxylate and dicarboxylate metabolism	ko00630	8	13
Nicotinate and nicotinamide metabolism	ko00760	8	10
Sulfur metabolism	ko00920	4	5
Carbon metabolism	ko01200	14	22
Glycerophospholipid metabolism	ko00564	3	6
Ether lipid metabolism	ko00565	1	1
Arginine biosynthesis	ko00220	7	8
Fatty acid degradation	ko00071	1	3
Glycine, serine and threonine metabolism	ko00260	7	11
Tryptophan metabolism	ko00380	5	5
Biosynthesis of cofactors	ko01240	22	35
Carbon fixation in photosynthetic organisms	ko00710	5	9
Flavone and flavonol biosynthesis	ko00944	9	11
Purine metabolism	ko00230	14	20
Caffeine metabolism	ko00232	1	2
Linoleic acid metabolism	ko00591	13	17
Penicillin and cephalosporin biosynthesis	ko00311	1	1
Pantothenate and CoA biosynthesis	ko00770	7	8
Sphingolipid metabolism	ko00600	2	3
Ubiquinone and other terpenoid-quinone biosynthesis	ko00130	2	3
Monobactam biosynthesis	ko00261	5	6
Thiamine metabolism	ko00730	3	3
Betalain biosynthesis	ko00965	1	3
Stilbenoid, diarylheptanoid and gingerol biosynthesis	ko00945	3	3
C5-Branched dibasic acid metabolism	ko00660	4	8
Pentose and glucuronate interconversions	ko00040	4	9
Lysine biosynthesis	ko00300	4	6
Histidine metabolism	ko00340	4	5
Taurine and hypotaurine metabolism	ko00430	1	1

Fructose and mannose metabolism	ko00051	4	5
Glycerolipid metabolism	ko00561	2	4
Inositol phosphate metabolism	ko00562	3	4
Biotin metabolism	ko00780	1	1
beta-Alanine metabolism	ko00410	8	10
Phosphatidylinositol signaling system	ko04070	1	1
Fatty acid biosynthesis	ko00061	1	5
Fatty acid metabolism	ko01212	1	2
Carotenoid biosynthesis	ko00906	1	1
Vitamin B6 metabolism	ko00750	3	5
Nitrogen metabolism	ko00910	1	1
Isoflavonoid biosynthesis	ko00943	2	3
Cysteine and methionine metabolism	ko00270	7	11
Cutin, suberine and wax biosynthesis	ko00073	1	2
Zeatin biosynthesis	ko00908	5	10
D-Arginine and D-ornithine metabolism	ko00472	1	1
Photosynthesis	ko00195	1	3
Glutathione metabolism	ko00480	2	5
Glycosylphosphatidylinositol (GPI)-anchor biosynthesis	ko00563	1	1
Biosynthesis of unsaturated fatty acids	ko01040	2	8
Folate biosynthesis	ko00790	1	1
Sulfur relay system	ko04122	3	3
Porphyrin and chlorophyll metabolism	ko00860	1	1
Phosphonate and phosphinate metabolism	ko00440	1	2

Supplementary Table S20. KEGG pathway analysis of different metabolites in leaf and stem.

Kegg_pathway	ko_ID	Sig_compound	compound
alpha-Linolenic acid metabolism	ko00592	4	9
Butanoate metabolism	ko00650	3	6
Metabolic pathways	ko01100	88	214
Phenylalanine metabolism	ko00360	8	14
Phenylalanine, tyrosine and tryptophan biosynthesis	ko00400	4	8
Cyanoamino acid metabolism	ko00460	4	6
Phenylpropanoid biosynthesis	ko00940	9	17
Tropane, piperidine and pyridine alkaloid biosynthesis	ko00960	3	6
Glucosinolate biosynthesis	ko00966	2	7
Aminoacyl-tRNA biosynthesis	ko00970	6	15
Biosynthesis of various secondary metabolites - part 2	ko00998	3	11
Biosynthesis of secondary metabolites	ko01110	53	106
2-Oxocarboxylic acid metabolism	ko01210	7	19
Biosynthesis of amino acids	ko01230	18	36
ABC transporters	ko02010	12	38

Ascorbate and aldarate metabolism	ko00053	4	13
Plant hormone signal transduction	ko04075	3	4
Arginine biosynthesis	ko00220	5	8
Fatty acid degradation	ko00071	1	3
Lysine degradation	ko00310	6	12
Citrate cycle (TCA cycle)	ko00020	3	7
Pyruvate metabolism	ko00620	5	6
Glyoxylate and dicarboxylate metabolism	ko00630	5	12
Carbon fixation in photosynthetic organisms	ko00710	5	8
Carbon metabolism	ko01200	10	19
Flavone and flavonol biosynthesis	ko00944	10	12
Purine metabolism	ko00230	8	19
Caffeine metabolism	ko00232	1	2
Valine, leucine and isoleucine degradation	ko00280	1	4
Valine, leucine and isoleucine biosynthesis	ko00290	4	8
Penicillin and cephalosporin biosynthesis	ko00311	1	1
Pantothenate and CoA biosynthesis	ko00770	7	8
Biosynthesis of cofactors	ko01240	10	34
Glutathione metabolism	ko00480	2	5
Flavonoid biosynthesis	ko00941	5	8
Stilbenoid, diarylheptanoid and gingerol biosynthesis	ko00945	2	3
Linoleic acid metabolism	ko00591	11	16
Isoquinoline alkaloid biosynthesis	ko00950	9	14
Tryptophan metabolism	ko00380	3	5
Indole alkaloid biosynthesis	ko00901	1	3
Arginine and proline metabolism	ko00330	2	13
Glycolysis / Gluconeogenesis	ko00010	2	6
Pentose and glucuronate interconversions	ko00040	2	8
Fructose and mannose metabolism	ko00051	3	6
Galactose metabolism	ko00052	3	18
Glycerolipid metabolism	ko00561	1	4
Inositol phosphate metabolism	ko00562	1	4
Glycerophospholipid metabolism	ko00564	3	6
Propanoate metabolism	ko00640	1	1
Nicotinate and nicotinamide metabolism	ko00760	3	9
Fatty acid biosynthesis	ko00061	1	5
Pyrimidine metabolism	ko00240	5	13
beta-Alanine metabolism	ko00410	6	9
Fatty acid metabolism	ko01212	1	2
Carotenoid biosynthesis	ko00906	1	1
Pentose phosphate pathway	ko00030	3	8
Isoflavonoid biosynthesis	ko00943	1	3
Phosphonate and phosphinate metabolism	ko00440	1	1
Glycine, serine and threonine metabolism	ko00260	6	11

Oxidative phosphorylation	ko00190	1	4
Alanine, aspartate and glutamate metabolism	ko00250	2	9
Tyrosine metabolism	ko00350	3	10
Monobactam biosynthesis	ko00261	4	6
Cysteine and methionine metabolism	ko00270	4	11
Lysine biosynthesis	ko00300	5	7
Histidine metabolism	ko00340	2	5
Cutin, suberine and wax biosynthesis	ko00073	1	2
D-Arginine and D-ornithine metabolism	ko00472	1	1
Glycosylphosphatidylinositol (GPI)-anchor biosynthesis	ko00563	1	1
Sphingolipid metabolism	ko00600	2	3
Biosynthesis of unsaturated fatty acids	ko01040	2	8
Ubiquinone and other terpenoid-quinone biosynthesis	ko00130	1	3
Folate biosynthesis	ko00790	1	1
Sulfur metabolism	ko00920	3	4
C5-Branched dibasic acid metabolism	ko00660	1	8
Porphyrin and chlorophyll metabolism	ko00860	1	1
Carbapenem biosynthesis	ko00332	1	2
Zeatin biosynthesis	ko00908	2	10
Starch and sucrose metabolism	ko00500	1	8
Betalain biosynthesis	ko00965	1	3
Sulfur relay system	ko04122	1	3

Supplementary Table S21. KEGG pathway analysis of different metabolites in fruit and stem.

**Kegg_pathway**	**ko_ID**	**Sig_compound**	**compound**
Isoquinoline alkaloid biosynthesis	ko00950	12	13
Biosynthesis of secondary metabolites	ko01110	63	109
Glycerophospholipid metabolism	ko00564	2	6
Metabolic pathways	ko01100	115	219
Phenylpropanoid biosynthesis	ko00940	11	16
Purine metabolism	ko00230	11	20
ABC transporters	ko02010	15	38
Flavonoid biosynthesis	ko00941	10	11
Phenylalanine metabolism	ko00360	12	17
Alanine, aspartate and glutamate metabolism	ko00250	6	10
Pyrimidine metabolism	ko00240	6	14
Valine, leucine and isoleucine degradation	ko00280	2	5
Propanoate metabolism	ko00640	3	3
Citrate cycle (TCA cycle)	ko00020	5	8
Oxidative phosphorylation	ko00190	3	5
Lysine degradation	ko00310	9	13
Tyrosine metabolism	ko00350	6	10
Pyruvate metabolism	ko00620	4	7

Glyoxylate and dicarboxylate metabolism	ko00630	5	12
Butanoate metabolism	ko00650	3	7
Nicotinate and nicotinamide metabolism	ko00760	3	10
Sulfur metabolism	ko00920	4	5
Carbon metabolism	ko01200	9	20
Arginine and proline metabolism	ko00330	3	13
2-Oxocarboxylic acid metabolism	ko01210	12	19
Biosynthesis of amino acids	ko01230	21	37
Biosynthesis of cofactors	ko01240	17	34
Fatty acid degradation	ko00071	1	3
Glycine, serine and threonine metabolism	ko00260	5	11
Tryptophan metabolism	ko00380	3	5
Phenylalanine, tyrosine and tryptophan biosynthesis	ko00400	4	8
Indole alkaloid biosynthesis	ko00901	2	3
Glucosinolate biosynthesis	ko00966	2	7
Aminoacyl-tRNA biosynthesis	ko00970	4	15
Biosynthesis of various secondary metabolites - part 2	ko00998	6	11
alpha-Linolenic acid metabolism	ko00592	4	9
Flavone and flavonol biosynthesis	ko00944	8	12
Caffeine metabolism	ko00232	1	2
Valine, leucine and isoleucine biosynthesis	ko00290	5	8
Linoleic acid metabolism	ko00591	12	16
Ascorbate and aldarate metabolism	ko00053	6	12
Glutathione metabolism	ko00480	1	5
Plant hormone signal transduction	ko04075	2	4
C5-Branched dibasic acid metabolism	ko00660	6	8
Pentose and glucuronate interconversions	ko00040	5	9
Arginine biosynthesis	ko00220	4	8
Lysine biosynthesis	ko00300	5	7
Histidine metabolism	ko00340	2	5
Taurine and hypotaurine metabolism	ko00430	1	1
Glycolysis / Gluconeogenesis	ko00010	2	6
Fructose and mannose metabolism	ko00051	3	6
Galactose metabolism	ko00052	4	18
Glycerolipid metabolism	ko00561	1	4
Inositol phosphate metabolism	ko00562	2	4
Carbon fixation in photosynthetic organisms	ko00710	4	9
Stilbenoid, diarylheptanoid and gingerol biosynthesis	ko00945	2	3
Tropane, piperidine and pyridine alkaloid biosynthesis	ko00960	3	7
Isoflavonoid biosynthesis	ko00943	4	4
Cysteine and methionine metabolism	ko00270	6	11
Betalain biosynthesis	ko00965	1	2
Monobactam biosynthesis	ko00261	2	6
beta-Alanine metabolism	ko00410	3	9

Cyanoamino acid metabolism	ko00460	1	6
Pantothenate and CoA biosynthesis	ko00770	3	8
Cutin, suberine and wax biosynthesis	ko00073	1	2
Zeatin biosynthesis	ko00908	4	10
Amino sugar and nucleotide sugar metabolism	ko00520	3	11
Pentose phosphate pathway	ko00030	3	8
Vitamin B6 metabolism	ko00750	2	5
Neomycin, kanamycin and gentamicin biosynthesis	ko00524	1	4
Biosynthesis of unsaturated fatty acids	ko01040	2	8
Ubiquinone and other terpenoid-quinone biosynthesis	ko00130	2	3
Folate biosynthesis	ko00790	1	1
Photosynthesis	ko00195	2	3
Fatty acid biosynthesis	ko00061	1	5
Porphyrin and chlorophyll metabolism	ko00860	1	1
Starch and sucrose metabolism	ko00500	2	8
Monoterpenoid biosynthesis	ko00902	2	2
Phosphonate and phosphinate metabolism	ko00440	1	2
Sulfur relay system	ko04122	1	3

Supplementary Table S22. KEGG pathway analysis of different metabolites in fruit and root.

Kegg_pathway	ko_ID	Sig_compound	compound
alpha-Linolenic acid metabolism	ko00592	7	10
Nicotinate and nicotinamide metabolism	ko00760	8	10
Metabolic pathways	ko01100	150	223
Isoquinoline alkaloid biosynthesis	ko00950	13	14
Biosynthesis of secondary metabolites	ko01110	75	109
Glycolysis / Gluconeogenesis	ko00010	2	6
Pentose phosphate pathway	ko00030	5	8
Galactose metabolism	ko00052	12	18
Starch and sucrose metabolism	ko00500	4	8
Amino sugar and nucleotide sugar metabolism	ko00520	7	11
Neomycin, kanamycin and gentamicin biosynthesis	ko00524	2	4
Indole alkaloid biosynthesis	ko00901	3	3
ABC transporters	ko02010	29	38
Purine metabolism	ko00230	14	20
Glycerophospholipid metabolism	ko00564	3	6
Phenylalanine metabolism	ko00360	15	18
Phenylalanine, tyrosine and tryptophan biosynthesis	ko00400	5	8
Cyanoamino acid metabolism	ko00460	5	6
Phenylpropanoid biosynthesis	ko00940	12	16
Tropane, piperidine and pyridine alkaloid biosynthesis	ko00960	5	7
Glucosinolate biosynthesis	ko00966	7	7

Aminoacyl-tRNA biosynthesis	ko00970	12	15
Biosynthesis of various secondary metabolites - part 2	ko00998	7	11
2-Oxocarboxylic acid metabolism	ko01210	16	19
Biosynthesis of amino acids	ko01230	26	37
Plant hormone signal transduction	ko04075	4	4
Valine, leucine and isoleucine degradation	ko00280	4	5
Valine, leucine and isoleucine biosynthesis	ko00290	4	8
Flavonoid biosynthesis	ko00941	10	11
Alanine, aspartate and glutamate metabolism	ko00250	8	10
Arginine and proline metabolism	ko00330	8	13
Arginine biosynthesis	ko00220	7	8
Citrate cycle (TCA cycle)	ko00020	5	8
Glyoxylate and dicarboxylate metabolism	ko00630	10	13
Carbon metabolism	ko01200	13	21
Biosynthesis of cofactors	ko01240	22	34
Glycine, serine and threonine metabolism	ko00260	4	11
Tryptophan metabolism	ko00380	4	5
Pyruvate metabolism	ko00620	1	7
Carbon fixation in photosynthetic organisms	ko00710	5	9
Caffeine metabolism	ko00232	1	2
Linoleic acid metabolism	ko00591	9	17
Penicillin and cephalosporin biosynthesis	ko00311	1	1
Pantothenate and CoA biosynthesis	ko00770	7	8
Ascorbate and aldarate metabolism	ko00053	8	12
Sphingolipid metabolism	ko00600	2	3
Ubiquinone and other terpenoid-quinone biosynthesis	ko00130	3	3
Monobactam biosynthesis	ko00261	3	6
Tyrosine metabolism	ko00350	6	11
Thiamine metabolism	ko00730	2	3
Betalain biosynthesis	ko00965	3	3
Glutathione metabolism	ko00480	3	5
Stilbenoid, diarylheptanoid and gingerol biosynthesis	ko00945	2	3
C5-Branched dibasic acid metabolism	ko00660	6	8
Pentose and glucuronate interconversions	ko00040	4	9
Lysine biosynthesis	ko00300	5	7
Lysine degradation	ko00310	8	13
Histidine metabolism	ko00340	3	5
Taurine and hypotaurine metabolism	ko00430	1	1
Butanoate metabolism	ko00650	4	7
Fructose and mannose metabolism	ko00051	5	6
Glycerolipid metabolism	ko00561	1	4
Inositol phosphate metabolism	ko00562	3	4
Propanoate metabolism	ko00640	2	4
Biotin metabolism	ko00780	1	1

beta-Alanine metabolism	ko00410	9	10
Phosphatidylinositol signaling system	ko04070	1	1
Flavone and flavonol biosynthesis	ko00944	4	10
Fatty acid biosynthesis	ko00061	2	5
Pyrimidine metabolism	ko00240	11	15
Fatty acid metabolism	ko01212	1	2
Carotenoid biosynthesis	ko00906	1	1
Vitamin B6 metabolism	ko00750	4	5
Nitrogen metabolism	ko00910	1	1
Zeatin biosynthesis	ko00908	4	10
Cysteine and methionine metabolism	ko00270	5	10
Cutin, suberine and wax biosynthesis	ko00073	1	2
D-Arginine and D-ornithine metabolism	ko00472	1	1
Photosynthesis	ko00195	1	3
Oxidative phosphorylation	ko00190	1	5
Glycosylphosphatidylinositol (GPI)-anchor biosynthesis	ko00563	1	1
Folate biosynthesis	ko00790	1	1
Isoflavonoid biosynthesis	ko00943	3	3
Carbapenem biosynthesis	ko00332	1	2
Monoterpenoid biosynthesis	ko00902	2	2
Phosphonate and phosphinate metabolism	ko00440	1	2
Sulfur metabolism	ko00920	1	4
Sulfur relay system	ko04122	1	3

Supplementary Table S23. KEGG pathway analysis of different metabolites in fruit and leaf.

Kegg_pathway	ko_ID	Sig_compound	compound
alpha-Linolenic acid metabolism	ko00592	6	9
Isoquinoline alkaloid biosynthesis	ko00950	10	14
Biosynthesis of secondary metabolites	ko01110	61	110
Butanoate metabolism	ko00650	5	7
Metabolic pathways	ko01100	117	222
Purine metabolism	ko00230	8	19
Glycerophospholipid metabolism	ko00564	3	6
Phenylalanine metabolism	ko00360	14	17
Phenylalanine, tyrosine and tryptophan biosynthesis	ko00400	5	8
Cyanoamino acid metabolism	ko00460	1	6
Phenylpropanoid biosynthesis	ko00940	14	17
Tropane, piperidine and pyridine alkaloid biosynthesis	ko00960	2	7
Glucosinolate biosynthesis	ko00966	1	7
Aminoacyl-tRNA biosynthesis	ko00970	3	15
Biosynthesis of various secondary metabolites - part 2	ko00998	5	11
2-Oxocarboxylic acid metabolism	ko01210	11	19
Biosynthesis of amino acids	ko01230	19	37

ABC transporters	ko02010	12	38
Ascorbate and aldarate metabolism	ko00053	11	13
Plant hormone signal transduction	ko04075	3	4
Flavonoid biosynthesis	ko00941	10	12
Alanine, aspartate and glutamate metabolism	ko00250	6	10
Pyrimidine metabolism	ko00240	8	14
Valine, leucine and isoleucine degradation	ko00280	2	5
Propanoate metabolism	ko00640	3	3
Citrate cycle (TCA cycle)	ko00020	7	8
Oxidative phosphorylation	ko00190	3	5
Lysine degradation	ko00310	8	13
Tyrosine metabolism	ko00350	6	11
Pyruvate metabolism	ko00620	5	7
Glyoxylate and dicarboxylate metabolism	ko00630	9	13
Nicotinate and nicotinamide metabolism	ko00760	3	10
Sulfur metabolism	ko00920	3	5
Carbon metabolism	ko01200	12	21
Arginine biosynthesis	ko00220	6	8
Biosynthesis of cofactors	ko01240	13	35
Fatty acid degradation	ko00071	2	3
Carbon fixation in photosynthetic organisms	ko00710	5	9
Flavone and flavonol biosynthesis	ko00944	8	11
Valine, leucine and isoleucine biosynthesis	ko00290	5	8
Linoleic acid metabolism	ko00591	12	16
Fatty acid biosynthesis	ko00061	2	5
Fatty acid elongation	ko00062	1	1
Cutin, suberine and wax biosynthesis	ko00073	1	2
Biosynthesis of unsaturated fatty acids	ko01040	3	8
Fatty acid metabolism	ko01212	2	2
Glutathione metabolism	ko00480	1	5
Stilbenoid, diarylheptanoid and gingerol biosynthesis	ko00945	2	3
C5-Branched dibasic acid metabolism	ko00660	5	8
Arginine and proline metabolism	ko00330	3	13
Pentose and glucuronate interconversions	ko00040	7	9
Lysine biosynthesis	ko00300	3	7
Histidine metabolism	ko00340	2	5
Taurine and hypotaurine metabolism	ko00430	1	1
Glycolysis / Gluconeogenesis	ko00010	2	6
Fructose and mannose metabolism	ko00051	2	6
Galactose metabolism	ko00052	5	18
Glycerolipid metabolism	ko00561	1	4
Inositol phosphate metabolism	ko00562	2	4
beta-Alanine metabolism	ko00410	3	9
Carotenoid biosynthesis	ko00906	1	1

Pentose phosphate pathway	ko00030	6	9
Isoflavonoid biosynthesis	ko00943	3	4
Betalain biosynthesis	ko00965	2	3
Tryptophan metabolism	ko00380	1	5
Cysteine and methionine metabolism	ko00270	4	11
Zeatin biosynthesis	ko00908	3	10
Monobactam biosynthesis	ko00261	2	6
D-Arginine and D-ornithine metabolism	ko00472	1	1
Amino sugar and nucleotide sugar metabolism	ko00520	2	11
Vitamin B6 metabolism	ko00750	3	5
Glycosylphosphatidylinositol (GPI)-anchor biosynthesis	ko00563	1	1
Sphingolipid metabolism	ko00600	1	3
Pantothenate and CoA biosynthesis	ko00770	2	8
Photosynthesis	ko00195	1	3
Glycine, serine and threonine metabolism	ko00260	3	11
Porphyrin and chlorophyll metabolism	ko00860	1	1
Ubiquinone and other terpenoid-quinone biosynthesis	ko00130	1	3
Starch and sucrose metabolism	ko00500	2	8
Monoterpenoid biosynthesis	ko00902	2	2
Phosphonate and phosphinate metabolism	ko00440	1	2

Supplementary Table S24. The average expression quantity of positive selected genes in the genus *Stephania* in four tissues (TPM).

PSG Genes	Genes name	Fruit	Leaf	Root	Stem
slo.H128504	RFNR2	41.61	41.78	65.30	50.07
slo.H119789	ARGAH2	54.12	47.14	74.67	83.47
slo.H113043	AT4G19130	3.03	3.62	10.08	5.53
slo.H109101	AT4G26950	41.19	120.73	145.41	111.77
slo.H117528	AT2G33680	1.50	1.05	3.07	2.25
slo.H127751	AT5G54880	1.78	1.44	4.66	2.15
slo.H118435	MOS1	57.30	82.36	81.52	89.31
slo.H109211	DEG14	0.85	1.95	5.93	3.38
slo.H118232	AT1G70150	0.34	1.59	1.41	1.70
slo.H114290	GDAP1	18.73	18.82	24.95	24.79
slo.H118265	AT3G01380	8.47	12.87	18.26	14.14
slo.H112969	TRM9	4.56	7.01	10.92	5.09
slo.H120898	AT2G05830	14.76	67.91	47.08	86.26
slo.H121952	TAF15b	75.17	77.02	72.97	59.09
slo.H103374	EMB2423	22.40	36.97	32.05	30.21
slo.H102991	ACS	47.01	141.64	107.06	102.54
slo.H115387	sks3	1.04	0.55	4.10	2.04
slo.H116104	DRH1	11.08	30.49	37.74	22.93
slo.H106752	TTN7	2.23	1.69	17.15	2.01

slo.H126185	AT5G63680	10.06	6.97	61.89	26.83
slo.H118137	AT2G02590	2.26	1.86	2.80	2.47
slo.H121840	AT3G56330	2.35	3.02	1.57	1.67
slo.H102458	BGAL12	14.88	18.13	36.42	29.97
slo.H123707	AT2G39580	24.91	26.24	27.16	24.45
slo.H101657	AT4G32060	8.58	1.70	7.15	3.59
slo.H120361	AT3G60370	0.52	4.03	1.91	0.80
slo.H127899	TOPP2	19.56	50.93	60.87	42.32
slo.H116747	AT3G45020	10.17	41.66	50.65	34.90
slo.H109568	AT2G32160	15.10	23.23	45.60	28.61
slo.H103250	FRS3	2.11	2.83	4.36	2.67
slo.H104215	CUL1	10.93	12.30	14.39	9.58
slo.H111978	AT4G19440	16.40	28.82	50.39	28.08
slo.H108370	SRT2	8.92	4.89	8.97	6.87
slo.H115560	AT4G38020	2.31	12.13	17.30	5.74
slo.H124514	FPA	8.18	11.48	13.00	10.01
slo.H102980	AT3G13225	53.18	83.82	136.61	75.41
slo.H122603	CYP704A1	2.10	11.06	12.60	15.66
slo.H121536	AT1G80360	43.21	46.61	56.53	59.28
slo.H112146	GlcNAc1pUT1	4.73	19.58	10.69	10.53
slo.H116438	AT5G37290	3.72	5.82	3.96	4.07
slo.H103684	PEX14	233.20	320.36	241.24	248.52
slo.H126138	OTP86	0.14	0.52	1.23	0.77
slo.H113153	HDG2	8.06	7.17	0.00	1.99
slo.H125213	AT1G20380	4.82	8.69	7.91	7.64
slo.H118419	PDF1B	11.64	21.93	9.67	10.62
slo.H100899	PBRP	11.70	81.77	46.73	47.68
slo.H124176	FACE2	6.67	10.78	13.63	9.77
slo.H120168	AT1G65070	0.91	5.02	4.11	4.24
slo.H127735	UBP10	10.62	24.78	21.66	17.05
slo.H116779	SOS4	38.14	19.51	21.06	11.05
slo.H129666	PAC	12.36	47.70	29.76	34.36
slo.H121774	AT3G14170	1.08	5.65	0.50	2.51
slo.H116428	PUM6	24.45	37.53	59.36	40.78
slo.H122667	AT3G52210	31.61	61.27	84.98	52.27
slo.H125109	AT1G21580	19.33	18.04	24.78	16.60
slo.H100776	AT4G39160	29.03	38.85	40.98	36.98
slo.H100059	CHB3	26.35	44.61	43.43	37.23
slo.H126583	AT1G63080	2.93	8.07	7.59	6.02
slo.H128913	APG7	16.72	18.74	18.06	16.41
slo.H108563	EMB1135	23.87	36.74	51.89	41.67
slo.H127834	AT1G05060	13.61	15.33	16.59	19.34
slo.H102842	AT3G14172	8.11	12.43	14.98	26.06
slo.H119342	AT1G18410	26.42	47.42	49.25	55.47

slo.H101267	AT5G14850	7.77	7.09	11.62	10.00
slo.H128354	UBP15	18.02	18.07	31.40	19.96
slo.H120358	SRS2	3.06	4.54	6.27	5.12
slo.H119238	AT4G01290	60.26	87.10	109.21	80.63
slo.H120576	AT5G58370	4.57	7.04	15.57	7.32
slo.H108495	QRT1	0.08	0.00	1.54	1.62
slo.H108959	NDR1	1.74	10.07	13.30	14.99
slo.H106439	TIC21	48.55	96.00	105.18	93.14
slo.H100313	UBC8	28.99	45.37	56.57	39.49
slo.H103330	POLA2	0.61	0.65	0.66	0.74
slo.H122112	CPL2	11.63	19.56	19.93	17.22
slo.H127933	XYLT	33.46	65.99	107.24	76.66
slo.H119120	TUB8	8.71	7.60	27.08	24.90
slo.H130315	AT5G02860	2.20	11.02	7.17	8.97
slo.H121855	AT5G05310	15.54	12.34	24.20	15.78
slo.H101663	AT2G25280	27.29	25.50	30.08	33.19
slo.H112019	HEXO2	11.66	13.23	12.78	10.12
slo.H124932	AT5G63370	96.92	175.57	196.41	151.52
slo.H105855	AT1G49380	3.21	27.44	17.92	17.95
slo.H133653	AT5G58100	20.81	30.14	43.58	38.96
slo.H109065	XIJ	0.28	0.27	1.08	0.59
slo.H102416	GLDP1	151.51	73.27	126.61	66.97
slo.H121786	AT1G22870	56.30	126.97	144.22	105.94
slo.H116173	AT2G20790	7.49	8.34	9.44	9.15
slo.H108243	ACT1	26.59	105.53	128.85	127.23
slo.H102474	AT3G57060	0.22	1.18	0.66	1.02
slo.H122199	AT3G09080	0.77	1.52	0.70	4.80
slo.H121679	AT1G51610	0.80	1.54	2.14	1.83
slo.H122500	AT1G73170	21.04	51.63	25.86	52.02
slo.H112634	SMG7	14.58	31.85	60.34	39.48
slo.H126059	AT5G16350	42.16	372.79	65.25	116.21
slo.H123591	PRMT6	6.11	9.97	22.05	10.79
slo.H119257	AT4G11350	22.98	6.39	58.33	48.16
slo.H101073	AT4G09760	39.60	42.73	47.23	49.12
slo.H102561	AT5G11980	23.58	28.02	33.81	31.35
slo.H131658	AT4G17540	11.53	18.46	12.05	15.33
slo.H116973	AT5G16610	8.18	29.14	18.85	18.23
slo.H102182	GLDH	4.14	14.62	48.85	52.73
slo.H100985	ATR	14.83	15.86	21.13	21.61
slo.H103763	AT3G26480	21.03	50.74	75.28	60.15
slo.H120825	CID4	67.71	102.83	99.57	94.50
slo.H126724	AT5G14770	1.00	0.80	2.23	1.22
slo.H111155	AT5G50840	24.04	60.75	47.35	44.03
slo.H129284	AT4G31170	17.20	24.44	25.37	21.02

slo.H126701	AT2G19260	14.34	19.09	18.68	17.03
slo.H115986	AT5G02860	5.20	19.48	24.56	15.50
slo.H121074	AT1G06710	17.49	31.71	30.23	25.45
slo.H119776	AT1G08410	81.22	128.18	154.62	104.60
slo.H109381	AT5G55220	5.57	30.35	32.04	30.05
slo.H105688	AT3G19990	59.04	13.19	14.40	8.93
slo.H114001	AT1G01770	8.06	6.76	5.94	5.98
slo.H110255	AT4G23540	9.54	17.38	28.06	22.21
slo.H107040	AT5G15880	20.04	30.51	32.17	37.48
slo.H100379	SBT4.12	14.51	3.64	15.10	39.49
slo.H100108	FC1	45.97	43.07	92.18	136.59
slo.H103700	EX1	36.06	53.38	48.24	41.15
slo.H107883	AT3G07700	5.30	43.81	11.94	16.53
slo.H100837	AT1G67530	26.05	24.56	35.82	24.20
slo.H106554	ATGRIP	65.46	142.14	165.46	147.01
slo.H117029	CPSF73-I	7.96	16.86	20.12	17.96
slo.H102446	emb1688	39.93	66.80	60.65	60.69
slo.H118060	AT3G28720	32.93	27.23	25.90	19.22
slo.H103274	AT3G27180	4.30	17.82	13.77	11.86
slo.H112192	AT5G45660	1.10	1.04	1.11	0.79
slo.H102845	AT5G21280	1.84	3.11	4.49	1.57
slo.H118443	AT1G17230	5.78	2.21	0.36	2.65
slo.H130992	RAD50	25.17	39.46	51.03	54.87
slo.H111349	AT3G02760	21.58	51.99	47.31	28.10
slo.H119530	AT5G51880	46.49	43.38	46.14	54.87
slo.H105435	SDG40	10.18	26.43	15.92	13.68
slo.H119031	AT5G28400	8.44	13.29	17.90	10.32
slo.H105391	AT2G33680	0.60	0.77	1.25	0.79
slo.H110532	AT2G41945	30.56	80.13	99.63	71.42
slo.H120969	TRZ4	7.87	10.36	7.78	7.30
slo.H116655	AT3G62010	30.93	60.76	35.68	40.73
slo.H105622	PARC6	6.45	3.36	9.43	7.10
slo.H127872	AT4G22290	9.68	18.28	44.62	17.51
slo.H113199	AT1G17640	17.59	55.18	37.17	39.58
slo.H126596	RCA	13.08	26.82	13.51	12.97
slo.H102322	AT1G73020	12.05	7.87	8.30	8.80
slo.H109986	AT4G26370	8.49	68.62	49.62	31.96
slo.H116058	AT1G03150	31.21	35.85	65.91	57.88
slo.H106281	AT5G66810	27.95	35.85	24.06	22.31
slo.H116483	MCM3	0.05	0.40	1.90	2.65
slo.H116017	AT5G25040	6.98	12.58	10.30	10.96
slo.H123649	AT2G41640	154.67	132.92	44.98	77.20
slo.H121008	GL2	4.81	5.37	124.25	114.51
slo.H130721	CRT3	45.35	122.80	132.42	72.98

slo.H112974	AT1G31500	8.96	33.50	9.25	24.77
slo.H120980	AT4G32790	66.70	101.99	59.98	35.68
slo.H117972	AT3G55160	5.22	6.42	8.61	8.56
slo.H112048	AT2G45500	54.43	102.01	143.04	134.56
slo.H103677	AT5G26680	31.72	63.88	98.77	66.38
slo.H113652	MED31	11.01	12.28	14.74	17.60
slo.H113111	NOA1	2.35	3.76	6.74	4.59
slo.H112963	AT5G02860	2.48	5.09	11.65	6.62
slo.H108988	AT1G36050	23.77	22.04	37.30	39.16
slo.H120563	S1P	18.48	17.01	27.51	19.51
slo.H127115	AT5G27410	9.41	9.48	37.06	10.08
slo.H124323	AT3G32930	12.17	40.00	29.68	17.43
slo.H121412	OCP3	7.65	20.75	18.33	12.42
slo.H113911	ABCA12	85.06	147.81	161.25	132.11
slo.H113995	KEA2	24.44	41.02	47.73	34.22
slo.H110618	AT2G40980	26.40	69.15	59.44	46.97
slo.H122170	RPL4	13.77	77.47	37.84	37.12
slo.H103528	AT3G57880	115.15	68.83	43.75	49.74
slo.H101387	FBW2	69.16	73.51	125.15	81.70
slo.H130709	PPI1	15.62	33.33	10.24	27.32
slo.H121591	AT3G61750	17.18	11.69	19.93	12.05
slo.H122148	AT5G41330	7.97	20.96	26.69	10.18
slo.H119168	AT2G41600	6.76	5.10	7.60	4.37
slo.H119144	AE7	39.24	36.42	32.50	36.80
slo.H116929	AT1G14810	27.85	25.83	44.16	22.91
slo.H108236	AT3G52155	17.52	37.04	29.42	25.81
slo.H122436	AT1G16800	14.75	28.31	54.26	46.79
slo.H124208	ROC4	11.90	27.73	24.09	26.88
slo.H103719	DWA3	55.86	94.00	118.27	97.96
slo.H103367	AT1G67700	14.03	44.21	15.47	30.93
slo.H102414	AT5G11840	0.61	19.97	0.75	3.24
slo.H125381	AT5G23430	8.35	18.20	16.96	11.42
slo.H125924	PAA2	28.39	29.52	52.58	41.16
slo.H108609	QRT3	3.42	0.15	0.78	1.16
slo.H110499	GLT1	33.95	133.67	99.73	139.20
slo.H101222	UBP15	25.26	43.90	35.83	33.75
slo.H115853	TZP	7.92	18.57	21.04	9.94
slo.H114330	DGD2	15.62	14.39	14.02	13.01
slo.H116394	AT5G55070	90.96	42.12	22.46	21.33
slo.H128396	PTAC12	21.29	33.96	58.47	41.22
slo.H109210	EMB2729	3.38	6.69	19.58	5.04
slo.H109153	AT5G55060	25.54	21.74	57.74	23.12
slo.H130493	AT1G05790	15.43	13.24	18.50	12.59
slo.H105502	ARF2	100.67	23.25	131.52	105.83

slo.H129846	AT5G41120	16.98	57.34	31.78	47.77
slo.H107238	AT5G64160	36.53	55.77	44.72	49.52
slo.H121630	AT5G14770	0.00	0.12	0.60	0.19
slo.H126622	3-Aug	18.64	29.56	46.08	37.47
slo.H111966	TPR9	8.93	20.59	21.12	17.33
slo.H119538	SSE1	6.59	22.72	17.33	14.83
slo.H101660	NUB1	41.69	82.00	38.02	32.77
slo.H110000	AT5G17840	22.68	27.29	12.10	17.03
slo.H103558	AT1G70590	7.04	14.04	21.83	20.08
slo.H117166	CIB5	0.00	0.00	1.63	1.82
slo.H105839	DTX35	4.66	9.15	11.33	7.83
slo.H111284	HCC1	11.81	30.18	26.12	18.55
slo.H129706	RRP4	15.57	24.59	31.55	21.69
slo.H105610	STT3A	41.87	39.27	62.01	51.16
slo.H109526	AT5G55520	0.61	0.32	1.65	1.15
slo.H128602	VAR2	28.75	237.43	51.80	57.98
slo.H118677	AT2G44500	1.02	0.11	0.27	0.42
slo.H102740	XLG3	16.68	23.37	37.95	26.34
slo.H127521	PETC	1.99	114.77	23.06	40.01
slo.H130477	CIB5	22.34	131.36	78.54	33.33
slo.H109165	AT1G02670	32.20	34.19	62.88	54.20
slo.H116841	GC1	18.74	31.76	29.98	31.29
slo.H116374	AT4G02425	14.63	23.83	30.90	17.01
slo.H109275	AT4G30020	2.26	0.33	1.00	0.55
slo.H126358	AT2G23093	8.83	16.31	19.64	22.53
slo.H124534	LOV1	0.24	9.88	0.00	0.58
slo.H128214	ABA3	98.10	77.46	51.07	17.18
slo.H127022	MBR1	78.59	126.10	97.12	91.22
slo.H110876	AT2G29290	22.45	38.19	29.02	36.69
slo.H118700	AT3G62310	71.00	74.67	51.71	53.85
slo.H127738	AT1G61850	23.99	17.88	40.41	24.82
slo.H122251	AT1G05670	7.44	21.88	17.80	14.49
slo.H106170	AT5G65490	17.88	18.50	27.81	24.91
slo.H122099	MOS7	18.64	64.44	31.18	50.42
slo.H124797	AT1G35660	8.16	16.75	21.71	13.10
slo.H108950	AT3G21810	17.83	46.71	56.34	45.20
slo.H111474	PDAT	15.68	0.16	0.16	0.27
slo.H124876	AT1G18340	24.34	15.11	30.98	17.71
slo.H102329	AT4G32820	7.05	11.92	13.84	14.51
slo.H122332	AT3G07700	28.61	52.88	37.35	38.45
slo.H105396	GR-RBP2	2.00	5.58	5.58	3.57
slo.H107826	EME1A	4.79	6.62	7.36	7.29
slo.H101027	AT2G45540	19.19	19.06	17.98	19.16
slo.H100829	PPH	0.11	0.97	0.63	0.06

slo.H114543	AT3G21465	5.97	7.68	14.54	8.29
slo.H120254	EDD1	10.06	15.72	21.82	15.91
slo.H115592	AT5G37360	16.77	102.43	30.89	57.46
slo.H123635	AT1G63080	10.87	18.76	24.82	15.01
slo.H107866	AT1G34150	8.50	21.64	26.86	24.78
slo.H117634	AT3G29280	8.53	7.20	32.75	17.01
slo.H114220	AT3G61080	10.35	25.71	17.95	17.85
slo.H121562	AT5G07770	28.35	22.70	26.31	22.91
slo.H130706	AT1G65270	83.49	92.41	103.33	90.10
slo.H107748	Fes1A	11.89	12.76	17.62	19.43
slo.H105238	THO2	21.78	31.52	37.74	26.79
slo.H120015	AT5G17250	8.34	5.71	11.65	9.91
slo.H105520	AT3G02820	0.11	0.00	0.00	0.38
slo.H112714	ALN	0.37	0.09	54.80	0.04
slo.H115855	AT3G23080	40.37	36.47	41.68	33.11
slo.H114387	AT1G59660	21.41	30.58	48.88	38.36
slo.H123626	AT1G25500	44.31	36.82	12.78	25.33
slo.H125920	SUA	45.36	73.20	73.07	63.66
slo.H112929	AT5G17670	9.87	76.36	48.40	37.38
slo.H121125	AT1G25420	40.29	37.93	31.45	46.94
slo.H100722	AT3G51050	49.65	83.36	85.64	66.64
slo.H103653	ATPI4K	39.90	62.78	79.08	66.42
slo.H117635	GEX3	0.61	0.00	0.05	0.00
slo.H112850	AT4G27390	8.27	49.61	42.37	36.51
slo.H112127	AT4G10600	35.87	60.13	76.67	58.95
slo.H116272	ERF11	0.00	0.00	0.10	0.00
slo.H105318	AT5G37530	4.11	6.29	12.52	4.22
slo.H115581	AT5G38890	22.97	38.61	76.14	38.19
slo.H119012	AT2G46560	9.16	15.49	31.27	21.86
slo.H121609	ASP5	58.25	71.85	90.89	72.04
slo.H117797	EDL2	34.02	67.49	88.04	61.06
slo.H121986	UGP3	7.20	18.25	20.93	8.04
slo.H110529	AT3G44050	0.27	0.82	3.48	2.27
slo.H100093	AT5G41060	1.12	5.64	1.89	3.83
slo.H116706	EDA7	17.77	26.59	28.85	23.21
slo.H107319	RECQSIM	1.82	8.01	8.82	11.68
slo.H117793	SAMC1	24.38	10.37	10.19	7.77
slo.H123772	AT2G32840	3.14	8.64	6.84	6.02
slo.H116114	AT1G03210	1.09	9.42	11.22	6.72
slo.H118581	AT5G63200	30.89	69.10	49.64	60.98
slo.H111700	AT5G06260	28.53	47.97	67.27	77.29
slo.H105544	AT1G09130	16.32	55.38	30.63	30.57
slo.H112008	RLT2	6.10	22.22	31.85	21.67
slo.H115792	AT1G63330	7.53	13.18	18.94	9.99

slo.H103390	AT4G31570	18.23	19.89	23.64	20.58
slo.H121402	AT5G20170	10.14	42.46	33.61	30.03
slo.H121939	SLP1	16.53	128.33	18.19	32.86
slo.H106643	LPA3	9.39	35.40	29.40	19.05
slo.H104069	AT5G39865	7.46	2.44	8.17	15.84
slo.H116961	AT5G06660	100.96	97.93	88.25	65.06
slo.H119827	TTN1	6.50	7.07	23.05	16.95
slo.H110528	OVA2	6.04	43.06	15.47	19.72
slo.H103911	AT4G27480	16.99	27.85	29.88	22.88
slo.H130161	NRPB9B	5.53	13.73	38.38	28.94
slo.H108444	AT4G19900	16.97	29.16	42.10	33.76
slo.H115999	AT3G46960	6.80	26.17	16.33	22.24
slo.H124417	LNO1	14.20	27.33	23.15	22.23
slo.H126685	AT4G31570	27.82	20.93	32.02	31.15
slo.H124764	PTP1	39.77	92.30	77.77	53.13
slo.H126042	NAC038	0.53	0.89	0.43	0.76
slo.H116967	AT3G17920	57.89	51.81	54.77	79.27
slo.H116579	AT4G01860	63.47	20.88	20.98	15.06
slo.H130666	AT3G07530	14.07	20.81	17.14	13.17
slo.H121161	AT4G29590	3.18	15.72	3.52	5.06
slo.H107724	CDKB1;2	0.38	0.48	5.39	2.38
slo.H112596	AT5G53920	2.83	6.22	3.27	3.20
slo.H129301	AT3G22845	148.41	125.78	143.81	103.24
slo.H118452	AT5G57230	3.80	8.37	11.80	7.59
slo.H123897	AT3G58470	17.99	17.19	33.26	21.45
slo.H114985	AT4G24090	13.41	59.34	23.55	33.23
slo.H105547	AT2G40640	22.95	25.16	32.24	30.68
slo.H103826	ZAT	11.52	19.55	21.55	14.54
